# Primary Prostatic Carcinoma with Metastasis to Epaxial Muscles and Myocardium in a Dog

**DOI:** 10.3390/vetsci12111045

**Published:** 2025-11-01

**Authors:** Hyouju Kim, Hyun-Jung Han, Dae Young Kim

**Affiliations:** 1 Department of Veterinary Emergency and Critical Care, College of Veterinary Medicine, Konkuk University, Seoul 05029, Republic of Korea; sdf4780@gmail.com; 2 KU Center for Animal Blood Medical Science, Konkuk University, Seoul 05029, Republic of Korea; 3 Veterinary Medical Diagnostic Laboratory, College of Veterinary Medicine, University of Missouri, Columbia, MO 65211, USA; kimdy@missouri.edu

**Keywords:** primary prostatic carcinoma, skeletal muscle metastasis, unusual metastatic pattern, comparative oncology, myocardial metastasis

## Abstract

Canine prostatic carcinoma is a highly aggressive neoplasm with a strong tendency to metastasize, most commonly to regional lymph nodes, lungs, and bones. However, skeletal muscle and myocardial involvement are rarely reported. This report describes an 11-year-old intact male Maltese dog with a two-month history of anorexia and lethargy, referred for further evaluation after failing to respond to piroxicam therapy. Diagnostic imaging revealed a prostatic mass and multiple rim-enhancing lesions in the epaxial musculature and left ventricular wall, without evidence of metastasis to the lymph nodes, lungs, or other visceral organs. Hemostatic analysis indicated a hypercoagulable state. Postmortem examination confirmed metastatic prostatic carcinoma involving the semispinalis, multifidus, and myocardium. Histologically, the neoplastic cells exhibited similar morphology at the primary and metastatic sites. Immunohistochemistry revealed strong cytokeratin expression and absence of uroplakin III, consistent with a non-urothelial epithelial origin. No evidence of lymphatic involvement was observed. To the best of our knowledge, this is the first documented case of canine prostatic carcinoma with exclusive myotropic and myocardial metastases. These findings suggest a possible hematogenous metastatic route and highlight the importance of including muscle and cardiac tissues in staging protocols for canine prostatic carcinoma, even when lymphadenopathy or pulmonary lesions are absent.

## Simple Summary

Prostate cancer in dogs is rare but often aggressive, with a tendency to spread to other parts of the body. It typically occurs in older male dogs and commonly spreads to the lungs, bones, or nearby lymph nodes. In this case, a small-breed dog exhibited fatigue and decreased appetite for 2 months. Despite medical treatment, the dog was euthanized. A detailed examination revealed that the cancer had spread only to the muscles along the spine and to the heart walls. The affected muscles included those near the neck and lower back. There was no evidence of cancer in the lungs, lymph nodes, or internal organs, which are commonly affected by this oncological disease. Prostate cancer spreading to the muscles and heart tissue is extremely rare in animals and humans. This case may provide insight into unusual patterns of cancer spread in dogs.

## 1. Introduction

Canine prostatic carcinoma is a highly aggressive malignant neoplasm that predominantly affects older neutered male dogs [1,2]. Histological subtypes may include urothelial, adenocarcinomatous, and mixed phenotypes; however, the tumor most often arises from the epithelial components of the prostate [3]. Histopathological heterogeneity is frequently observed, encompassing a range of architectural patterns such as glandular, solid, papillary, and cribriform arrangements, which reflect varying degrees of differentiation and biological behavior [4].

Prostatic carcinomas in dogs exhibit a high propensity for metastasis at diagnosis and throughout disease progression. The most commonly reported metastatic sites include the regional iliac and sublumbar lymph nodes, lungs, and axial skeleton, particularly the lumbar vertebrae and pelvis [5]. Less frequently, metastatic spread has been documented in the liver, spleen, kidneys, lungs, gastrointestinal tract, brain, and adrenal glands [5,6,7,8,9,10,11,12,13].

The precise mechanisms underlying metastasis in canine prostatic carcinoma remain incompletely understood [14]. However, hematogenous and lymphatic routes are thought to play significant roles in neoplastic cells’ dissemination [15]. Hematogenous spread may explain distant organ involvement, particularly in the bones, whereas lymphatic metastasis typically accounts for nodal involvement [16,17,18]. Aggressive local invasion into adjacent structures, including the bladder, urethra, and rectum, has also been reported [13].

Metastasis to skeletal muscle is extremely rare in dogs, and, to our knowledge, cardiac involvement in prostatic carcinoma has not been previously reported. This case report describes a unique presentation of canine prostatic carcinoma with isolated metastases to the epaxial skeletal muscles and myocardium, without detectable spread to the lymphatic system or visceral organs. Based on current literature, this represents the first documented case of canine prostatic carcinoma with exclusive myotropic metastasis.

## 2. Case Presentation

### 2.1. Clinical Examination

An 11-year-old intact male Maltese dog presented with a two-month history of anorexia and lethargy. The patient had a prior diagnosis of urinary bladder and prostatic tumors at a local veterinary clinic and was treated with piroxicam; however, no significant clinical improvement was noted. Owing to the lack of therapeutic response, the dog was referred to Konkuk University Veterinary Medical Teaching Hospital for further diagnostic evaluation and case management. The patient had a history of myxomatous mitral valve disease (MMVD) and was receiving cardiac medication. The physical examination revealed normal urination and defecation, and vital signs (heart rate, body temperature, and blood pressure) were within reference ranges.

### 2.2. Laboratory Findings

Hematological evaluation revealed mild, non-regenerative anemia (packed cell volume, 34.3%; reference interval [RI], 37–55%) (Table 1). Serum biochemistry revealed marked increases in blood urea nitrogen (80 mg/dL; RI: 8–26 mg/dL), creatinine (2.2 mg/dL; RI: 0.5–1.8 mg/dL), and symmetric dimethylarginine (48 µg/dL; RI: 0–14 µg/dL). Inflammatory markers were also elevated, with C-reactive protein at 5 mg/dL (RI < 1 mg/dL). D-dimer concentration was significantly increased (2436.69 ng/mL; RI: 0–250 ng/mL), indicating a potential prothrombotic state. Thromboelastography revealed a shortened R time (3.9 min; RI: 5–10 min) and K time (0.8 min; RI: 1–3 min), with increased α angle (79.3°; RI: 53–72°), maximum amplitude (81.3 mm; RI: 50–70 mm), and clot strength (G, 21.8 K d/sc; RI: 4.6–10.9 K d/sc), consistent with accelerated clot initiation and enhanced clot firmness. Lysis at 30 min (0%; RI: 0–8%) and estimated percent lysis (0%; RI: 0–15%) were within normal limits, indicating no evidence of fibrinolysis. With the markedly elevated D-dimer level, the coagulation profile indicated a hypercoagulable state.

### 2.3. Diagnostic Imaging

Abdominal radiographs showed dorsal displacement of the descending colon and increased soft tissue opacity in the mesenteric region of the urinary bladder. Abdominal ultrasonography revealed a heterogeneous, hypoechoic mass (51.8 × 42.4 mm) in the dorsoventral bladder wall, containing hyperechoic debris and multiple anechoic foci. The prostatic urethra was visualized traversing the mass, and a small volume of anechoic abdominal effusion was noted cranial to the lesion. The mass compressed the urinary bladder and descending colon, with hyperechoic changes observed in the adjacent peritoneum.

Echocardiography was performed following the detection of a cardiac murmur on auscultation. No radiographic evidence of cardiac hypertrophy or pulmonary abnormalities was observed. The examination revealed mild mitral regurgitation with chordal rupture and degenerative changes of the mitral valve. Lesions with a tram-line appearance were identified in the main pulmonary artery; however, there was no evidence of pulmonary artery dilatation or pulmonic valve obstruction. A structure consistent with a heartworm was observed on echocardiography, but the heartworm antigen test was negative, suggesting the possibility of a male-only infection.

**Figure 1:**
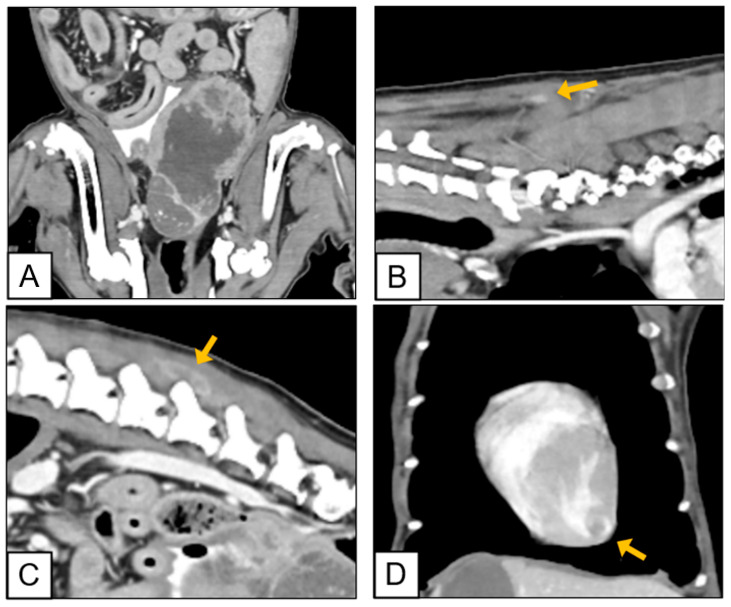
Computed tomography (CT) images of metastatic lesions in the prostate, epaxial musculature, and heart. CT imaging revealed an asymmetrically enlarged left prostatic lobe with internal mineralization and displacement of adjacent organs (**A**). Rim-enhancing lesions, suggestive of muscular metastases, were identified in the semispinalis and multifidus muscles at C7 (16 × 4 × 3.2 mm) (**B**) and in the epaxial musculature at L4–L5 (26 × 10 × 5 mm) and L6 (15 × 8 × 4 mm) (**C**). Cardiac metastases included a large mass in the left ventricular wall (31 × 23 × 10 mm) and two adjacent nodules (23 × 10.5 × 5.9 mm and 14.3 × 10.4 × 3.3 mm) (**D**). Arrows indicate metastatic lesions in panels (**B**–**D**). No gross abnormalities were observed in the thoracic duct or sublumbar lymph nodes.

Computed tomography (CT) provided further characterization, revealing asymmetrical enlargement of the left prostatic lobe, displacement of the bladder and colon, and internal parenchymal mineralization suggestive of malignancy (Figure 1A). No metastatic lesions were detected in the lungs, bones, or regional lymph nodes; however, multiple rim-enhancing lesions were observed in the epaxial musculature and left ventricular myocardium. Firm, white metastatic masses were identified in the semispinalis and multifidus muscles at cervical vertebra C7 (16 × 4 × 3.2 mm) (Figure 1B), lumbar vertebrae L4–L5 (26 × 10 × 5 mm), and L6 (15 × 8 × 4 mm) (Figure 1C). Myocardial metastases included a large mass within the left ventricular wall (31 × 23 × 10 mm) and additional nodules in close proximity (23 × 10.5 × 5.9 mm; 14.3 × 10.4 × 3.3 mm) (Figure 1D). No gross abnormalities were noted in the thoracic duct or sublumbar lymph nodes, which measured 9 × 7 mm, 9 × 10 mm, and 6 × 10 mm.

### 2.4. Treatment and Outcome

The patient was maintained on cardiac medications and diuretics—including furosemide, amlodipine, pimobendan, and spironolactone—for management of pre-existing MMVD. However, owing to progressive clinical deterioration, poor prognosis, and financial constraints, humane euthanasia was performed with the owners’ consent, followed by necropsy.

### 2.5. Necropsy Findings

**Figure 2:**
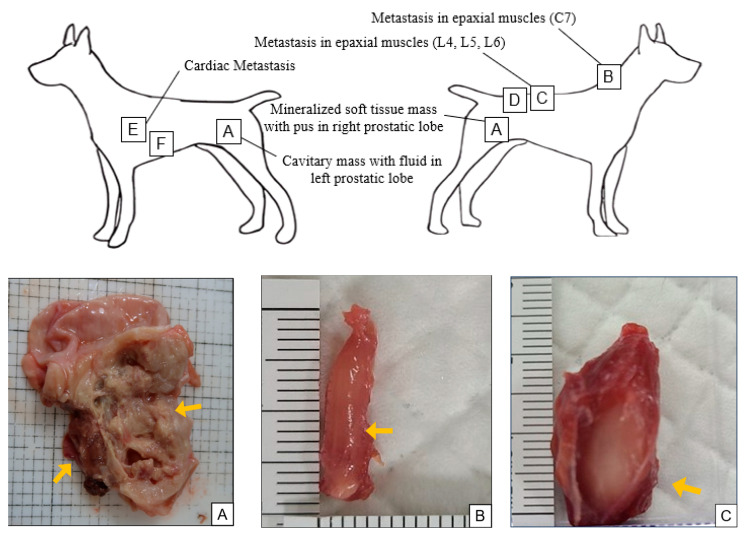

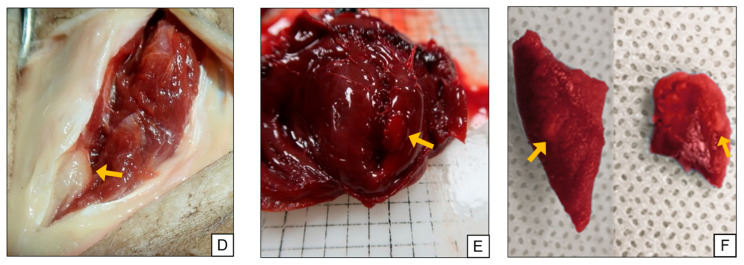
Gross pathological findings at necropsy. A schematic diagram (top) illustrates the anatomical sites of the primary and metastatic lesions labeled A–F. Grossly, a cavitary, fluid-filled mass was identified in the left prostatic lobe (right arrow), and a mineralized soft tissue mass containing purulent material was observed in the right prostatic lobe (left arrow) (**A**). Multiple firm, white metastatic nodules were present in the epaxial musculature at C7 (**B**), L4–L5 (**C**), and L6 (**D**), corresponding to imaging findings. Cardiac metastases were observed in the left ventricular wall, including a large mass measuring 31 × 23 × 10 mm (**E**) and two adjacent nodules measuring 23 × 10.5 × 5.9 mm (left arrow) and 14.3 × 10.4 × 3.3 mm (right arrow), respectively (**F**). No gross abnormalities were observed in the thoracic duct or lymph nodes. Additional necropsy photographs are provided in the Supplementary Materials.

Gross pathology at *post-mortem* examination revealed a cavitary, fluid-filled mass in the left prostatic lobe and a mineralized soft tissue mass containing purulent material in the right lobe (Figure 2A). Multiple white, firm metastatic nodules were identified in the epaxial musculature, corresponding to imaging findings at C7, L4–L5, and L6 (Figure 2B–D). Cardiac metastases were also observed in the left ventricular wall, including a 31 × 23 × 10 mm mass (Figure 2E) and two adjacent nodules measuring 23 × 10.5 × 5.9 mm and 14.3 × 10.4 × 3.3 mm, respectively (Figure 2F). No other congenital malformations or structural abnormalities were observed. The thoracic ducts and lymph nodes showed no significant gross abnormalities, and no additional metastases were identified in other organs.

### 2.6. Histopathological and Immunohistochemical Findings

**Figure 3:**
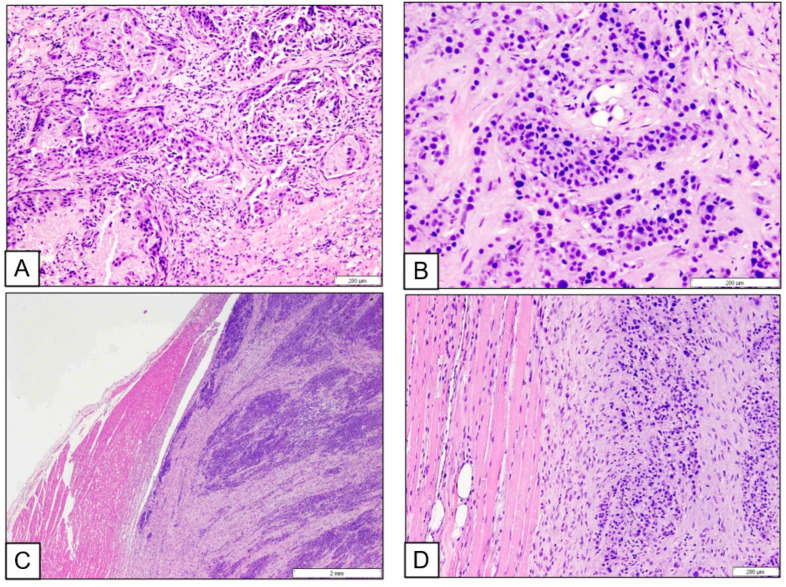
The prostate gland contained an ill-defined, infiltrative carcinoma composed of polyhedral epithelial cells forming irregularly shaped glands (**A**) and cellular trabeculae and islands (**B**). A metastatic lesion is visible on the right side of the image, infiltrating the left ventricular myocardium (**C**). A similar metastatic focus is seen on the right side of the image within the multifidus muscle at L4 (**D**). Both metastatic sites exhibited architectural patterns and cytoplasmic features consistent with the primary tumor, most notably moderate eosinophilic cytoplasm.

Tissue specimens were collected from the prostate glands, epaxial muscles, myocardium, and urinary bladder; fixed in 10% neutral-buffered formalin; routinely processed; and stained with hematoxylin and eosin for histopathological evaluation. Immunohistochemistry was conducted to determine tumor origin using antibodies against cytokeratin (clone AE1/AE3, 1:200; Dako) and uroplakin III (BSB 2289, 1:100; Bio SB). Histologically, both prostate glands contained ill-defined, locally invasive epithelial neoplasms composed of neoplastic polyhedral cells arranged in glandular, tubular, trabecular, and small island patterns. In certain regions, tumor cells were individually dispersed. The neoplastic cells exhibited round nuclei, moderate eosinophilic cytoplasm, and indistinct cytoplasmic borders. A mitotic count of seven per ten high-power fields (400×) was recorded. Metastatic lesions with similar histomorphological features were observed in the semispinalis muscle at C7, the multifidus muscle at L4–L6, the left ventricular myocardium, and the trigone region of the urinary bladder (Figure 3). No metastatic involvement was detected in the sublumbar lymph nodes, including the internal and medial iliac nodes.

**Figure 4:**
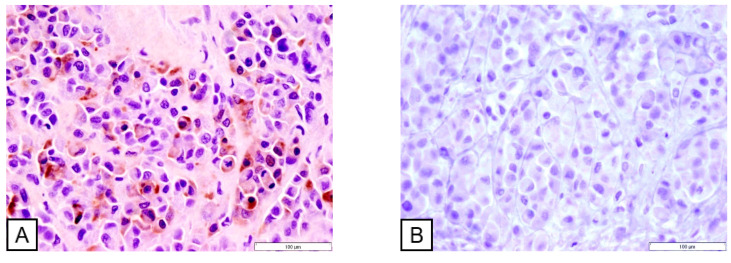
Immunohistochemistry. The prostate tumor cells were positive for cytokeratin (**A**) and negative for uroplakin III (**B**).

Immunohistochemically, the tumor cells in the prostate, skeletal muscle, and myocardium demonstrated strong cytoplasmic immunoreactivity for cytokeratin, but lacked the expression of uroplakin III, a marker specific for urothelial differentiation (Figure 4).

## 3. Discussion

Canine prostatic carcinoma is a highly aggressive cancer, with gross metastases observed in approximately 80% of cases at necropsy [1]. Common metastatic sites include regional lymph nodes, lungs, and bones. Additionally, metastases to visceral organs, such as the liver, kidneys, adrenal glands, colon, and spleen, have also been documented in veterinary and human studies [1,5,6,7,8,9,10,11,12,13]. A retrospective necropsy study of 76 dogs with confirmed prostate carcinoma found that 80% exhibited metastatic disease, most commonly involving the lymph nodes, lungs, and bones [1]. Conversely, skeletal muscle and cardiac tissue involvement is extremely rare, infrequently reported in veterinary literature, and often overlooked in clinical assessments. In one study that examined primary and secondary cardiac tumors in dogs and cats, metastatic lesions were identified in 24 of 66 canine cases, most commonly originating from carcinomas, malignant lymphomas, and hemangiosarcomas [19]. This finding underscores the rarity of cardiac metastasis, particularly from prostatic carcinoma.

Hematologic and hemostatic findings indicate systemic inflammation, azotemia, and a paraneoplastic coagulopathy. The mild non-regenerative anemia is consistent with anemia of chronic disease, a syndrome frequently observed in dogs with neoplasia and inflammation, where cytokine-mediated iron sequestration and suppressed erythropoiesis play key roles [15,20]. The concurrent azotemia and elevated SDMA suggest early renal involvement, which may be functional secondary to systemic inflammation, or postrenal, due to urethral or prostatic obstruction. The raised CRP indicates the presence of systemic inflammation [21]. The coagulation profile, marked elevation of D-dimer with TEG evidence of shortened clotting times and increased clot strength, indicates a hypercoagulable state, which has been documented in dogs with neoplastic disease [22]. Such a paraneoplastic prothrombotic milieu may facilitate metastatic spread by enabling tumor cells to interact with platelets, seed microthrombi, and extravasate into vascularized tissues more readily [23].

Bone metastases, particularly to the lumbar vertebrae and pelvis, are observed in approximately 22% of dogs with necropsy-confirmed cases [1]. However, ante-mortem detection is often limited because of imaging sensitivity or the lack of targeted sampling [24]. In the present case, although bone histopathology was not performed, the absence of CT-detected bone lesions, along with the exclusive identification of metastatic lesions in the epaxial musculature and myocardium, suggests the possibility of isolated muscular and cardiac metastases [25]. Similar occurrences have been reported in human oncology, where muscular metastases developed independently of skeletal involvement [26,27,28].

The mechanisms underlying the rarity of skeletal muscle metastases, especially in isolation, remain unclear. Skeletal muscle is generally regarded as an inhospitable environment for metastatic colonization. Hypotheses to explain this resistance include physical barriers imposed by muscle motion, high local lactic acid concentrations, and the potential for muscle fibroblasts to encapsulate neoplastic cells [29]. Another proposed mechanism involves adenosine receptors, with muscle cells releasing agonists in vitro that may exert systemic anticancer and chemoprotective effects [30]. Recent studies have also highlighted structural, metabolic, and immunological factors such as mechanical barriers, vascular shear forces, extracellular matrix stiffness, myokine secretion, and exercise-induced metabolic shifts that may further restrict tumor colonization of skeletal muscle [31].

The anatomical and hemodynamic characteristics of venous circulation may partially explain the unusual metastatic pattern observed in this case. In humans, Batson’s venous plexus is a recognized valveless venous network that allows retrograde blood flow and provides a pathway for metastatic spread from the pelvis to the vertebral column and thoracic organs [32]. In dogs, the internal vertebral venous plexus (IVVP) serves an equivalent role, forming bidirectional connections between the deep pelvic and thoracic venous systems, including those draining the prostate, urinary bladder, and mammary glands [33]. Because these veins lack valves, blood and circulating tumor cells can flow in either direction depending on local pressure gradients [33]. This mechanism may explain the hematogenous dissemination of prostatic carcinoma cells to the epaxial skeletal muscles, which are adjacent to the vertebral venous plexus.

Metastasis to the myocardium cannot be fully explained by the IVVP pathway, as the cardiac venous system is not directly connected to this network. A possible route might be systemic hematogenous dissemination, whereby circulating tumor cells enter the coronary circulation and become lodged within cardiac chambers [34,35]. Nevertheless, cardiac muscle metastases remain exceptionally rare in human oncology, reflecting the combined effects of dynamic contractile activity, continuous blood flow, and a lymphatic architecture that hinders tumor cell implantation and growth [34,35]. When cardiac involvement occurs, it is often detected incidentally during advanced imaging or postmortem examination, as most cases are clinically silent [34]. In dogs, reported myocardial metastases have been associated primarily with melanoma, lymphoma, and sarcoma, whereas those originating from prostatic carcinoma are exceedingly uncommon [35].

The absence of lymph node or visceral organ involvement in this case is particularly noteworthy. Prostatic carcinoma typically spreads via lymphatic and hematogenous routes, with nodal metastasis often preceding distant organ involvement [1]. In canine prostatic carcinoma, a multicenter retrospective CT series (*n* = 30) reported lymph node enlargement, but none documented muscular metastases [36]. On the other hand, the findings in this case suggest the possibility of exclusive hematogenous dissemination, resulting in isolated metastases to highly vascularized muscle groups and the myocardium. This route has been postulated in several human and veterinary reports describing muscular and cardiac metastases from urogenital tumors [37,38,39].

Moreover, this case underscores the diagnostic value of advanced imaging modalities, particularly contrast-enhanced CT, for detecting clinically silent or atypical metastatic lesions. In small-animal practice, similar CT findings have supported the use of whole-body scans to identify occult metastases in urothelial and other neoplasms [40,41]. In a cohort of 15 dogs with confirmed skeletal muscular metastases, lesions appeared as well-defined, oval-to-round nodules with heterogeneous post-contrast enhancement, including ring-enhancing patterns [42]. In the present case, comparable rim-enhancing lesions were observed in the skeletal muscle and myocardium. Comprehensive whole-body imaging is often underutilized in routine staging protocols for prostatic carcinoma in veterinary practice; however, this case demonstrates that contrast-enhanced CT can reveal occult lesions not apparent on physical examination or standard radiography, including those in muscle and cardiac tissues.

The presence and distribution of metastases at diagnosis are key prognostic indicators in dogs with prostatic carcinoma. Reported median survival times range from 0–6.9 months in dogs with metastatic disease, depending on the treatment modality [5,43]. While non-steroidal anti-inflammatory drug therapy alone may modestly prolong survival, combination protocols incorporating chemotherapy (e.g., mitoxantrone or carboplatin) and advanced radiation therapy (e.g., Intensity-modulated radiation therapy) have demonstrated improved outcomes in select cases [44]. In this case, however, the extent of the disease, poor clinical response, and owner-related limitations precluded aggressive interventions.

## 4. Conclusions

In summary, this case report expands the recognized spectrum of metastatic behavior in canine prostate carcinoma to include the cardiac and skeletal muscles broadening the known spectrum of affected tissues. The identification of exclusive metastases to the skeletal muscle and myocardium, in the absence of lymph node or visceral involvement, challenges the conventional paradigm of metastatic progression in this disease. These findings suggest that hematogenous dissemination to muscle-rich vascular beds may occur more frequently than previously recognized. They also emphasize the importance of comprehensive diagnostic imaging, including cardiac and muscular assessments, even when lymphadenopathy or pulmonary lesions are not present. Clinicians should consider atypical metastatic presentations in muscle and cardiac tissues during staging and monitoring protocols. Future studies should aim to elucidate the mechanisms underlying these rare metastatic patterns.

## Figures and Tables

**Table 1:** Hematologic, biochemical, and hemostatic test results on presentation.

	Results	Unit	Reference Interval	Parameters
HCT	34.3	%	37–55%	↓
WBC	14.46	×10^3^/µL	6–17 × 10^3^/µL	-
MCV	60.9	fL	60–74 fL	-
MCHC	35.6	g/dL	31–36 g/dL	-
PLT	375	×10^3^/µL	200–500 × 10^3^/µL	-
BUN	80	mg/dL	8–26 mg/dL	↑
Creatinine	2.2	mg/dL	0.5–1.8 mg/dL	↑
SDMA	48	µg/dL	0–14 µg/dL	↑
CRP	5	mg/dL	<1 mg/dL	↑
D-dimer	2436.69	ng/mL	0–250 ng/mL	↑
TEG R	3.9	min	5–10 min	↓
TEG K	0.8	min	1–3 min	↓
α angle	79.3	°	53–72°	↑
MA	81.3	mm	50–70 mm	↑
LY30	0	%	0–8%	-
EPL	0	%	0–15%	-

Reference intervals (RIs) are based on institutional standards. Arrows indicate whether values are within or outside RI: ↑: above, -: within, ↓: below. PCV, packed cell volume; BUN, blood urea nitrogen; SDMA, symmetric dimethylarginine; CRP, C-reactive protein; MCV, mean cell volume; MCHC, mean corpuscular hemoglobin concentration; PLT, platelet count; WBC, white blood cell count; MA, maximum amplitude; G, clot strength; LY30, lysis at 30 min; EPL, estimated percent lysis; TEG, thromboelastography.

## Data Availability

The original contributions presented in this study are included in the article/Supplementary Material. Further inquiries can be directed to the corresponding author(s). Supplementary Materials, including MRI and CT scans, clinical chemistry results, and necropsy photographs, have been provided to support the findings of this case report.

## Supplementary Materials

The following supporting information can be downloaded at: https://www.mdpi.com/article/10.3390/vetsci12111045/s1, File S1: MRI and CT scans, clinical chemistry results, and necropsy photographs.

## Abbreviations

The following abbreviations are used in this manuscript:

BUN	Blood urea nitrogen
CRP	C-reactive protein
CT	Computed tomography
H&E	Hematoxylin and eosin
IHC	Immunohistochemistry
MMVD	Myxomatous mitral valve disease
PCV	Packed cell volume
RI	Reference interval
SDMA	Symmetric dimethylarginine
TEG	Thromboelastography
R	Reaction time (TEG parameter)
K	Clot formation time (TEG parameter)
α angle	Clot acceleration angle (TEG parameter)
MA	Maximum amplitude (TEG parameter)
G	Clot strength (TEG parameter)
LY30	Lysis at 30 min (TEG parameter)
EPL	Estimated percent lysis (TEG parameter)
