# Supplementary figures and images for: Primary Prostatic Carcinoma with Metastasis to Epaxial Muscles and Myocardium in a Dog (part 2 of 2)
Source: Vet Sci. 2025 Nov 1;12(11):1045. doi: 10.3390/vetsci12111045 (PMC12656942; doi:10.3390/vetsci12111045)

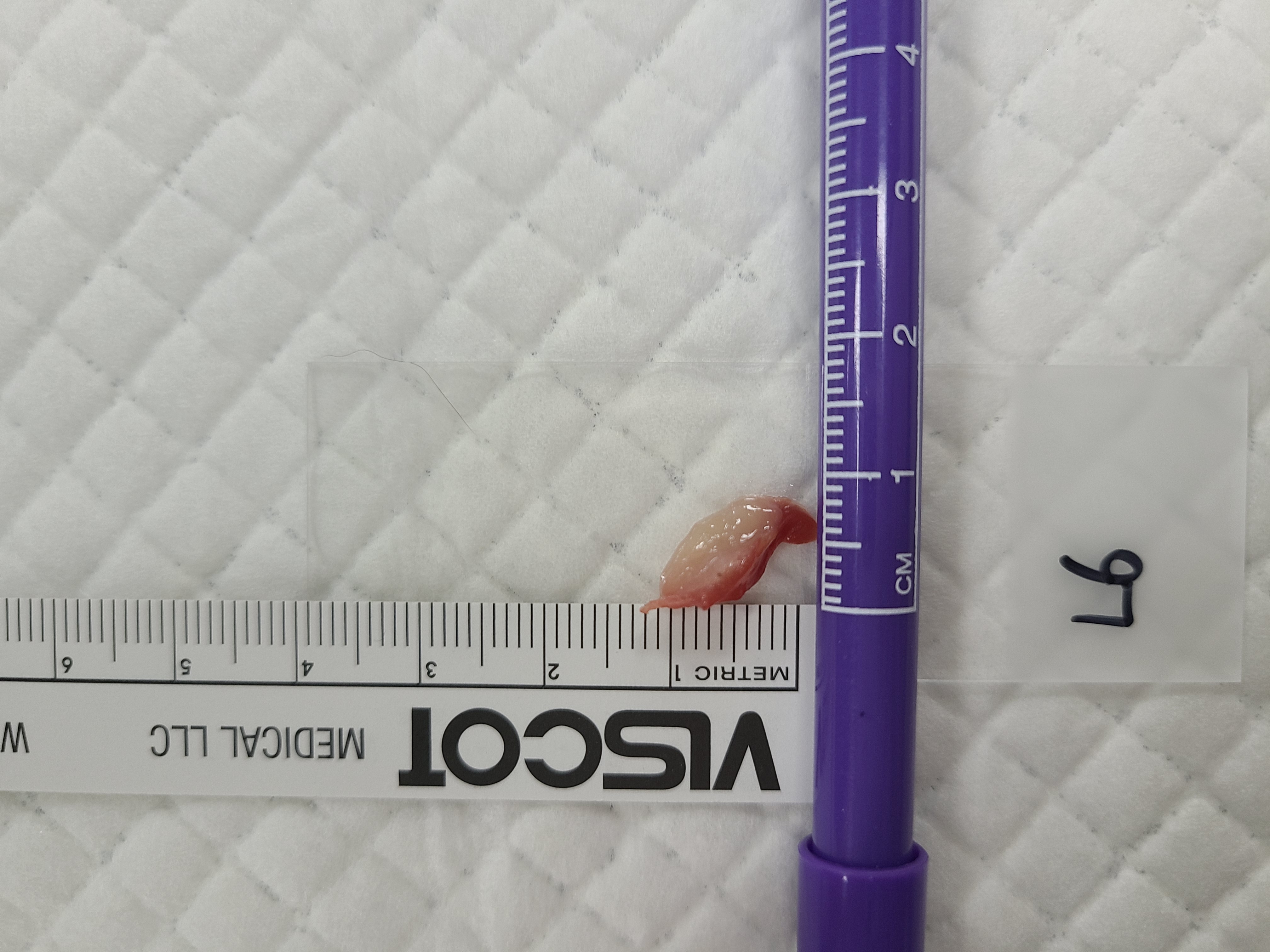

Supplement: Supplementary file 1 [file vetsci-12-01045-s001.zip › KakaoTalk_20221022_173638675_10.jpg]

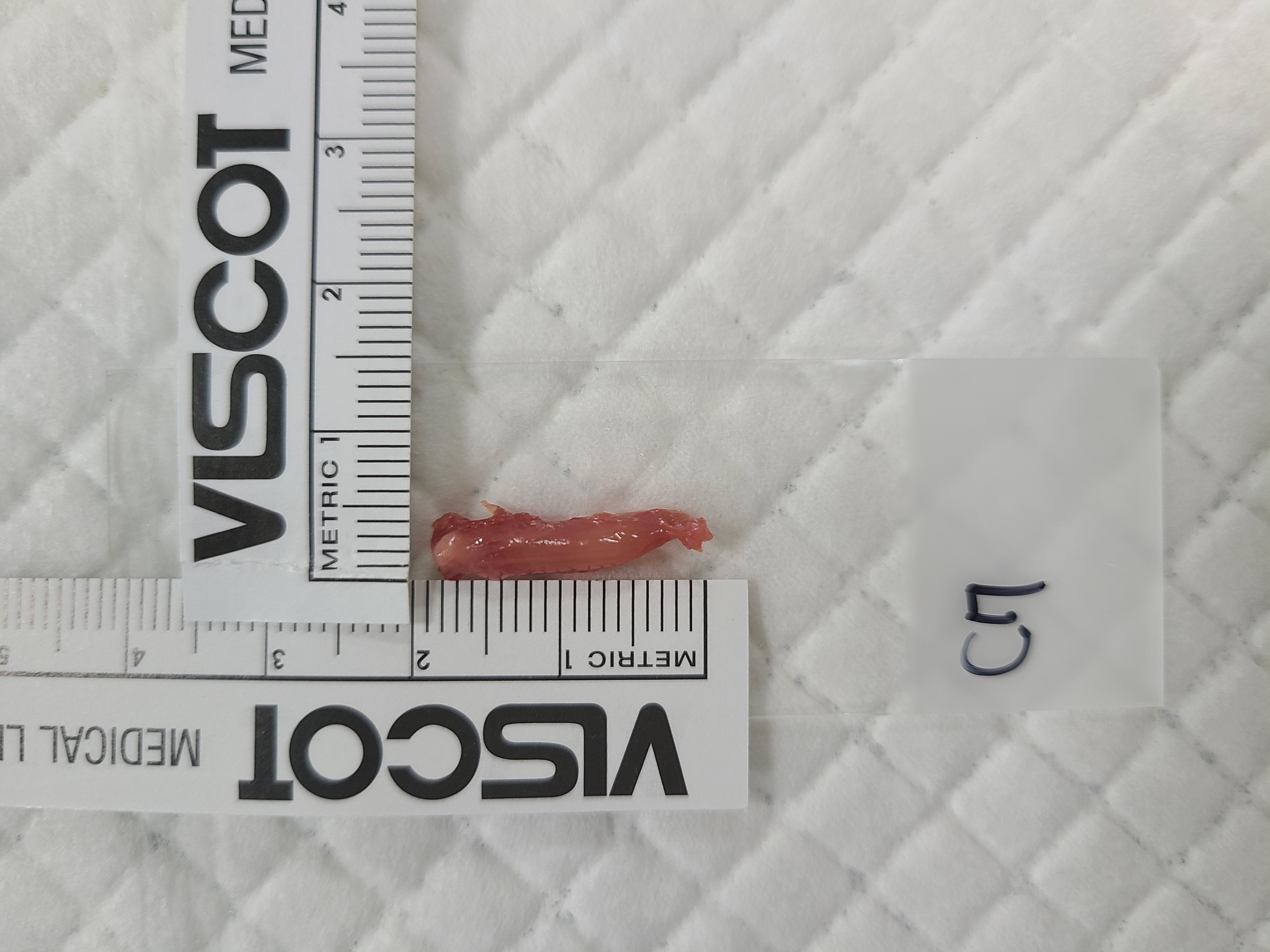

Supplement: Supplementary file 1 [file vetsci-12-01045-s001.zip › KakaoTalk_20221022_173638675_11.jpg]

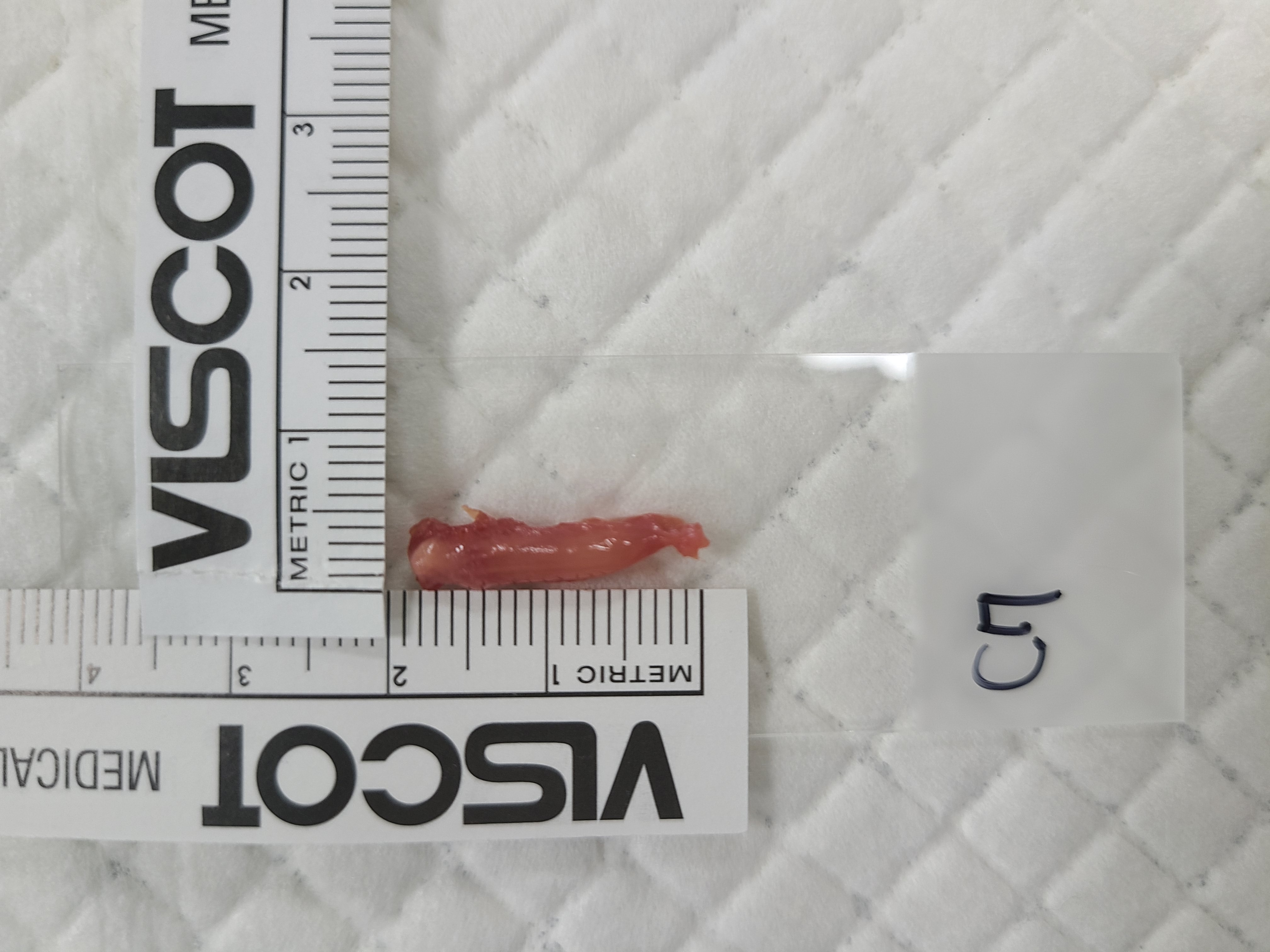

Supplement: Supplementary file 1 [file vetsci-12-01045-s001.zip › KakaoTalk_20221022_173638675_12.jpg]

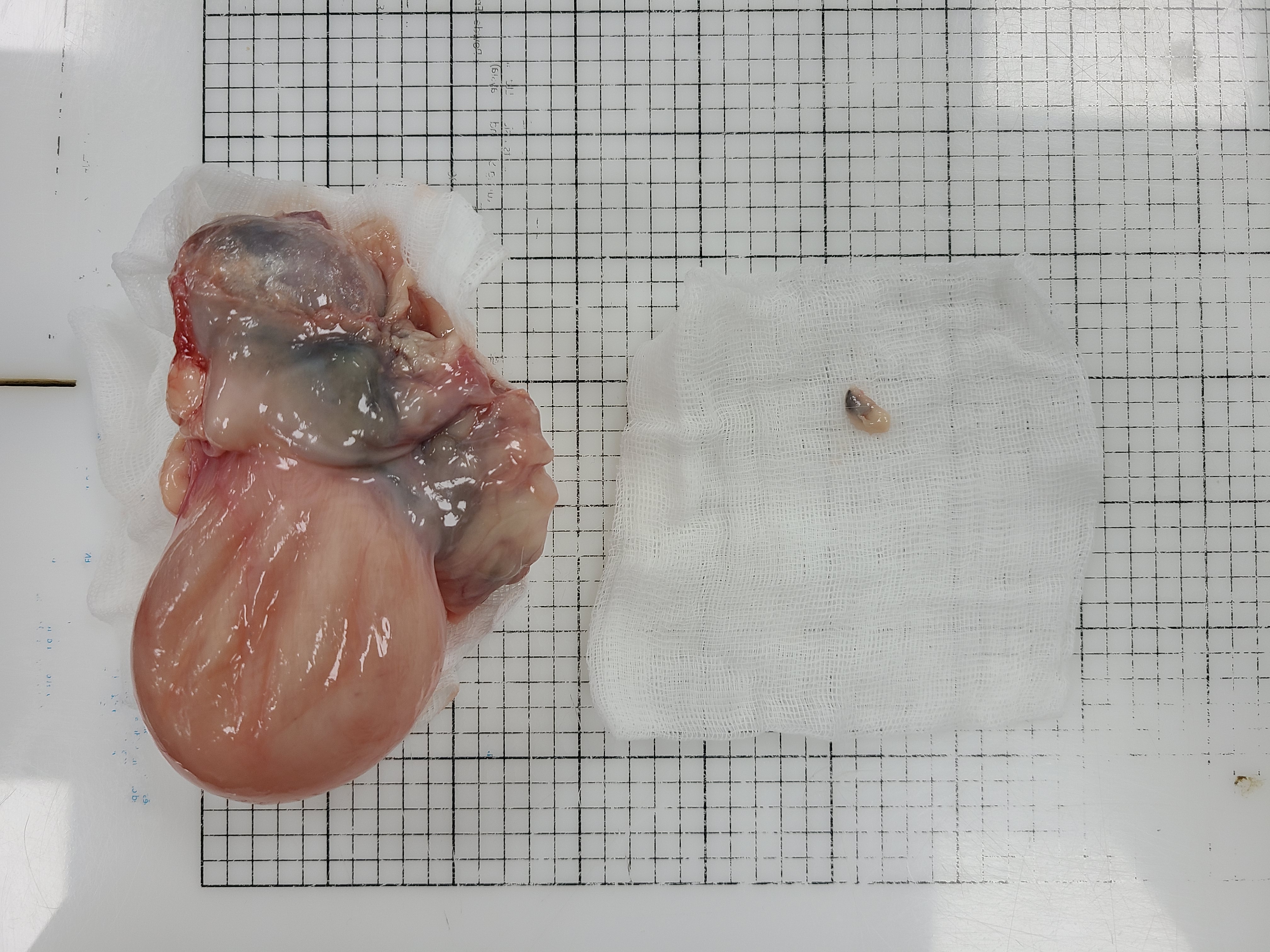

Supplement: Supplementary file 1 [file vetsci-12-01045-s001.zip › KakaoTalk_20221022_173638675_13.jpg]

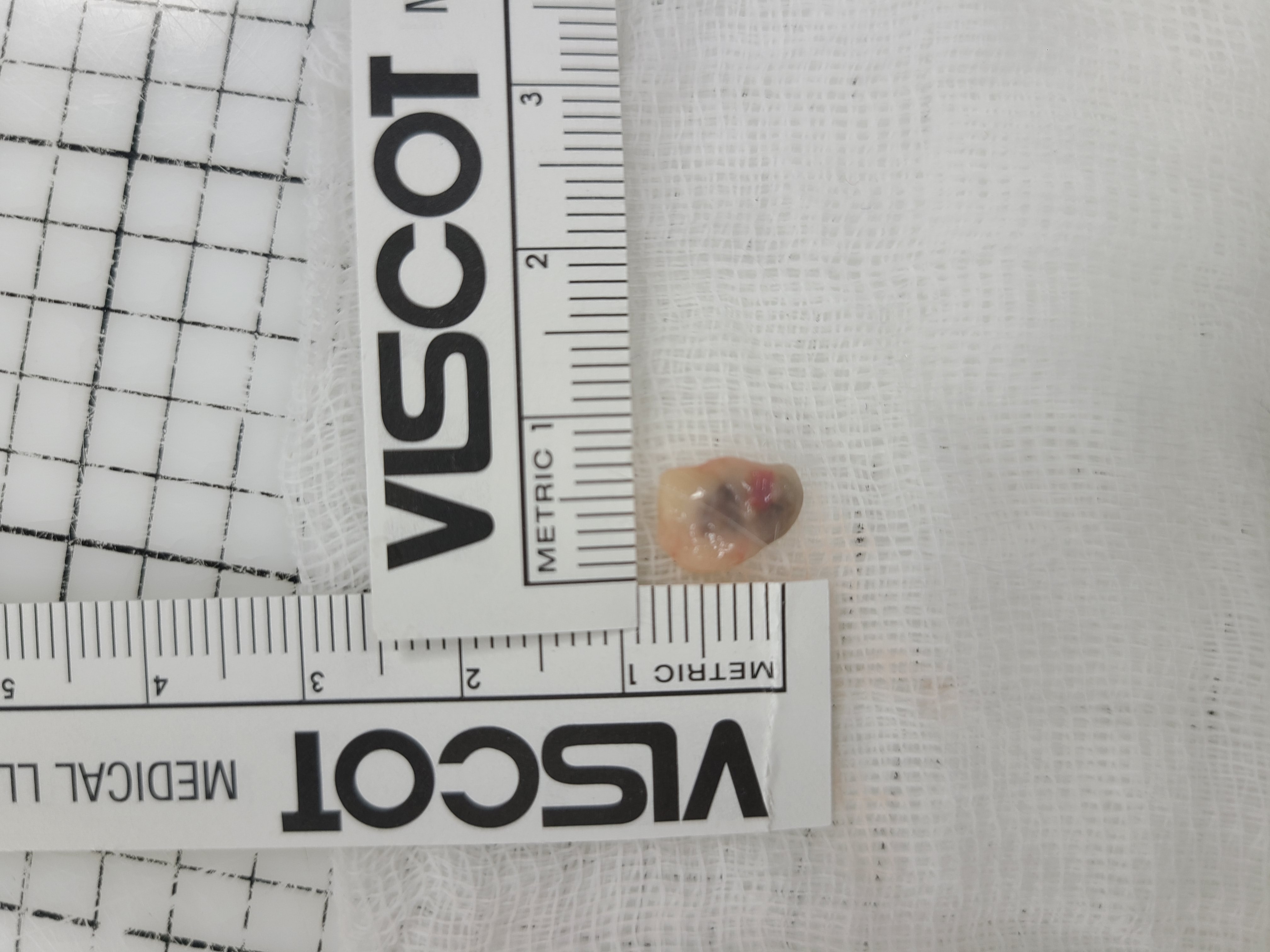

Supplement: Supplementary file 1 [file vetsci-12-01045-s001.zip › KakaoTalk_20221022_173638675_14.jpg]

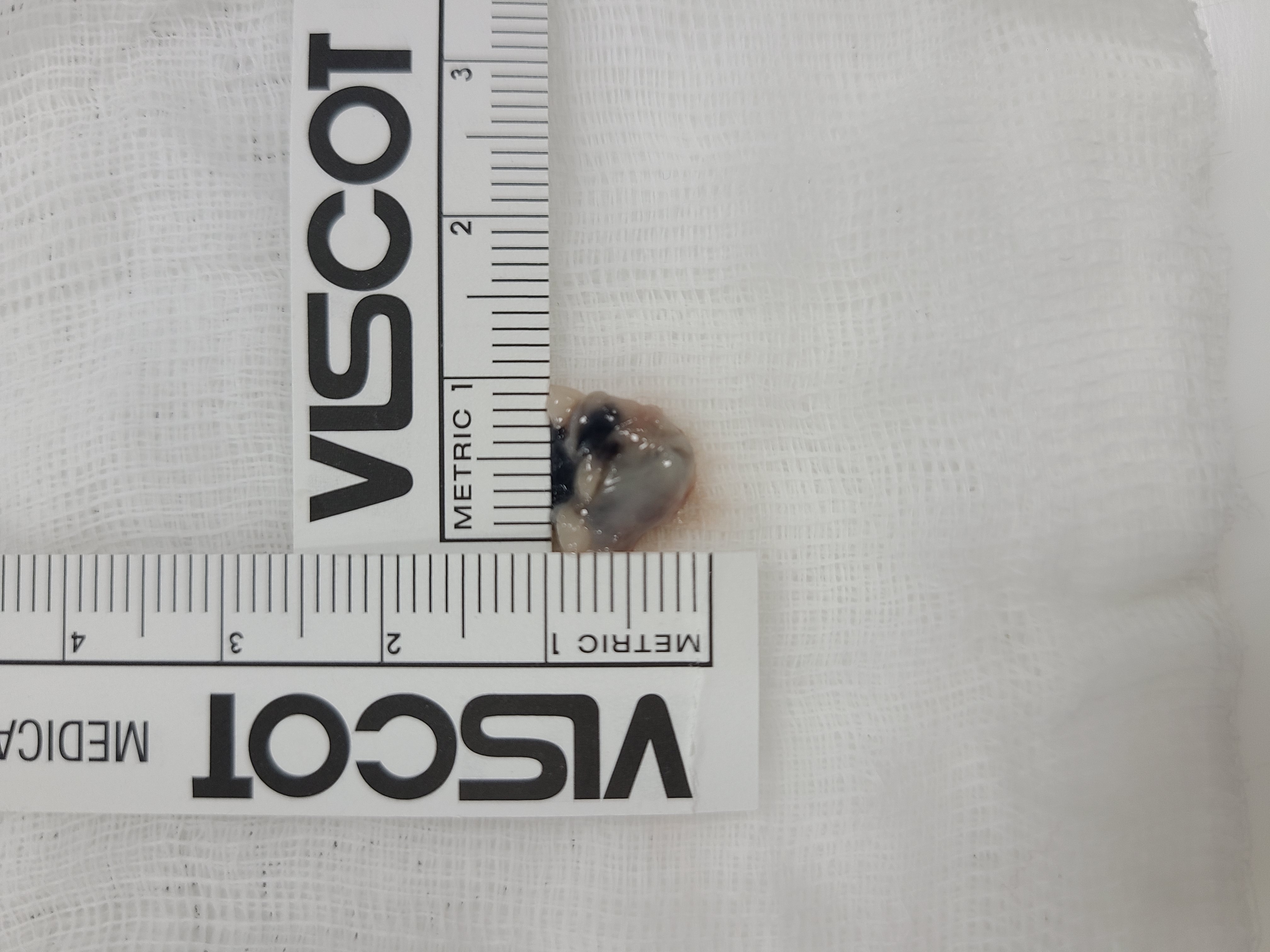

Supplement: Supplementary file 1 [file vetsci-12-01045-s001.zip › KakaoTalk_20221022_173638675_15.jpg]

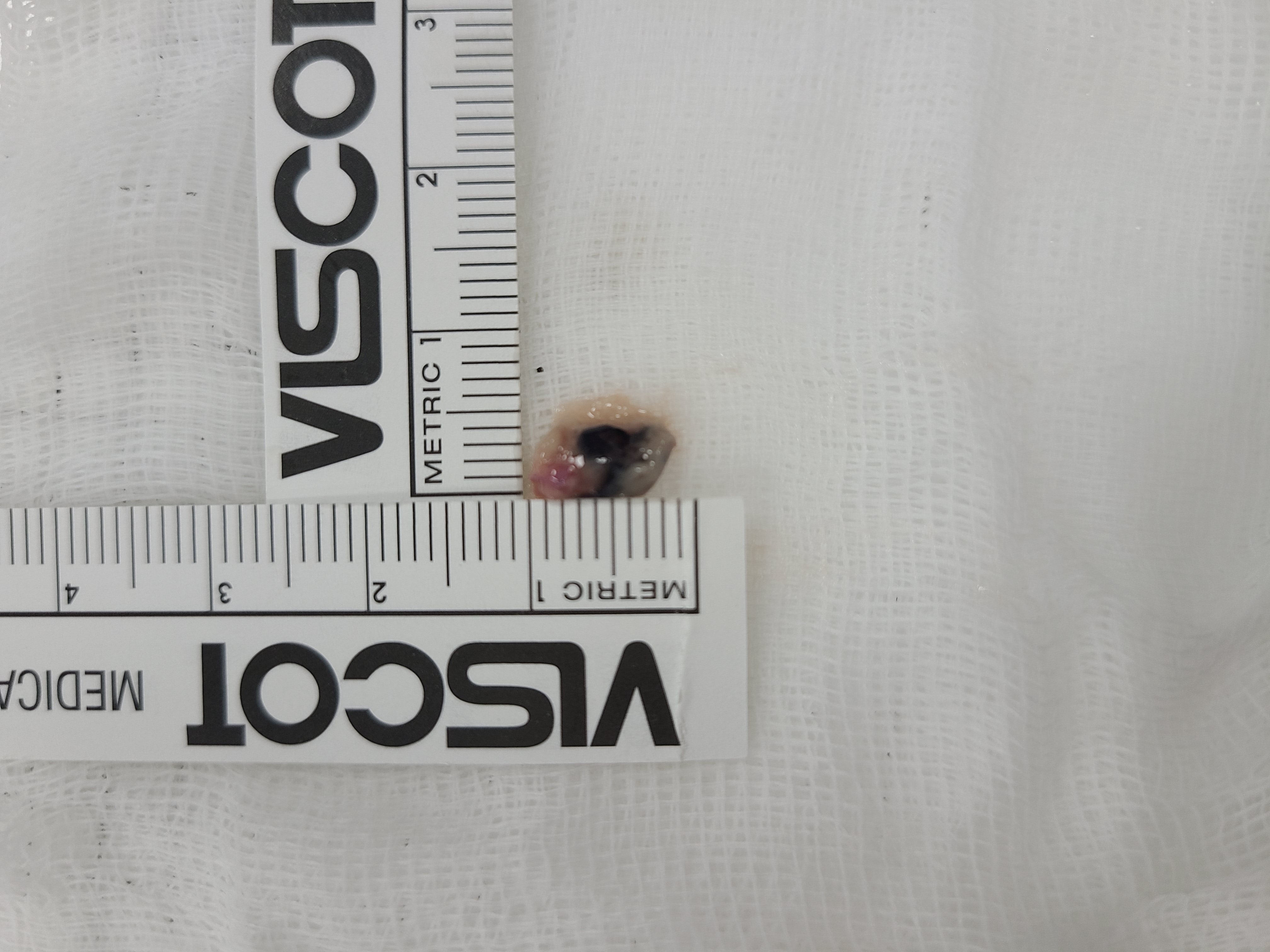

Supplement: Supplementary file 1 [file vetsci-12-01045-s001.zip › KakaoTalk_20221022_173638675_16.jpg]

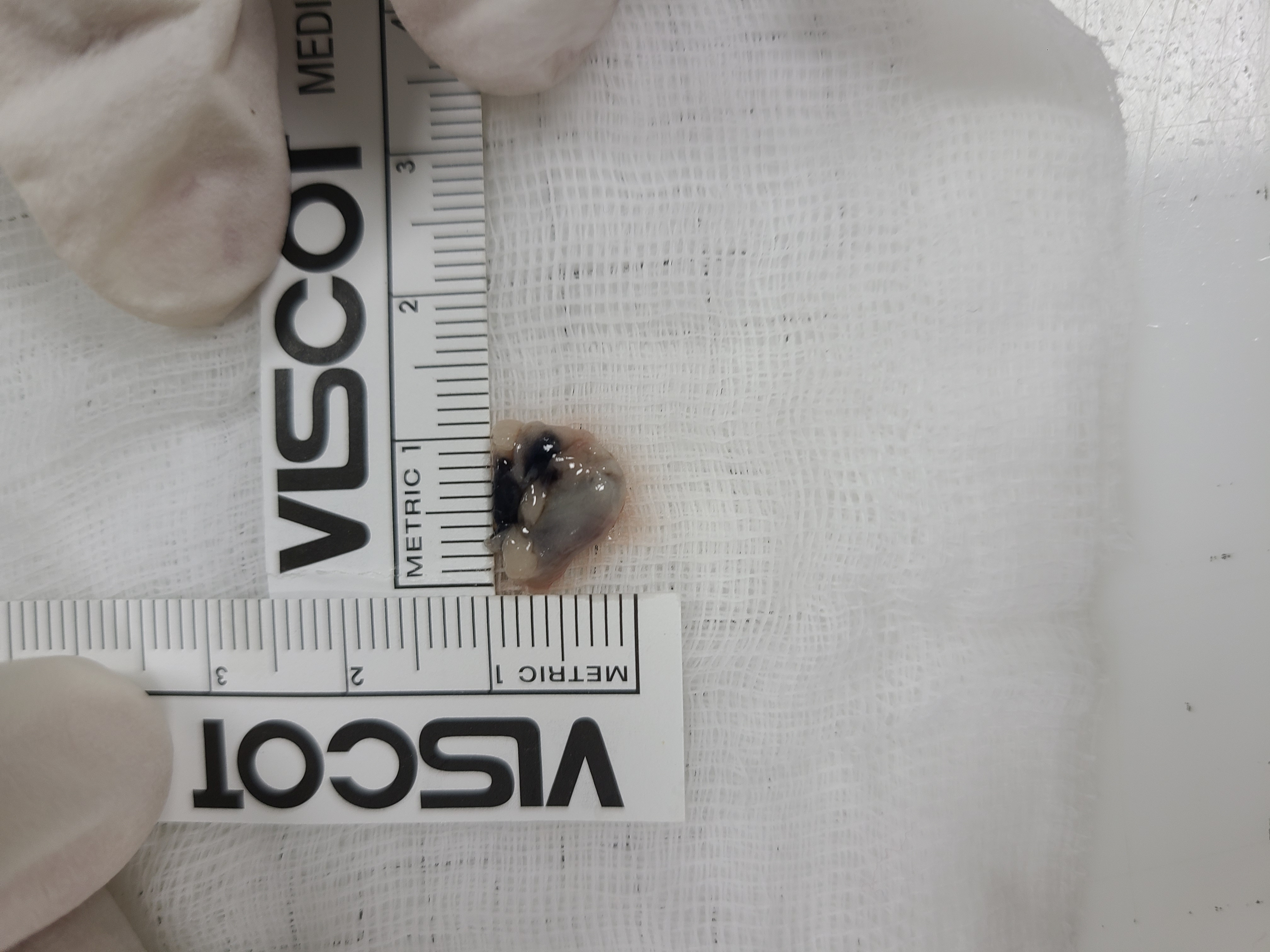

Supplement: Supplementary file 1 [file vetsci-12-01045-s001.zip › KakaoTalk_20221022_173638675_17.jpg]
