# Supplementary figures and images for: Primary Prostatic Carcinoma with Metastasis to Epaxial Muscles and Myocardium in a Dog (part 1 of 2)
Source: Vet Sci. 2025 Nov 1;12(11):1045. doi: 10.3390/vetsci12111045 (PMC12656942; doi:10.3390/vetsci12111045)

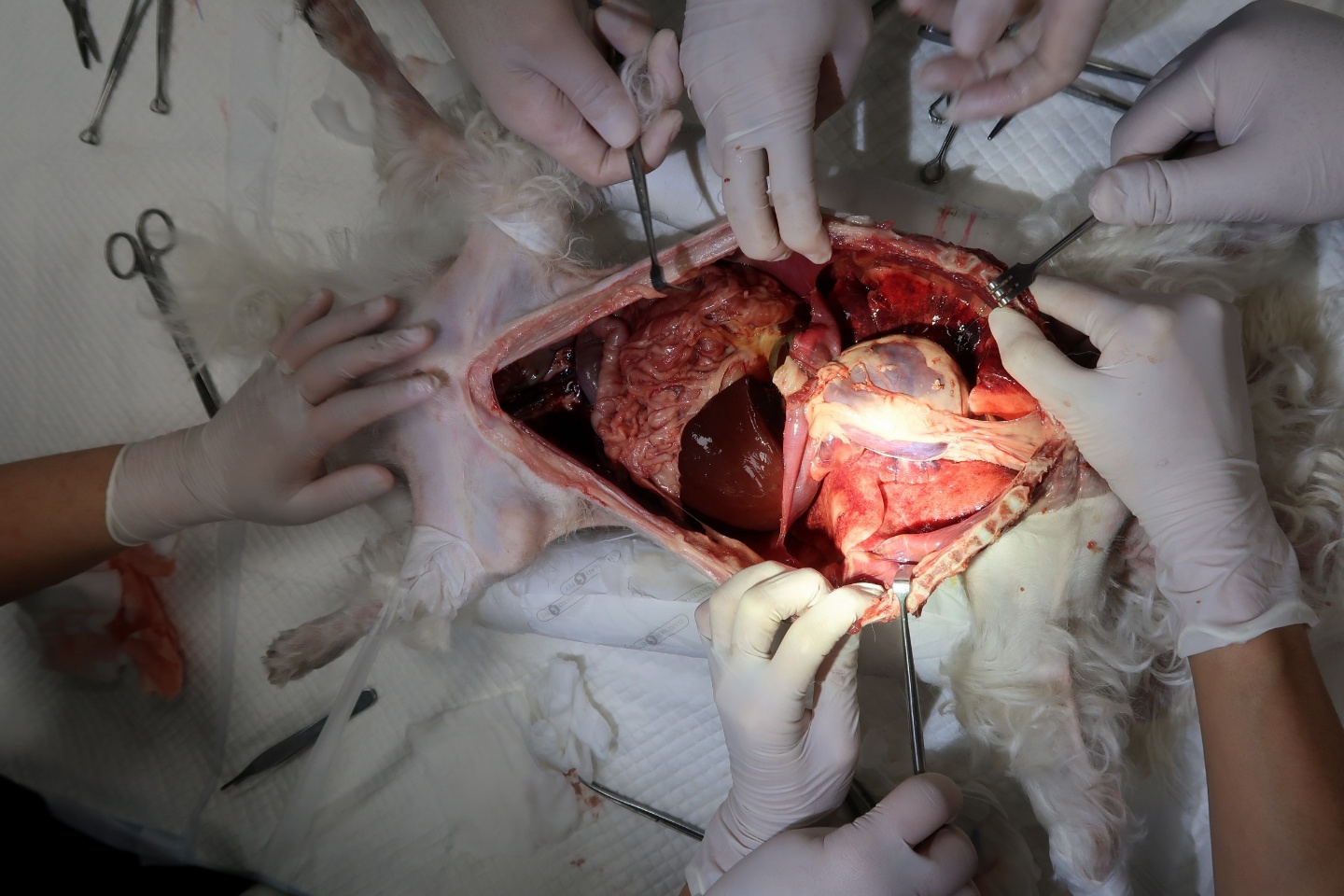

Supplement: Supplementary file 1 [file vetsci-12-01045-s001.zip › KakaoTalk_20221022_163449407.jpg]

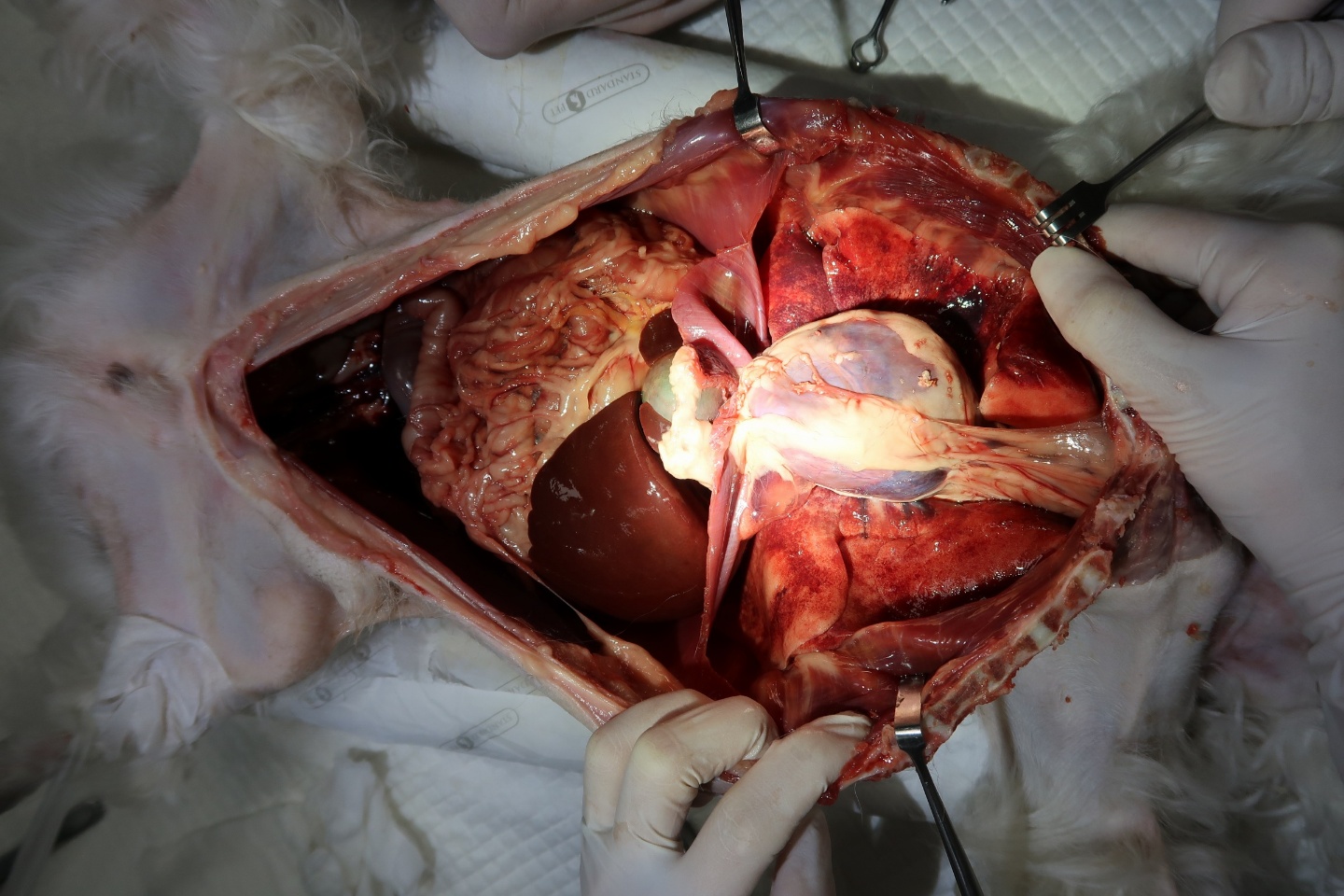

Supplement: Supplementary file 1 [file vetsci-12-01045-s001.zip › KakaoTalk_20221022_163449407_01.jpg]

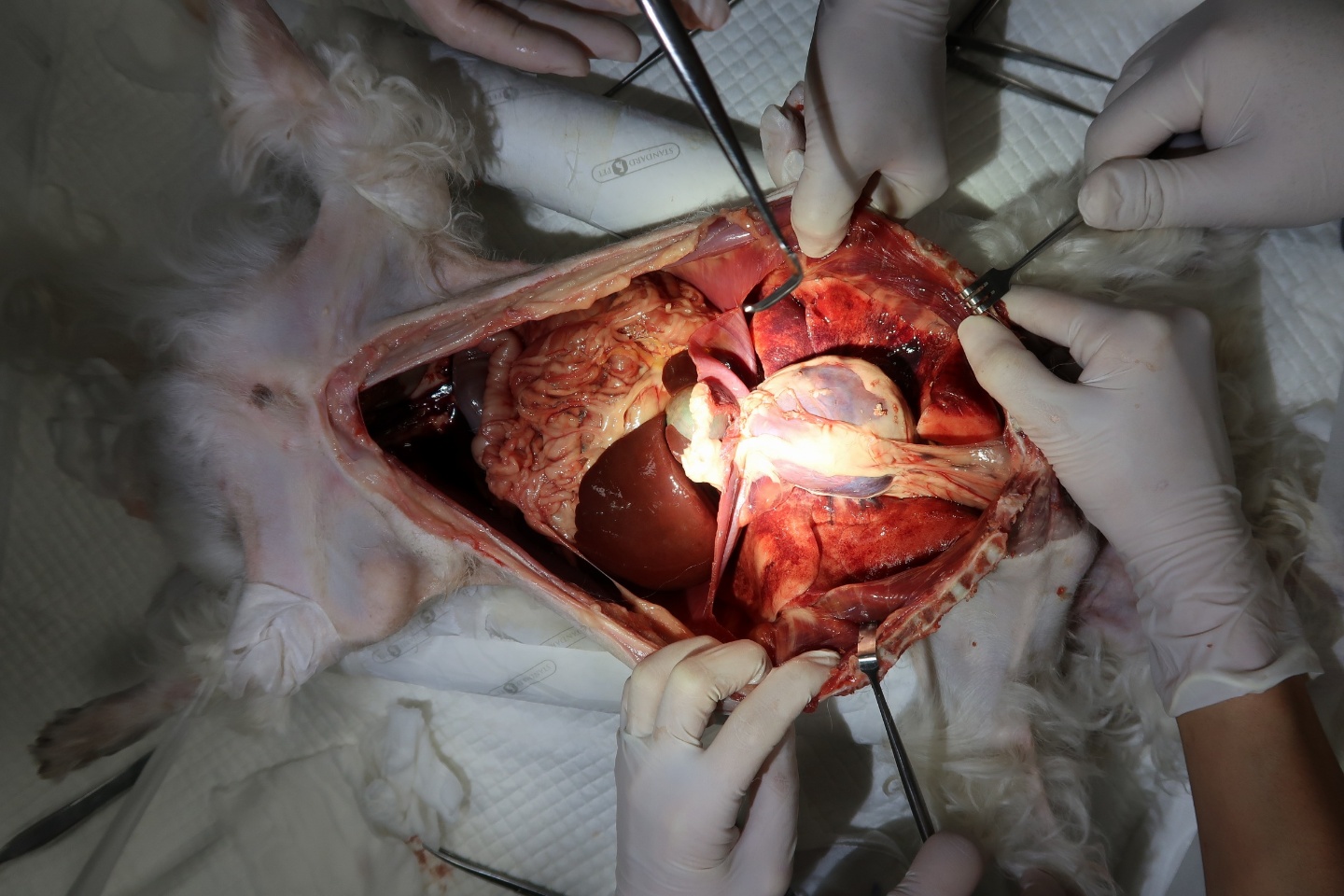

Supplement: Supplementary file 1 [file vetsci-12-01045-s001.zip › KakaoTalk_20221022_163449407_02.jpg]

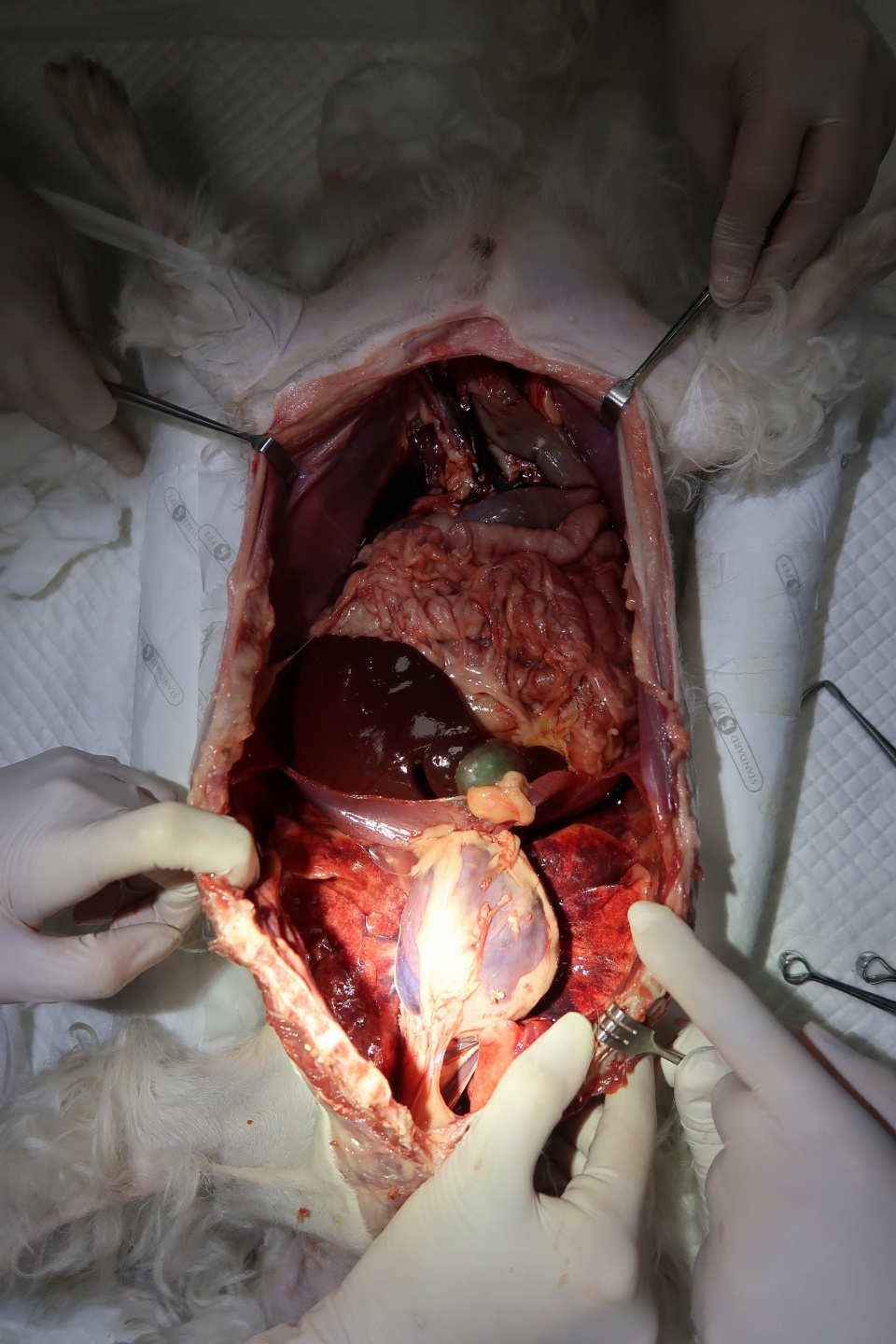

Supplement: Supplementary file 1 [file vetsci-12-01045-s001.zip › KakaoTalk_20221022_163449407_03.jpg]

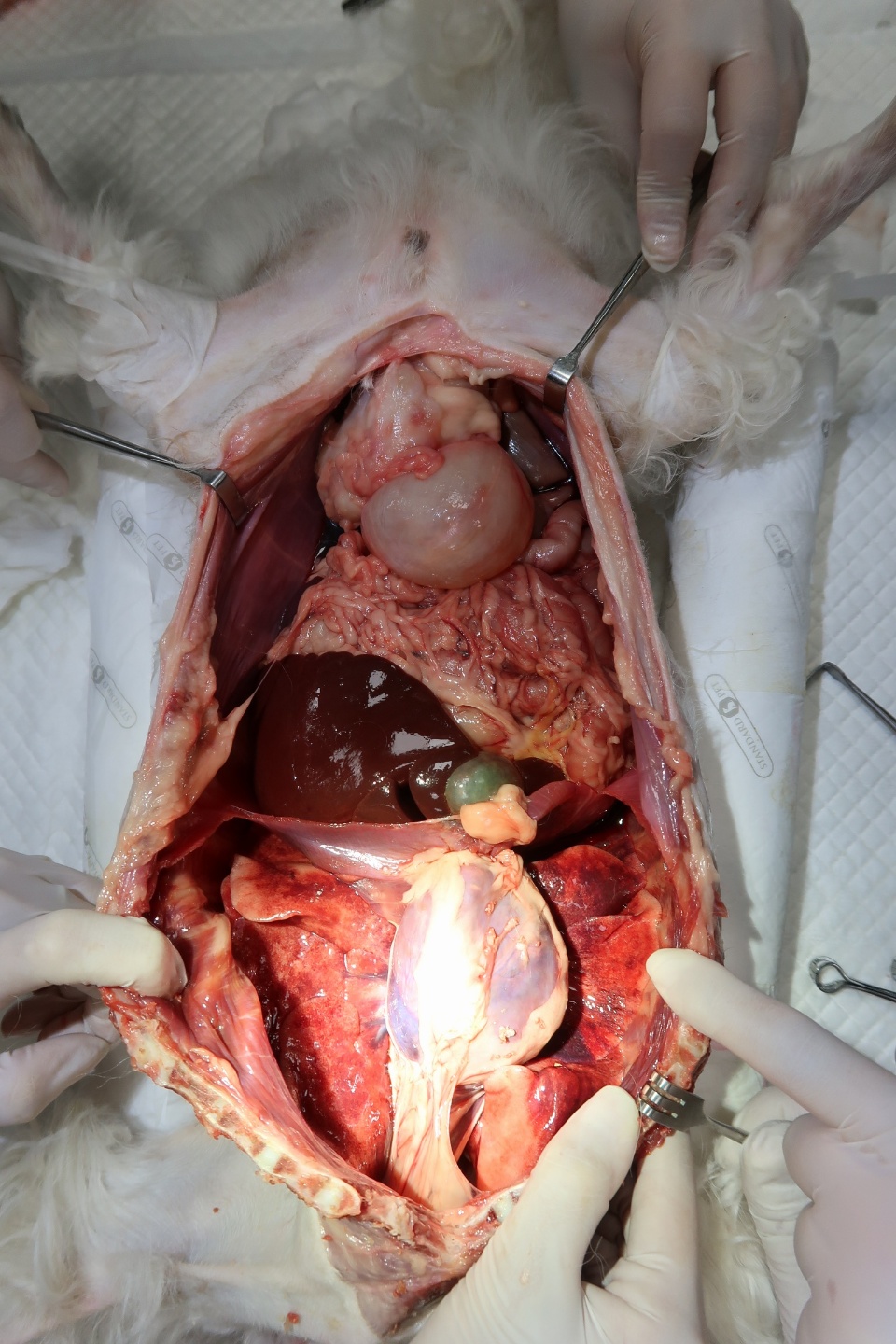

Supplement: Supplementary file 1 [file vetsci-12-01045-s001.zip › KakaoTalk_20221022_163449407_04.jpg]

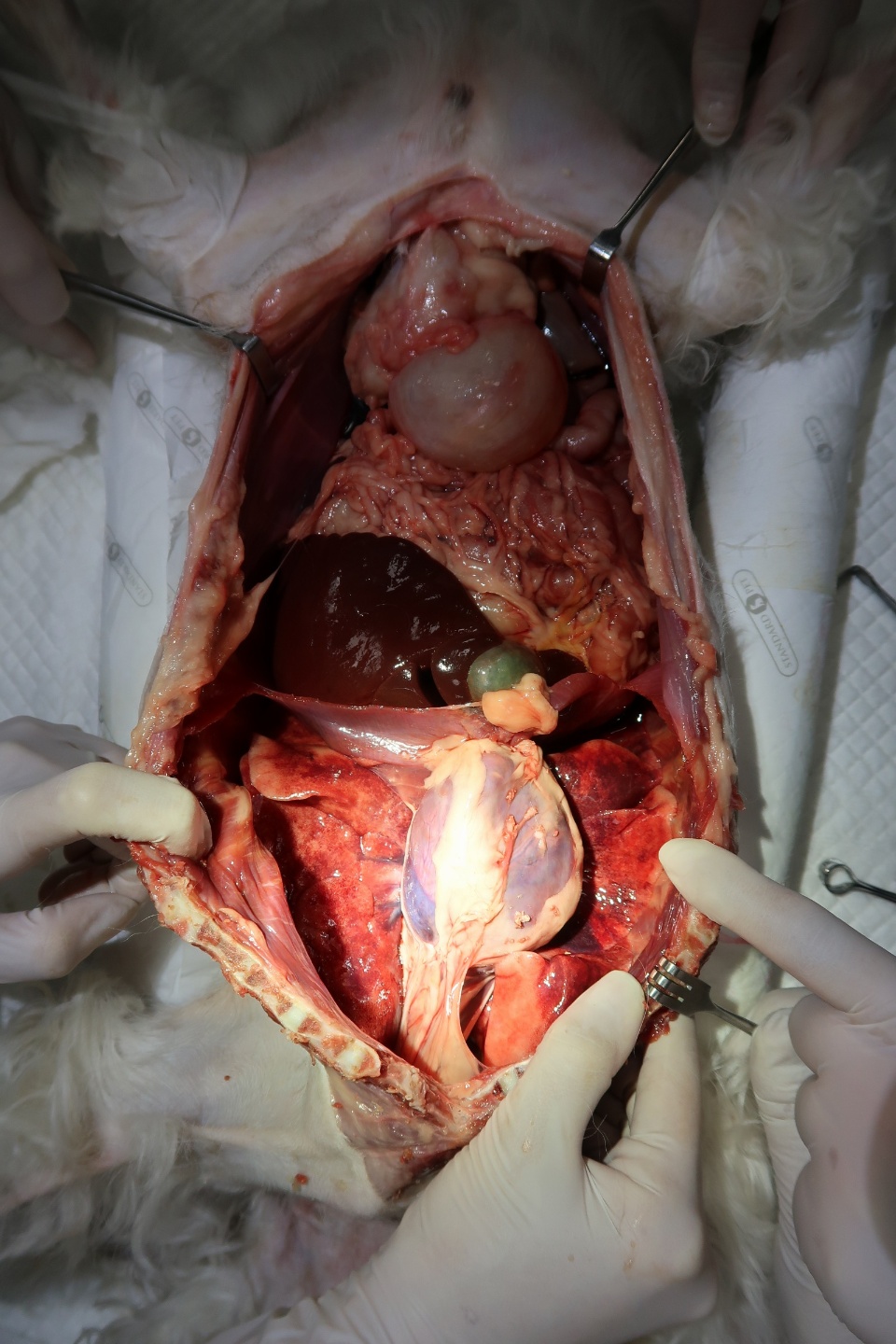

Supplement: Supplementary file 1 [file vetsci-12-01045-s001.zip › KakaoTalk_20221022_163449407_05.jpg]

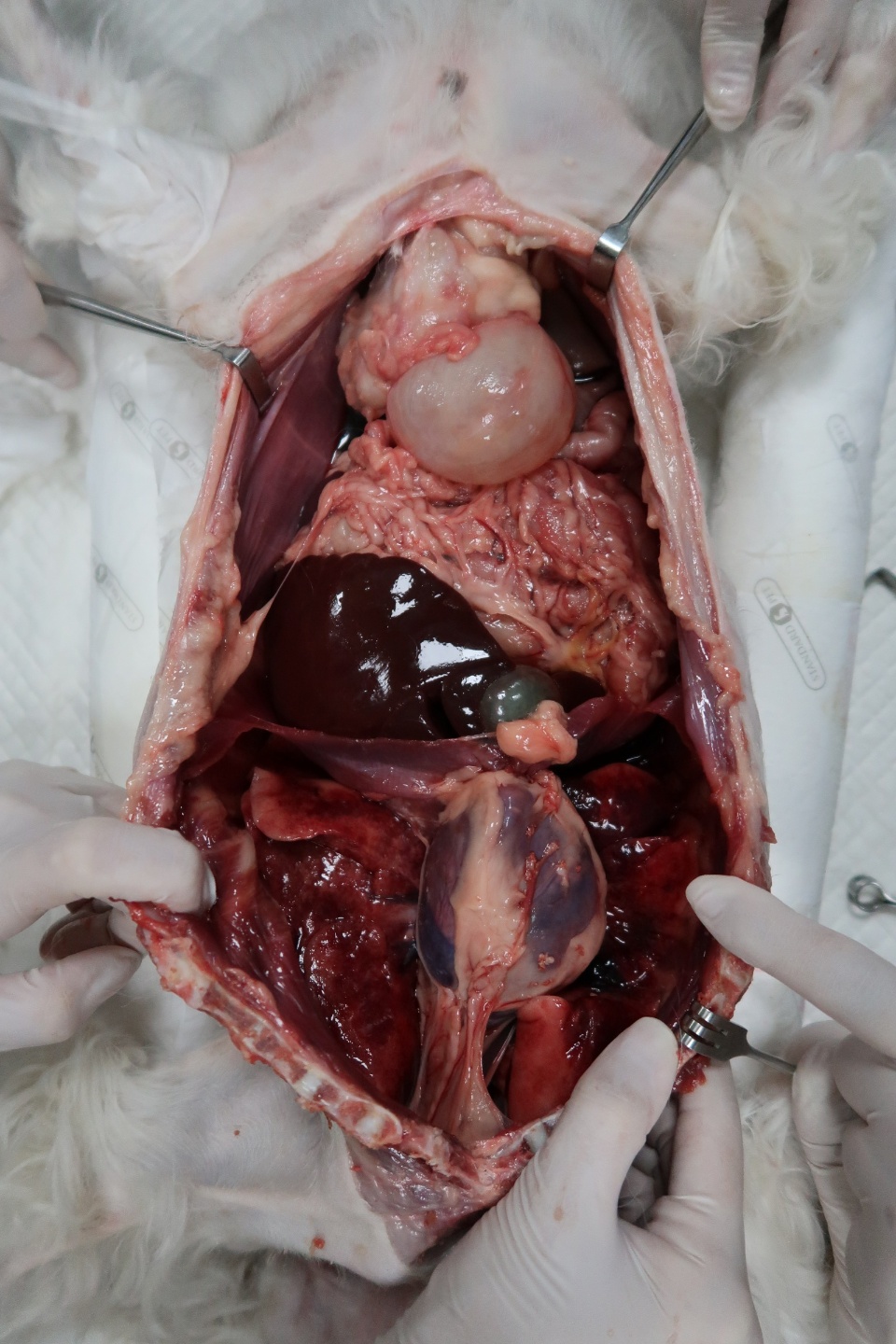

Supplement: Supplementary file 1 [file vetsci-12-01045-s001.zip › KakaoTalk_20221022_163449407_06.jpg]

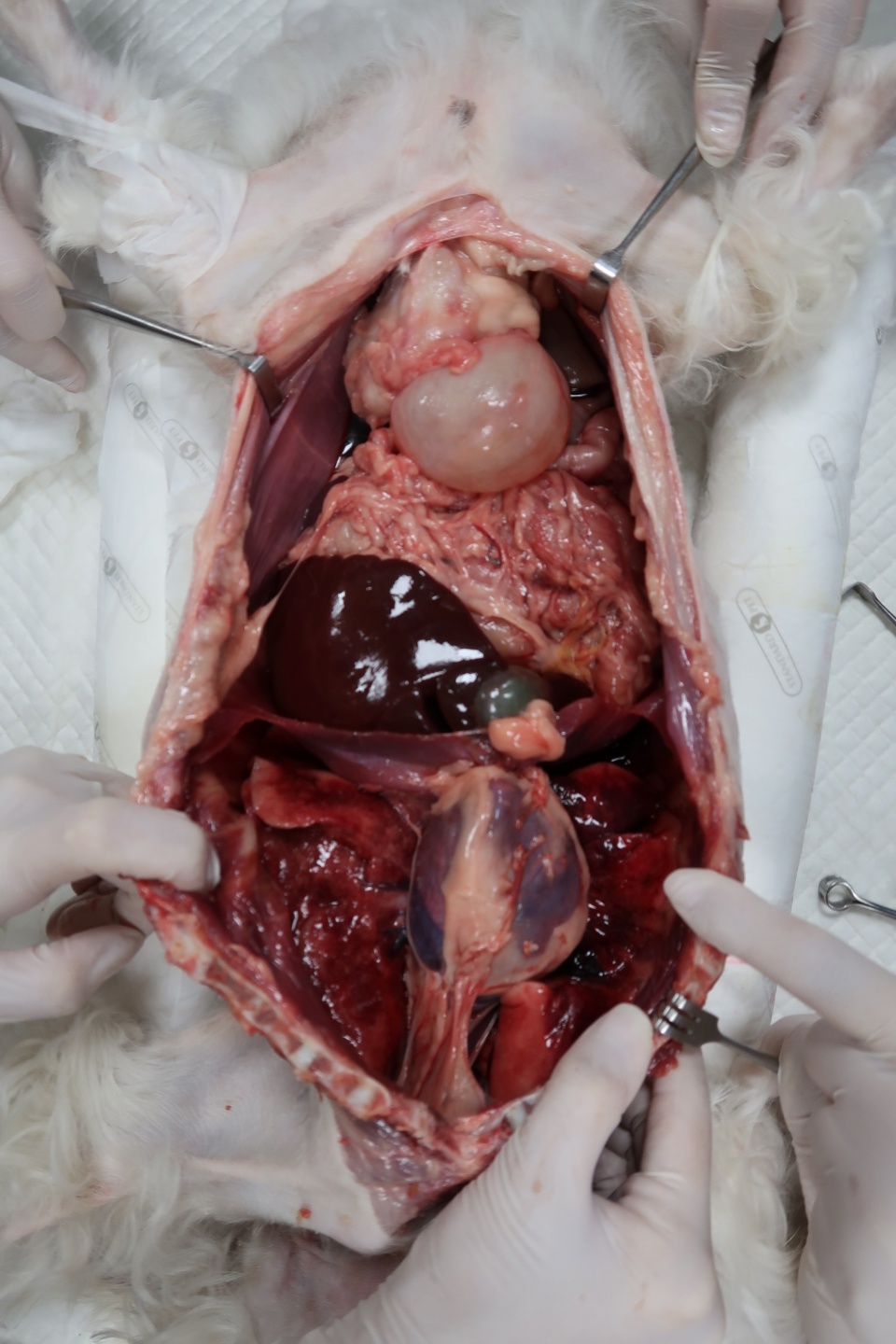

Supplement: Supplementary file 1 [file vetsci-12-01045-s001.zip › KakaoTalk_20221022_163449407_07.jpg]

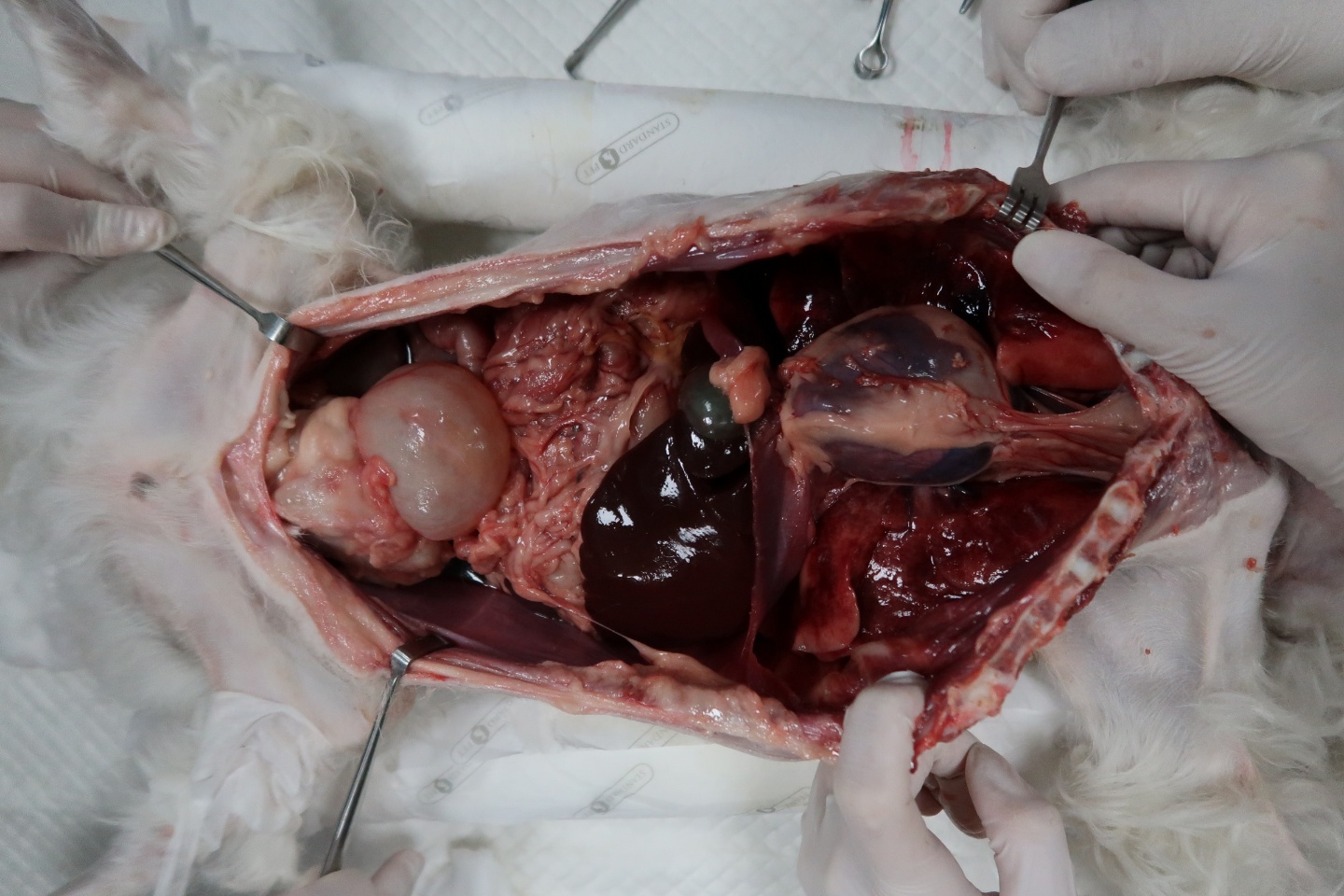

Supplement: Supplementary file 1 [file vetsci-12-01045-s001.zip › KakaoTalk_20221022_163449407_08.jpg]

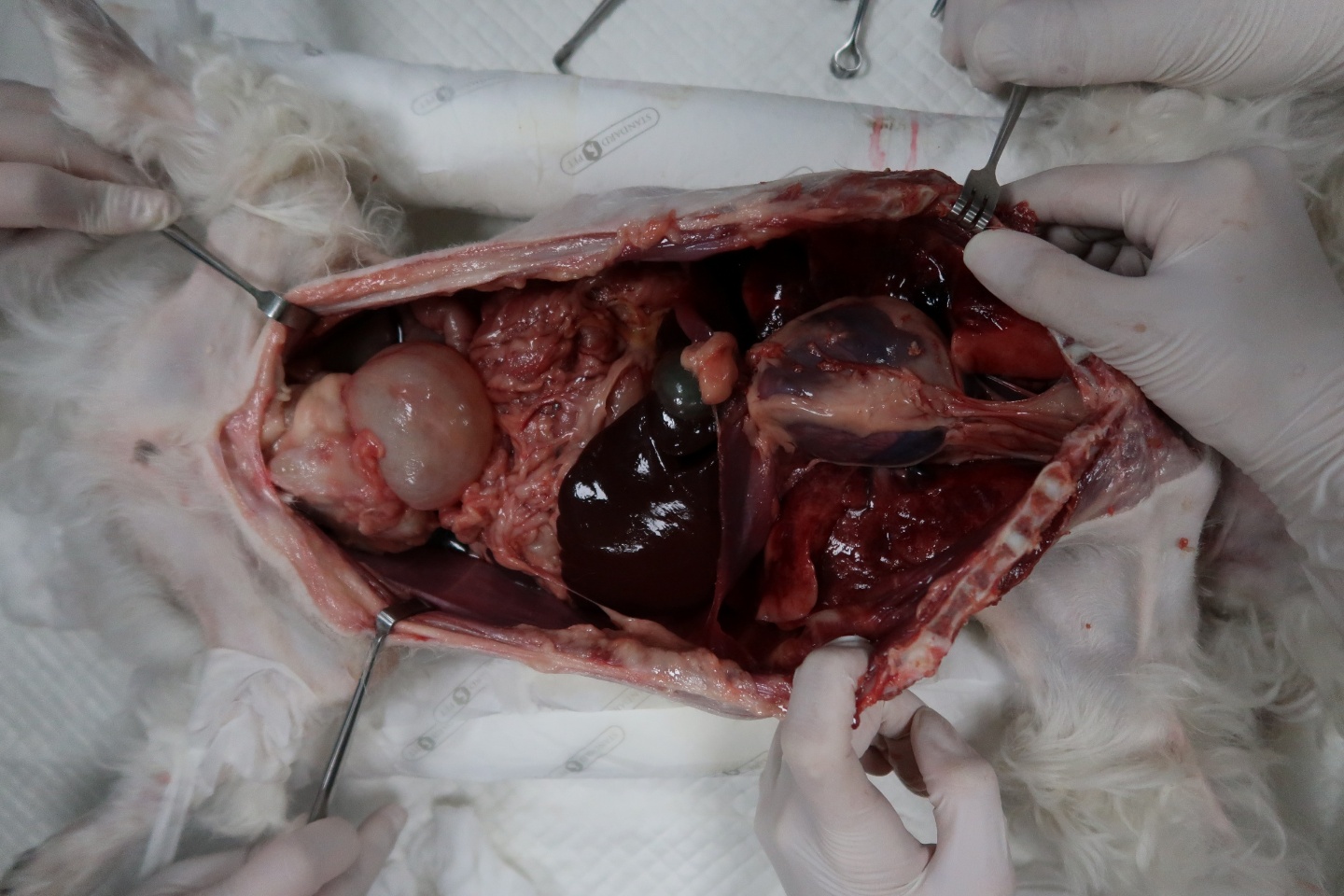

Supplement: Supplementary file 1 [file vetsci-12-01045-s001.zip › KakaoTalk_20221022_163449407_09.jpg]

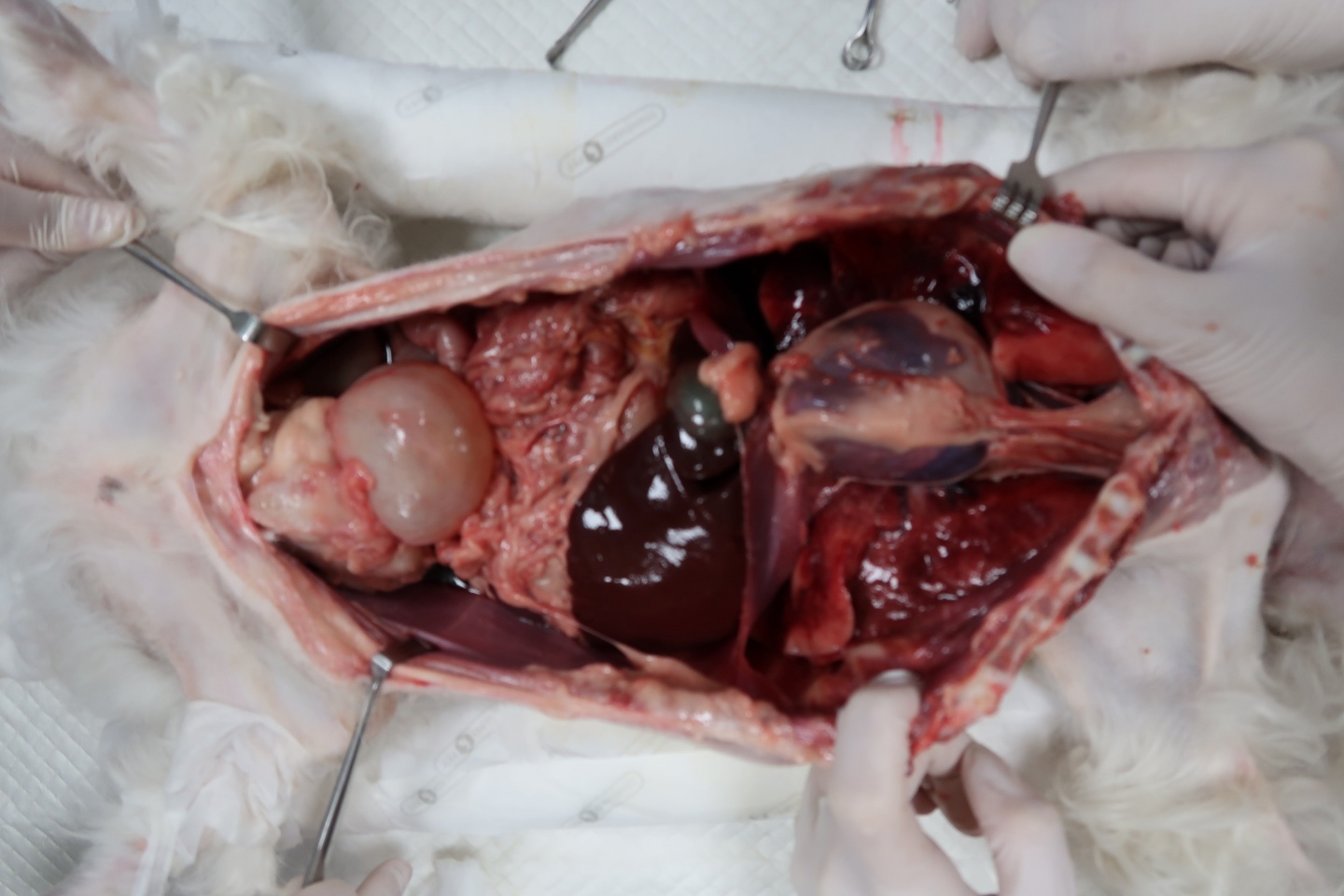

Supplement: Supplementary file 1 [file vetsci-12-01045-s001.zip › KakaoTalk_20221022_163449407_10.jpg]

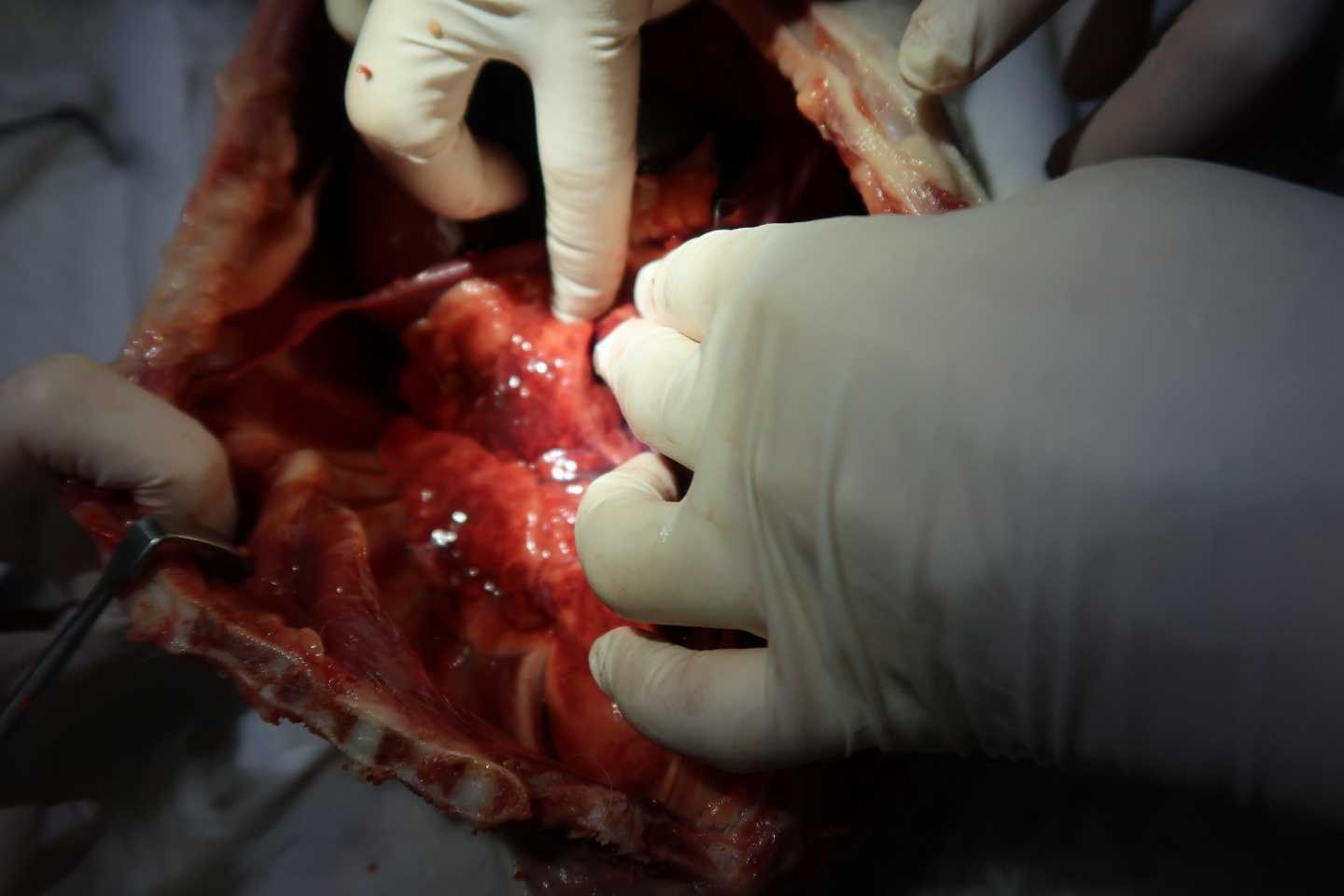

Supplement: Supplementary file 1 [file vetsci-12-01045-s001.zip › KakaoTalk_20221022_163449407_11.jpg]

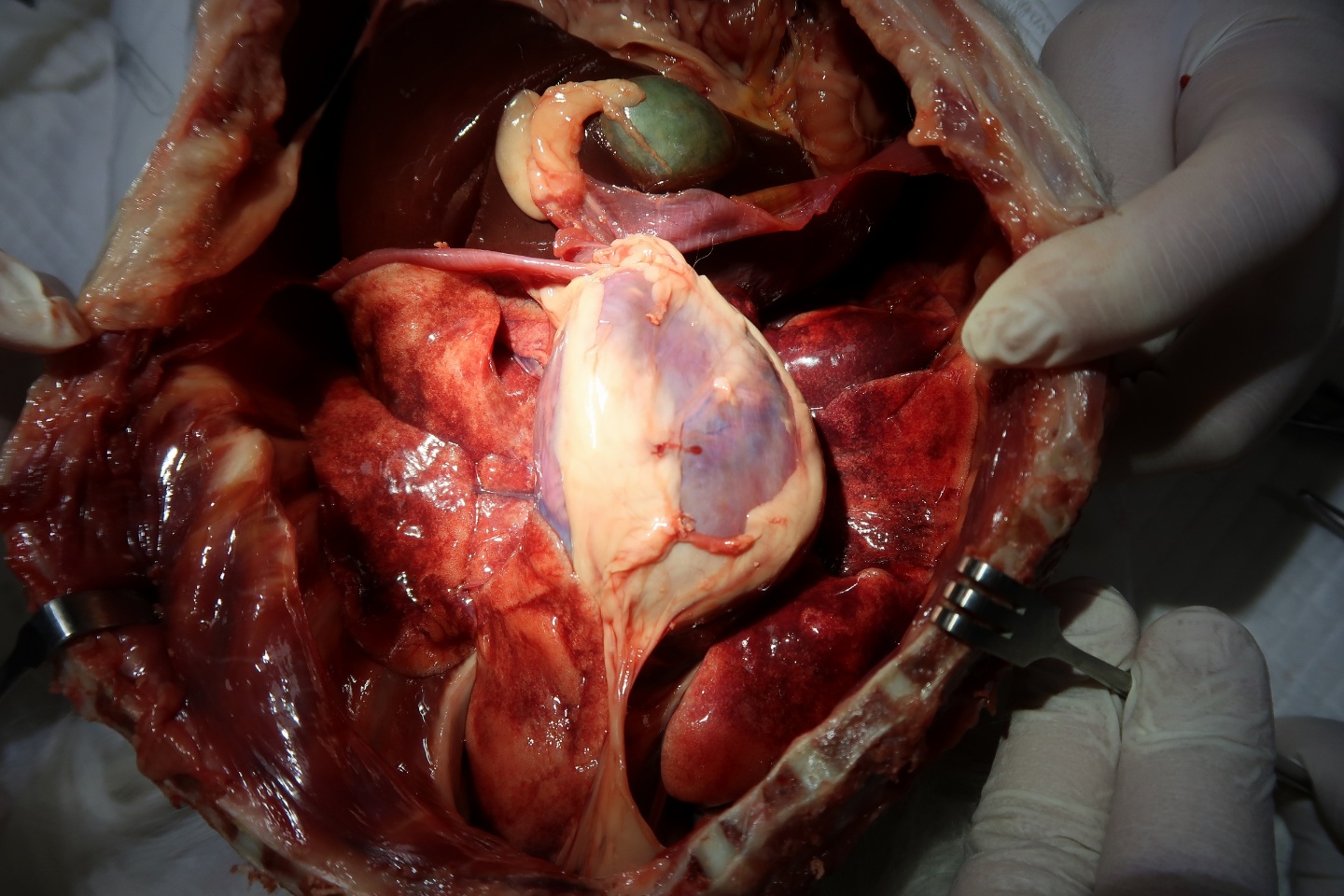

Supplement: Supplementary file 1 [file vetsci-12-01045-s001.zip › KakaoTalk_20221022_163449407_12.jpg]

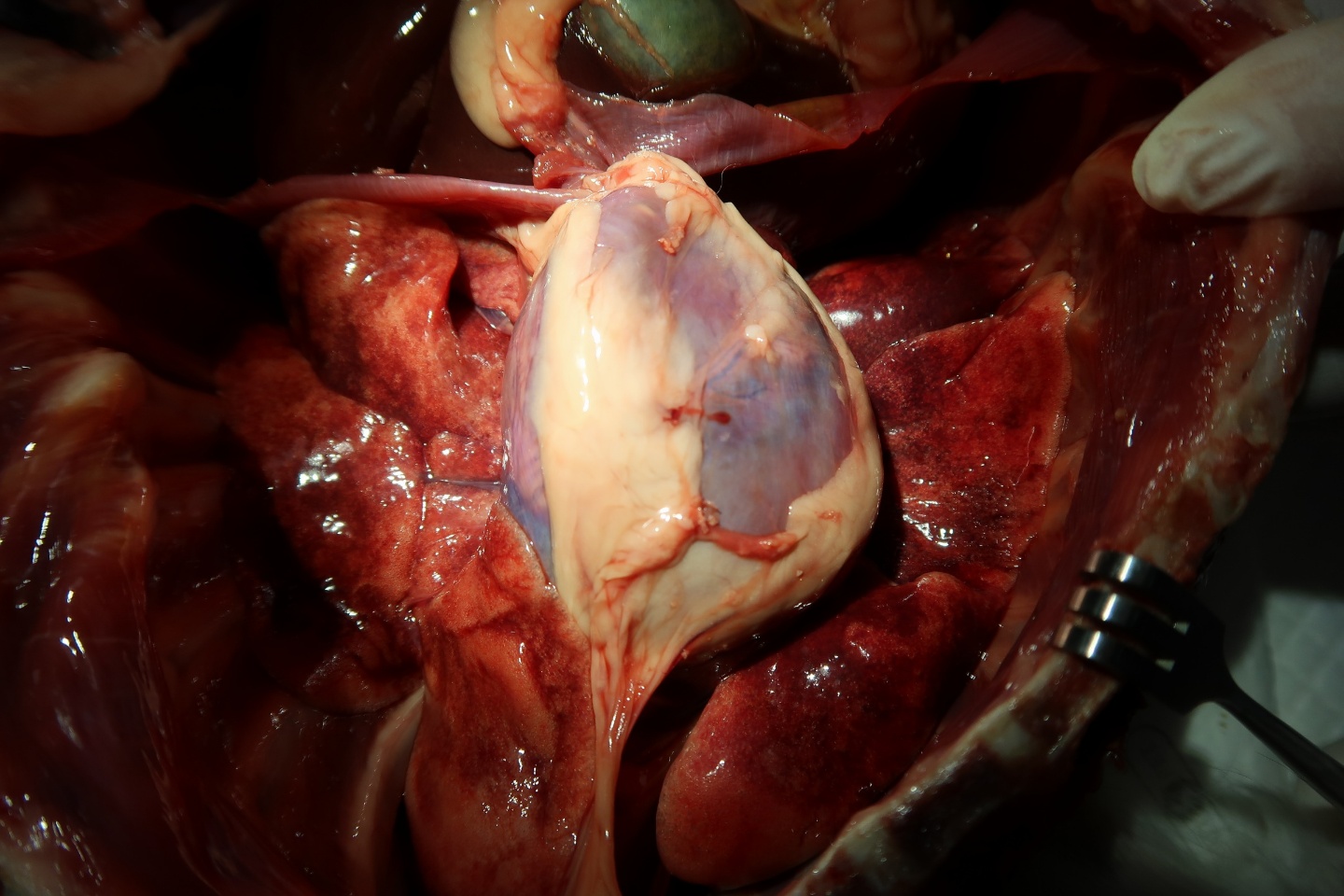

Supplement: Supplementary file 1 [file vetsci-12-01045-s001.zip › KakaoTalk_20221022_163449407_13.jpg]

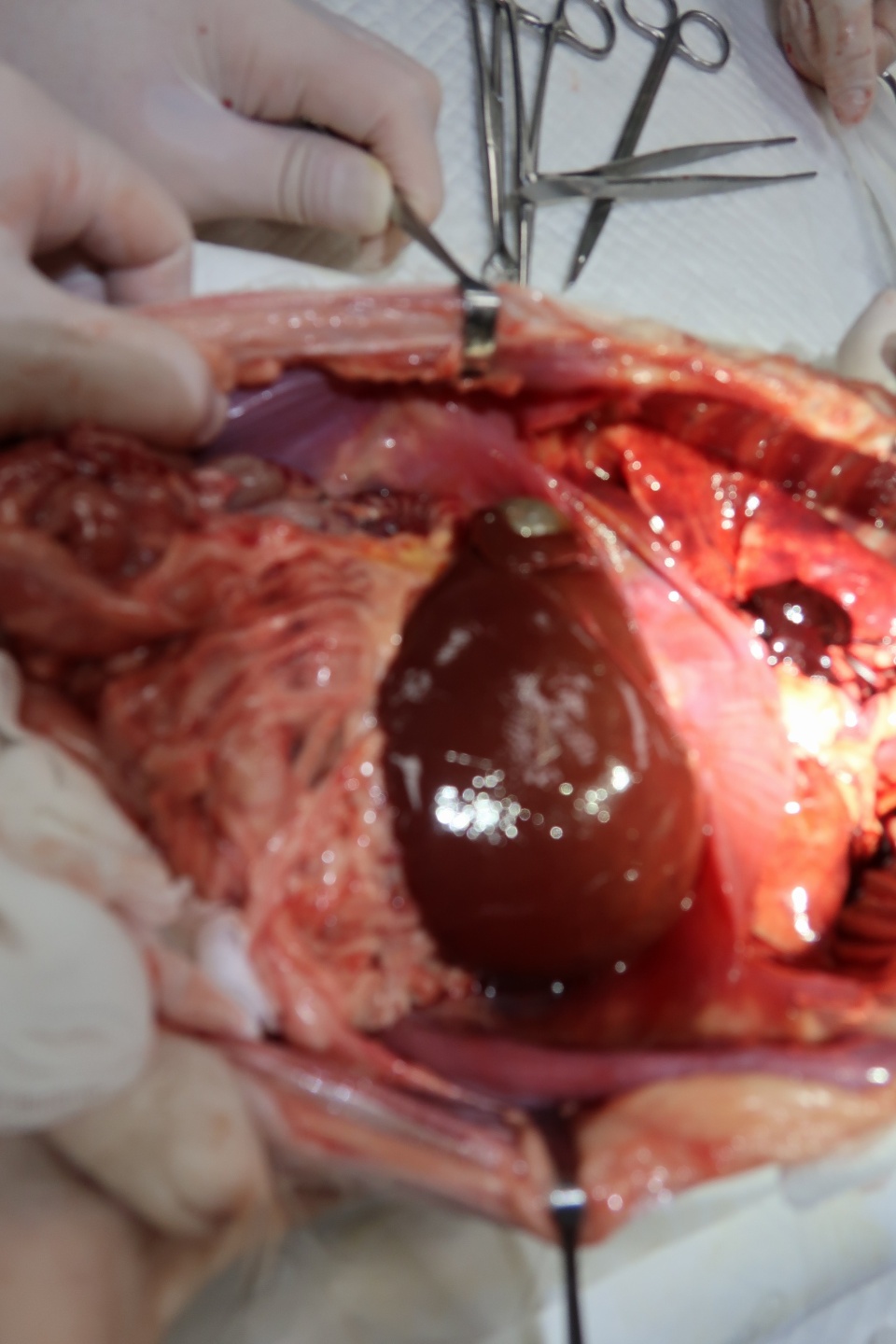

Supplement: Supplementary file 1 [file vetsci-12-01045-s001.zip › KakaoTalk_20221022_163449407_14.jpg]

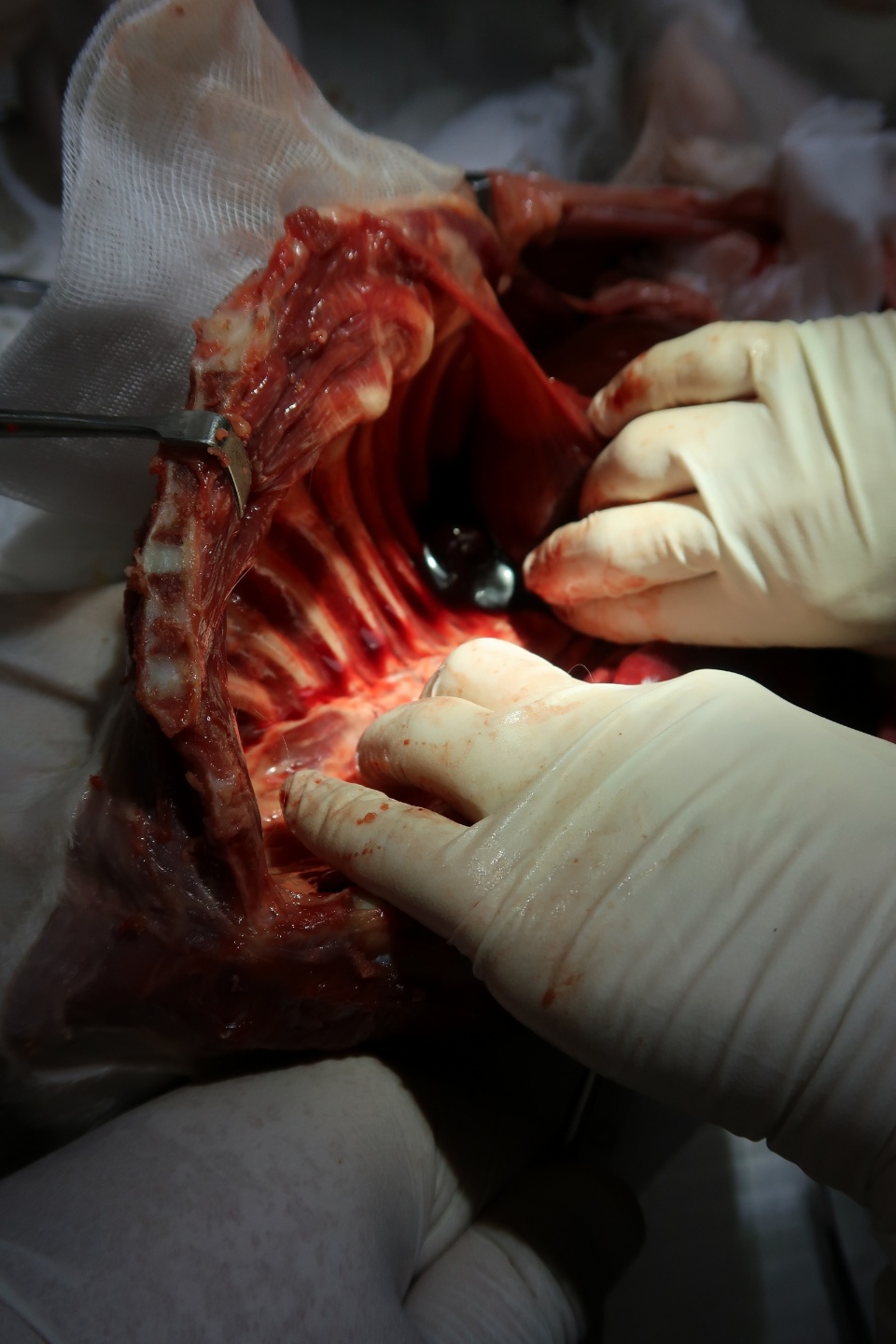

Supplement: Supplementary file 1 [file vetsci-12-01045-s001.zip › KakaoTalk_20221022_163449407_15.jpg]

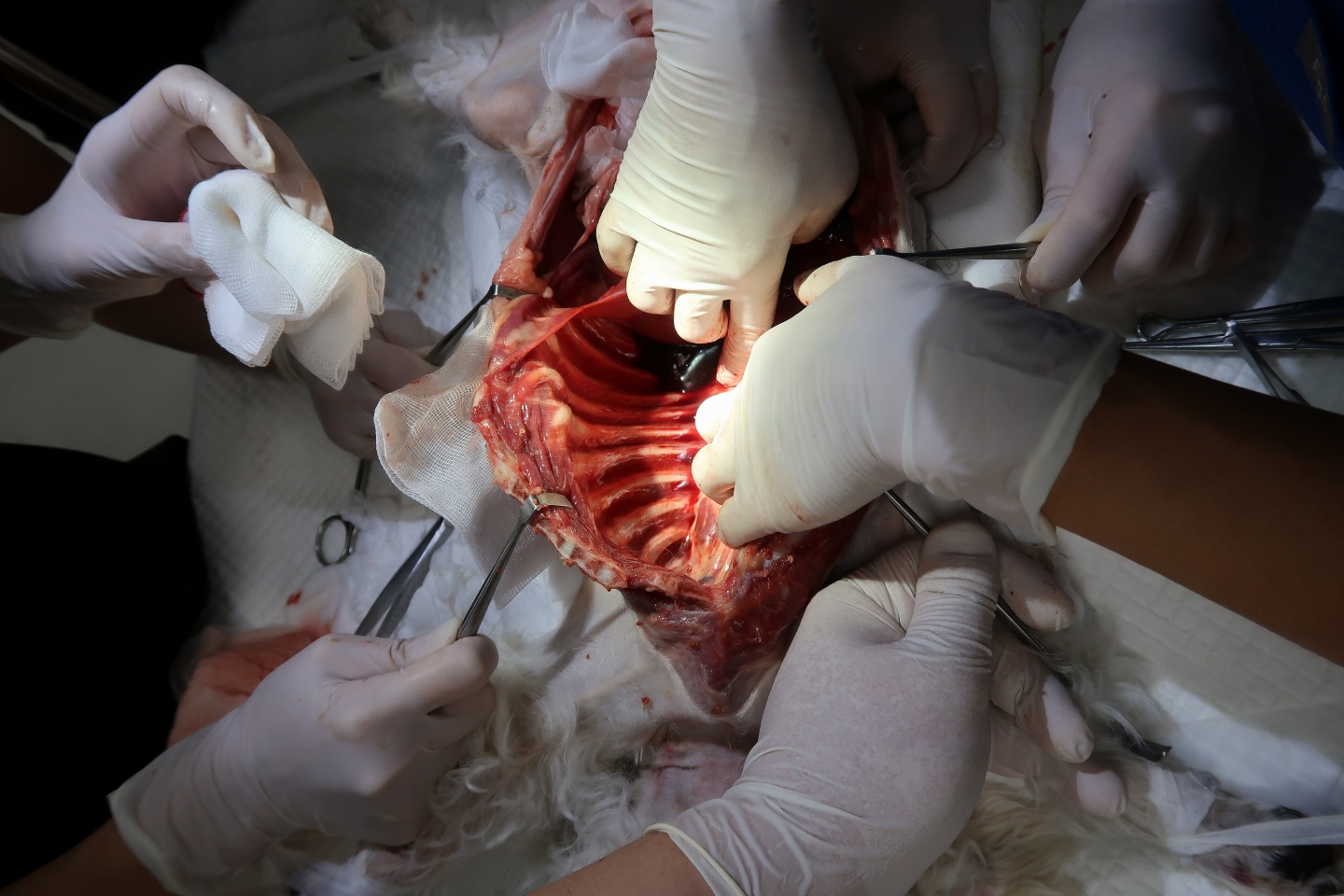

Supplement: Supplementary file 1 [file vetsci-12-01045-s001.zip › KakaoTalk_20221022_163449407_16.jpg]

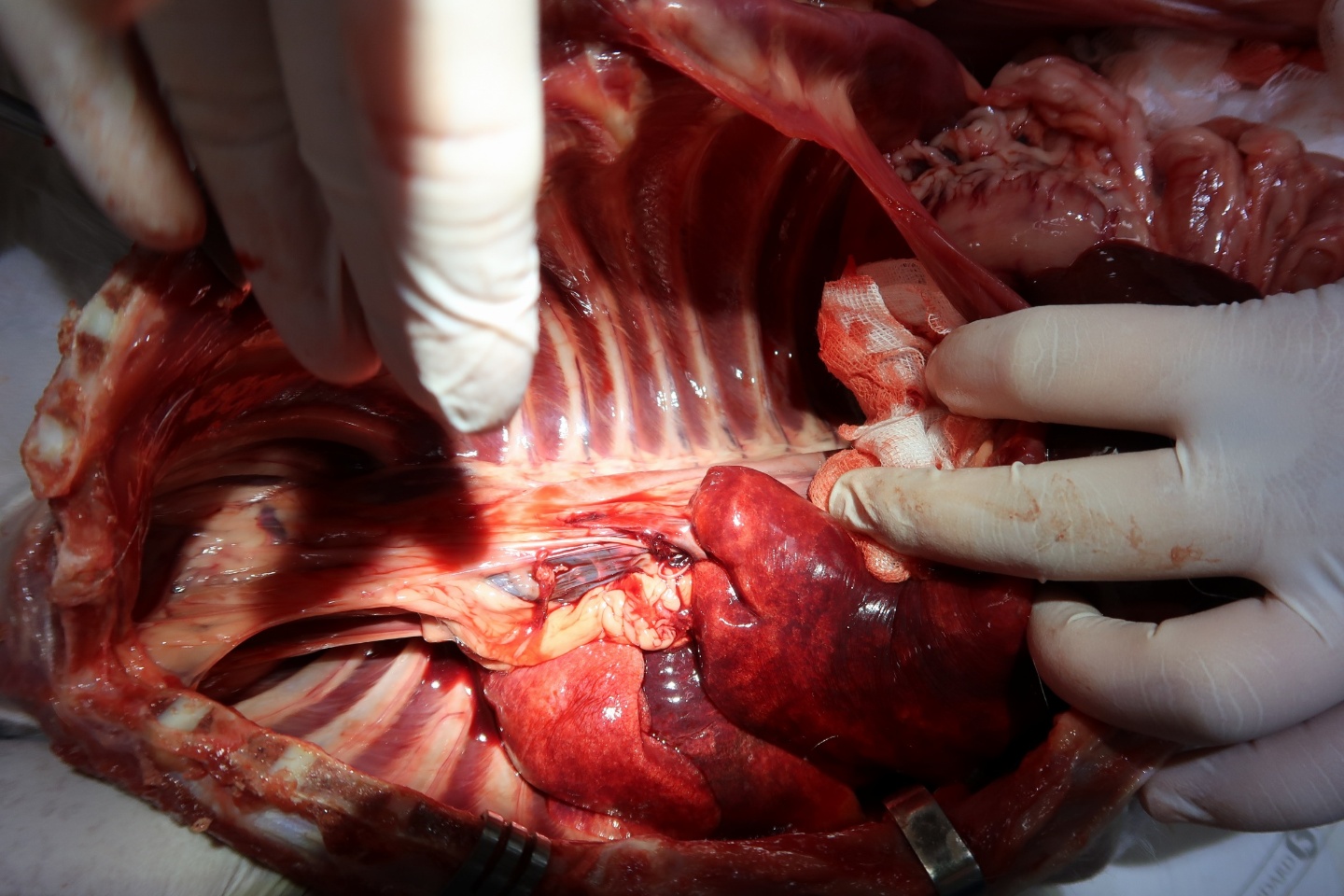

Supplement: Supplementary file 1 [file vetsci-12-01045-s001.zip › KakaoTalk_20221022_163449407_17.jpg]

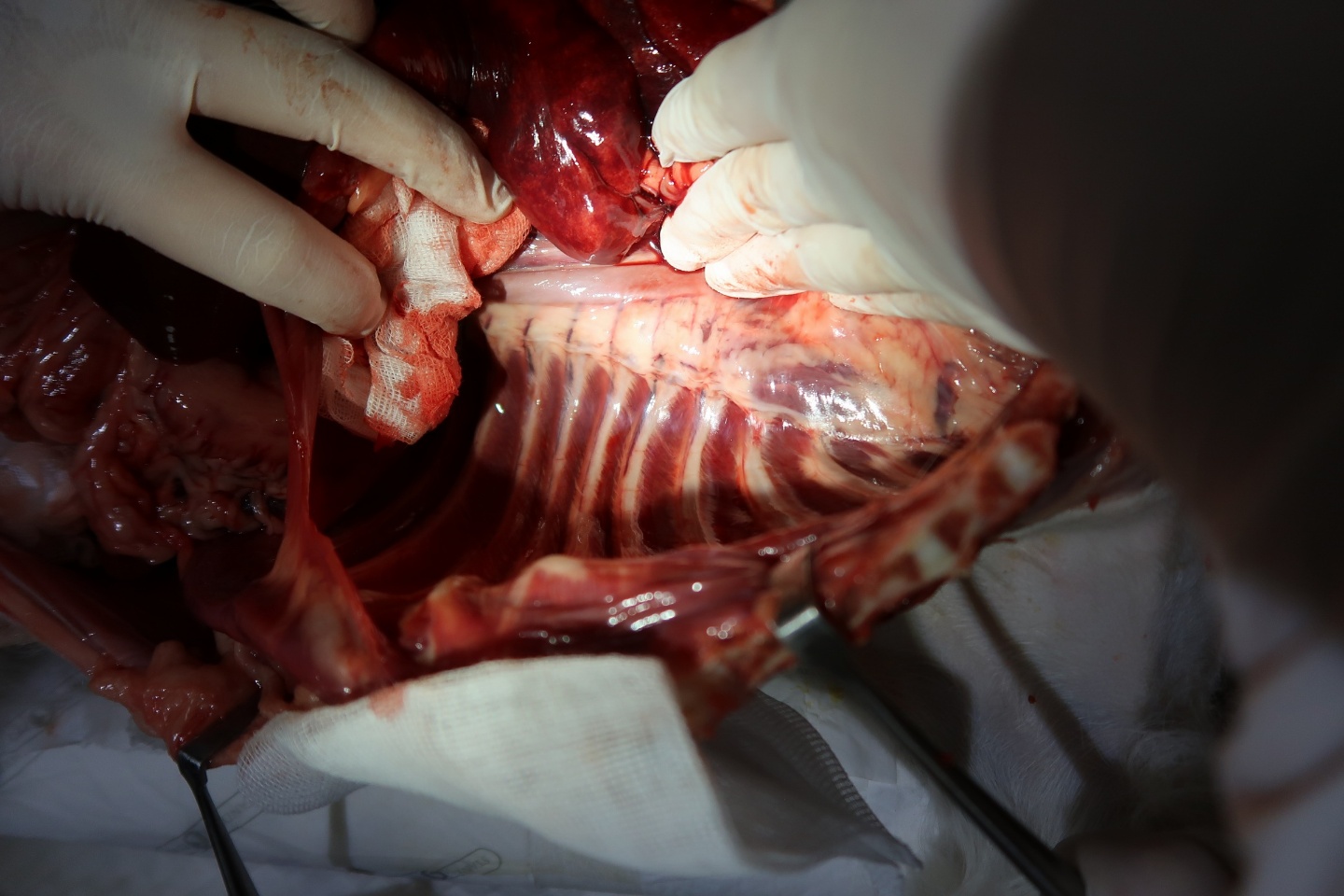

Supplement: Supplementary file 1 [file vetsci-12-01045-s001.zip › KakaoTalk_20221022_163449407_18.jpg]

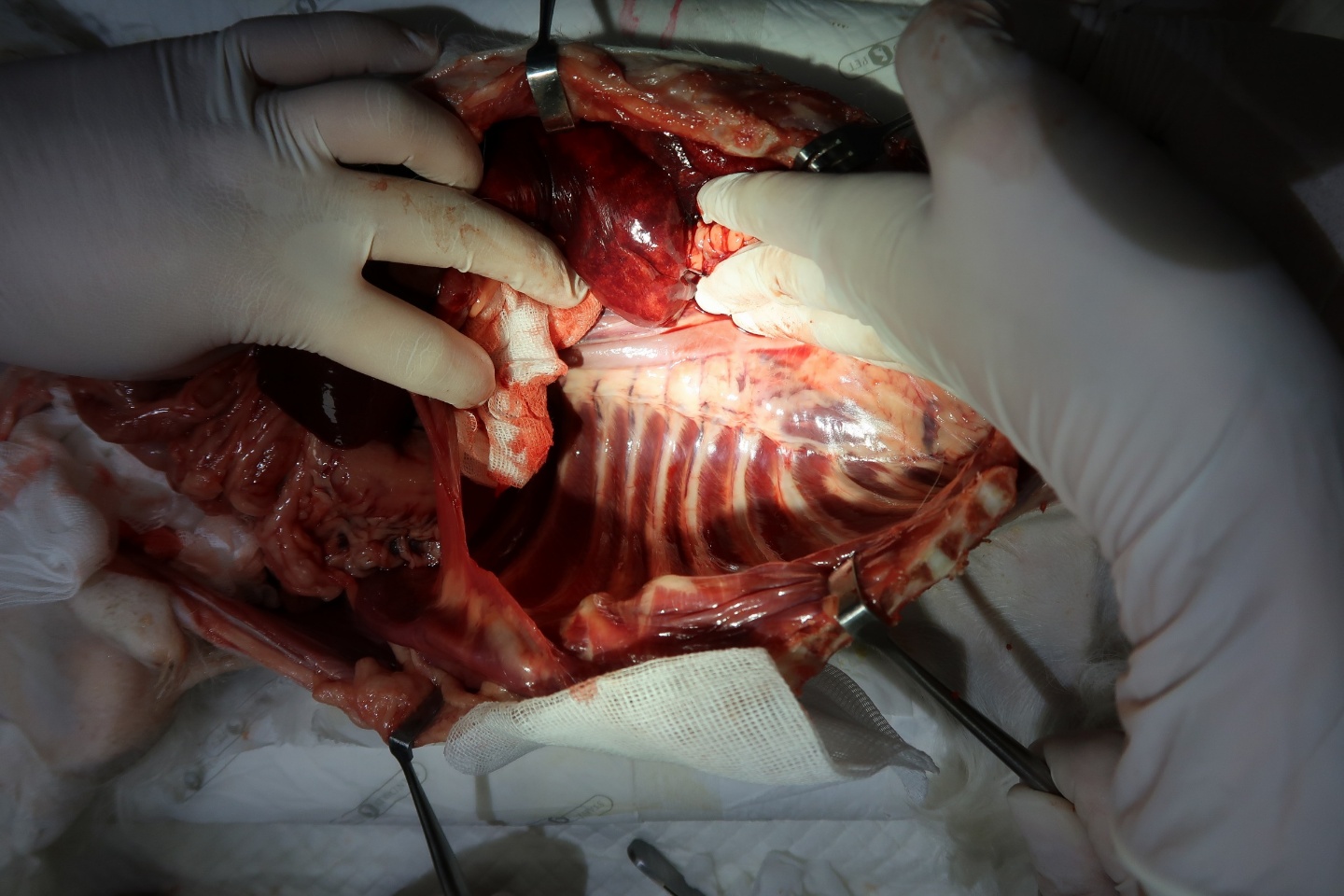

Supplement: Supplementary file 1 [file vetsci-12-01045-s001.zip › KakaoTalk_20221022_163449407_19.jpg]

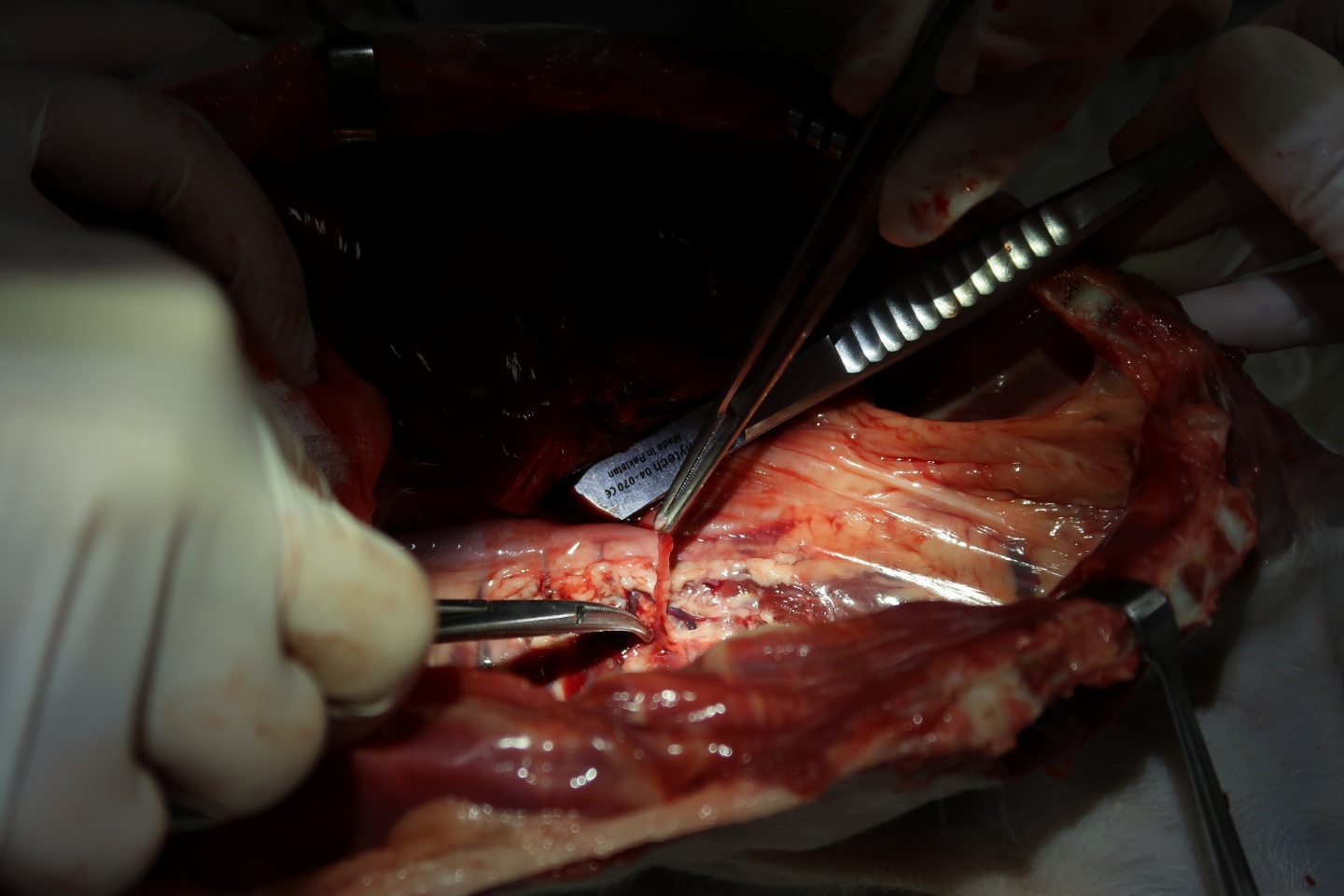

Supplement: Supplementary file 1 [file vetsci-12-01045-s001.zip › KakaoTalk_20221022_163449407_20.jpg]

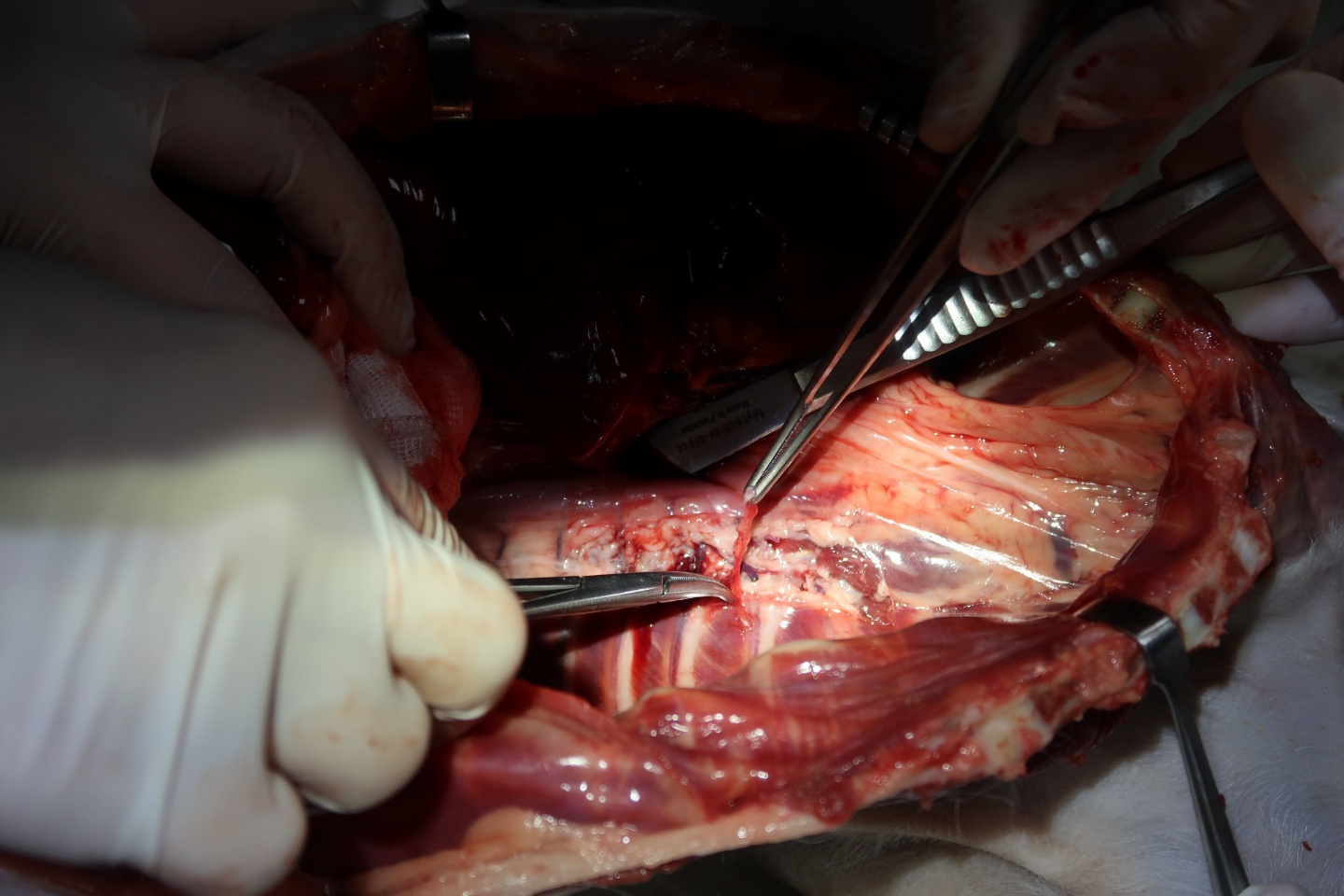

Supplement: Supplementary file 1 [file vetsci-12-01045-s001.zip › KakaoTalk_20221022_163449407_21.jpg]

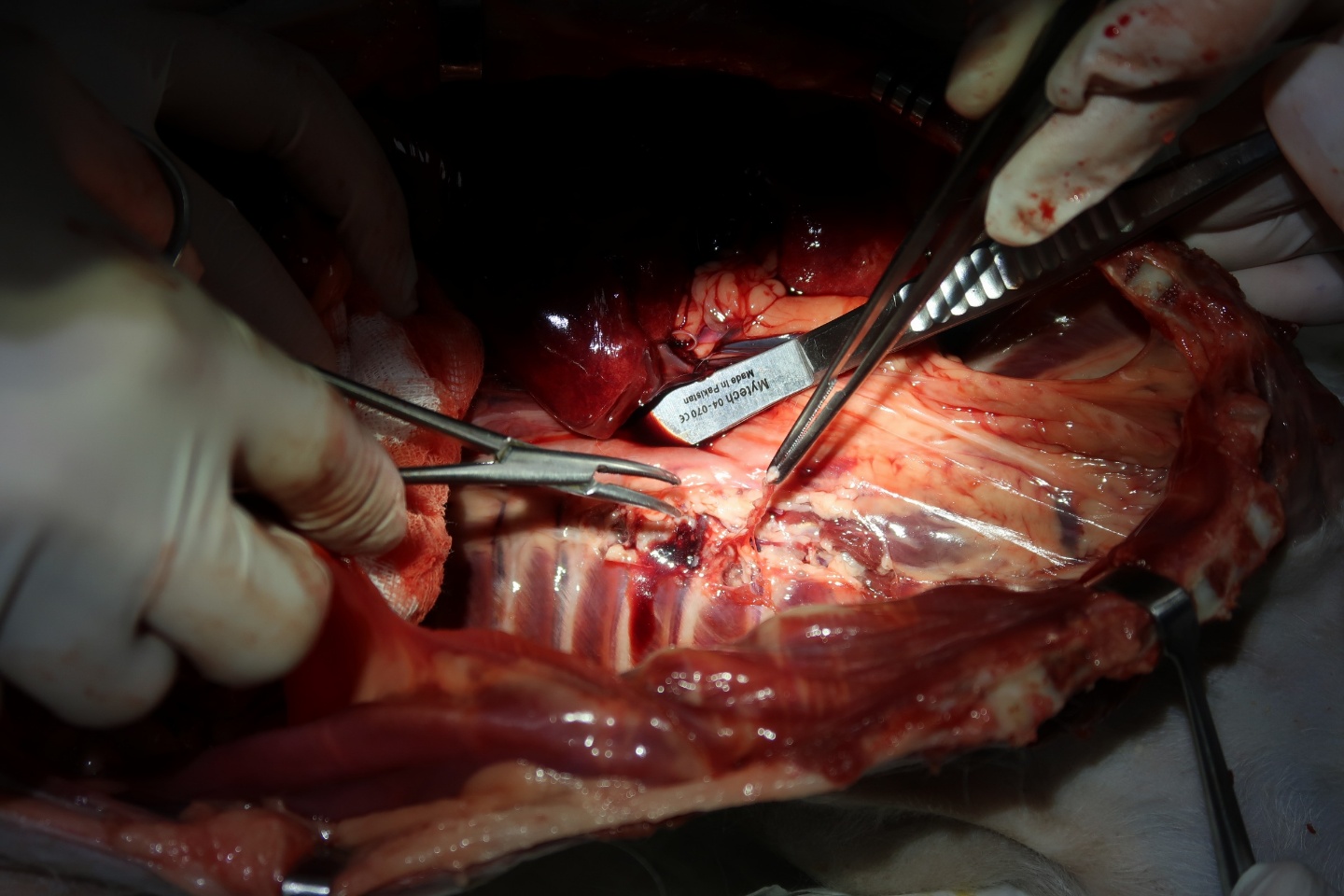

Supplement: Supplementary file 1 [file vetsci-12-01045-s001.zip › KakaoTalk_20221022_163449407_22.jpg]

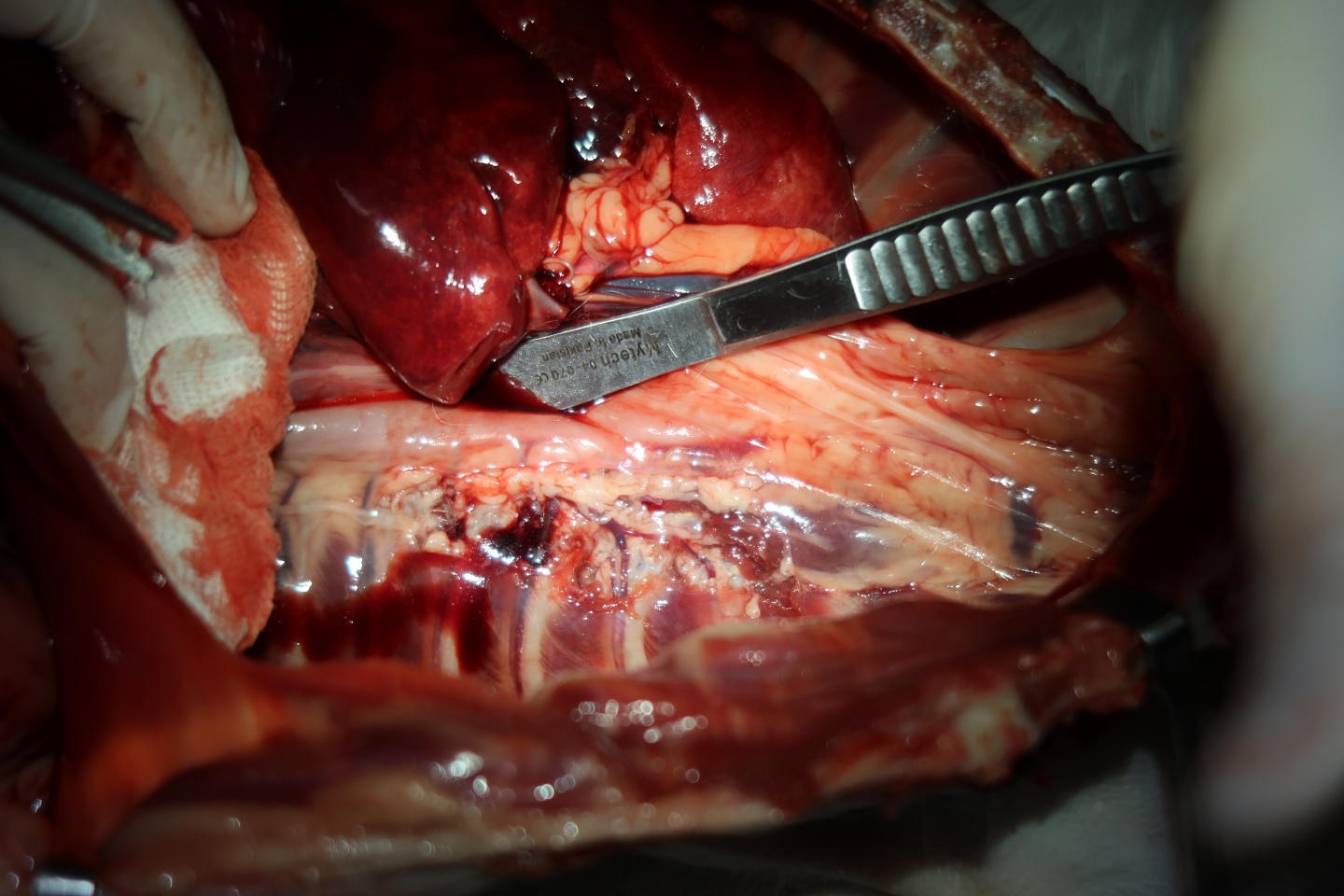

Supplement: Supplementary file 1 [file vetsci-12-01045-s001.zip › KakaoTalk_20221022_163449407_23.jpg]

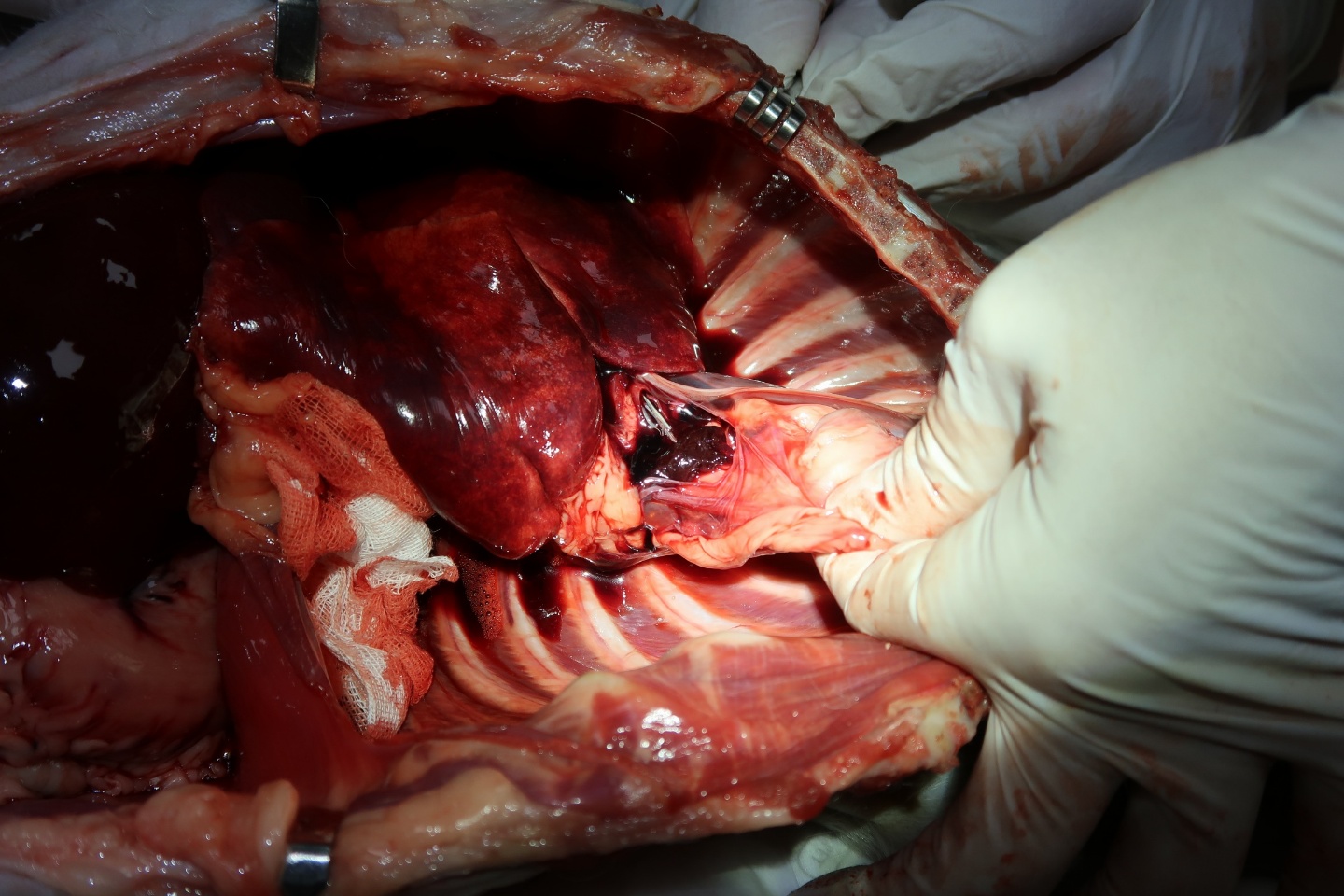

Supplement: Supplementary file 1 [file vetsci-12-01045-s001.zip › KakaoTalk_20221022_163449407_24.jpg]

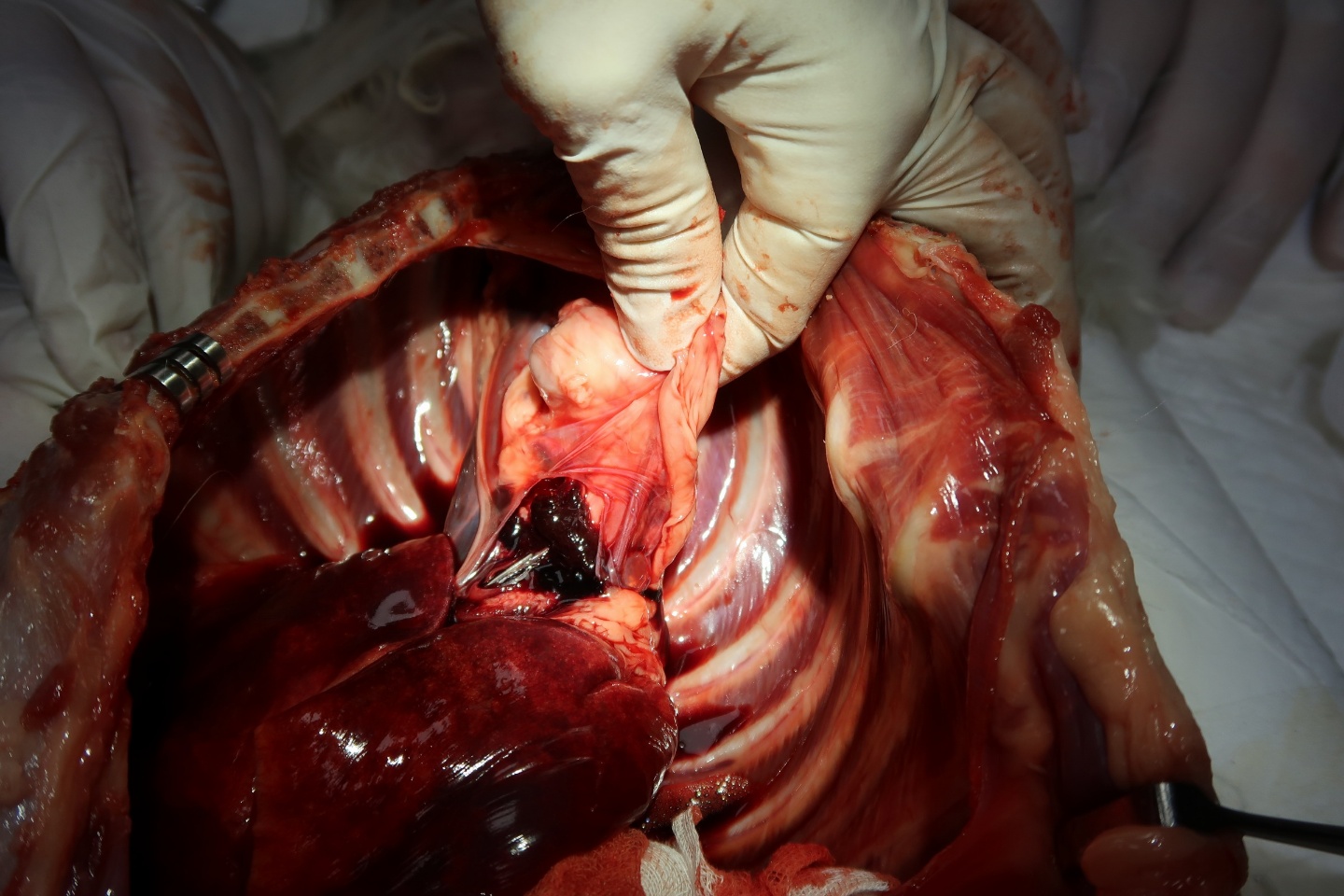

Supplement: Supplementary file 1 [file vetsci-12-01045-s001.zip › KakaoTalk_20221022_163449407_25.jpg]

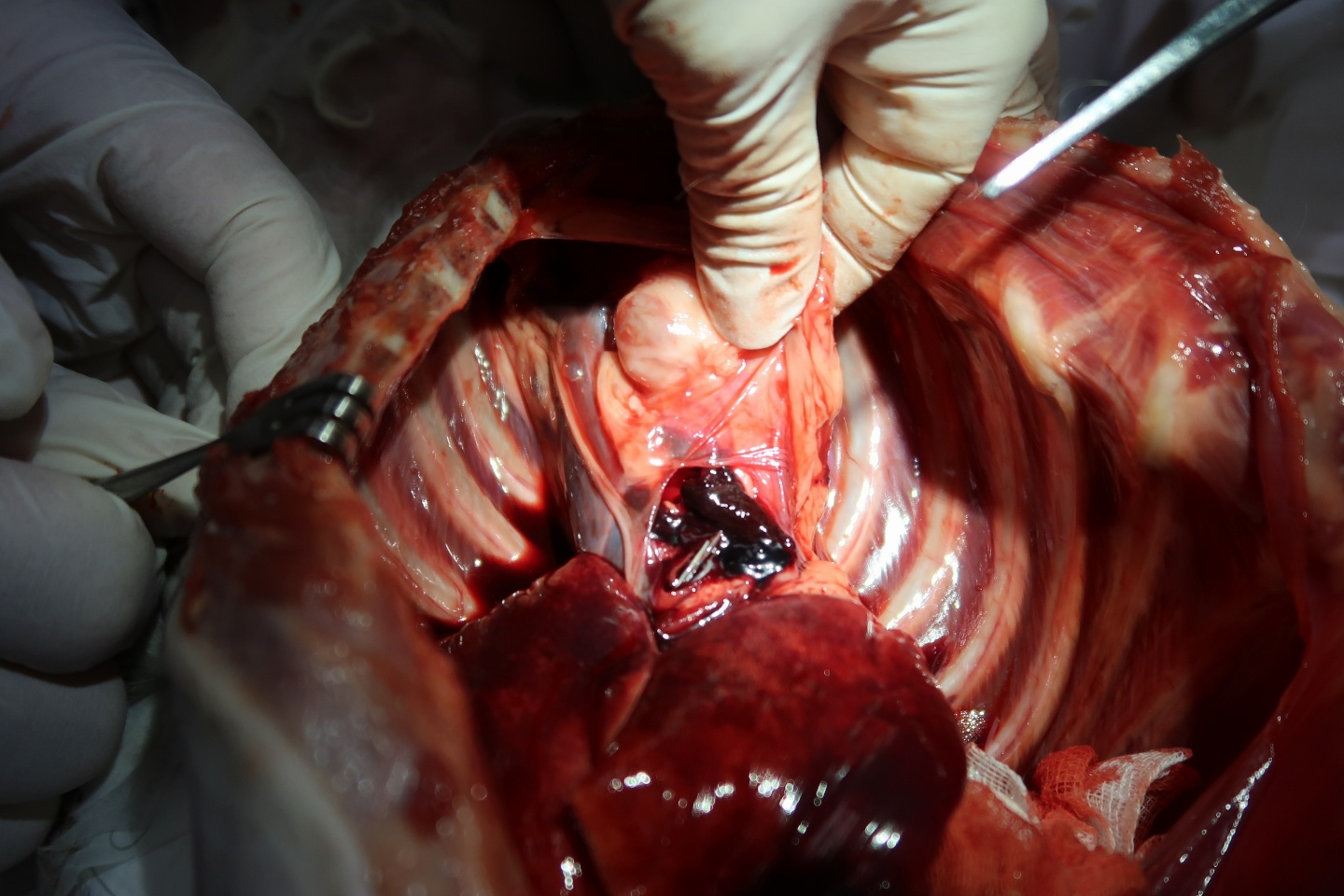

Supplement: Supplementary file 1 [file vetsci-12-01045-s001.zip › KakaoTalk_20221022_163449407_26.jpg]

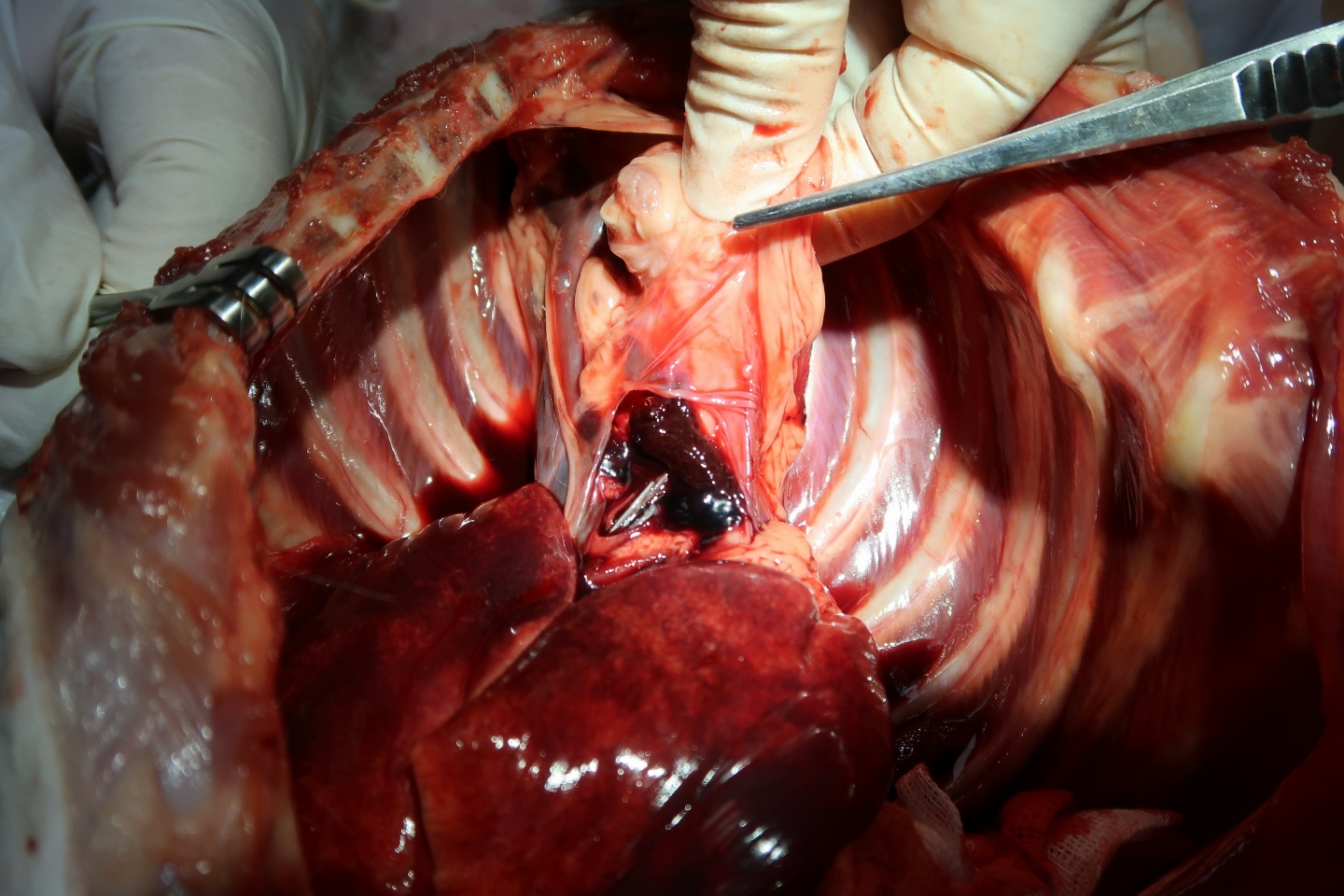

Supplement: Supplementary file 1 [file vetsci-12-01045-s001.zip › KakaoTalk_20221022_163449407_27.jpg]

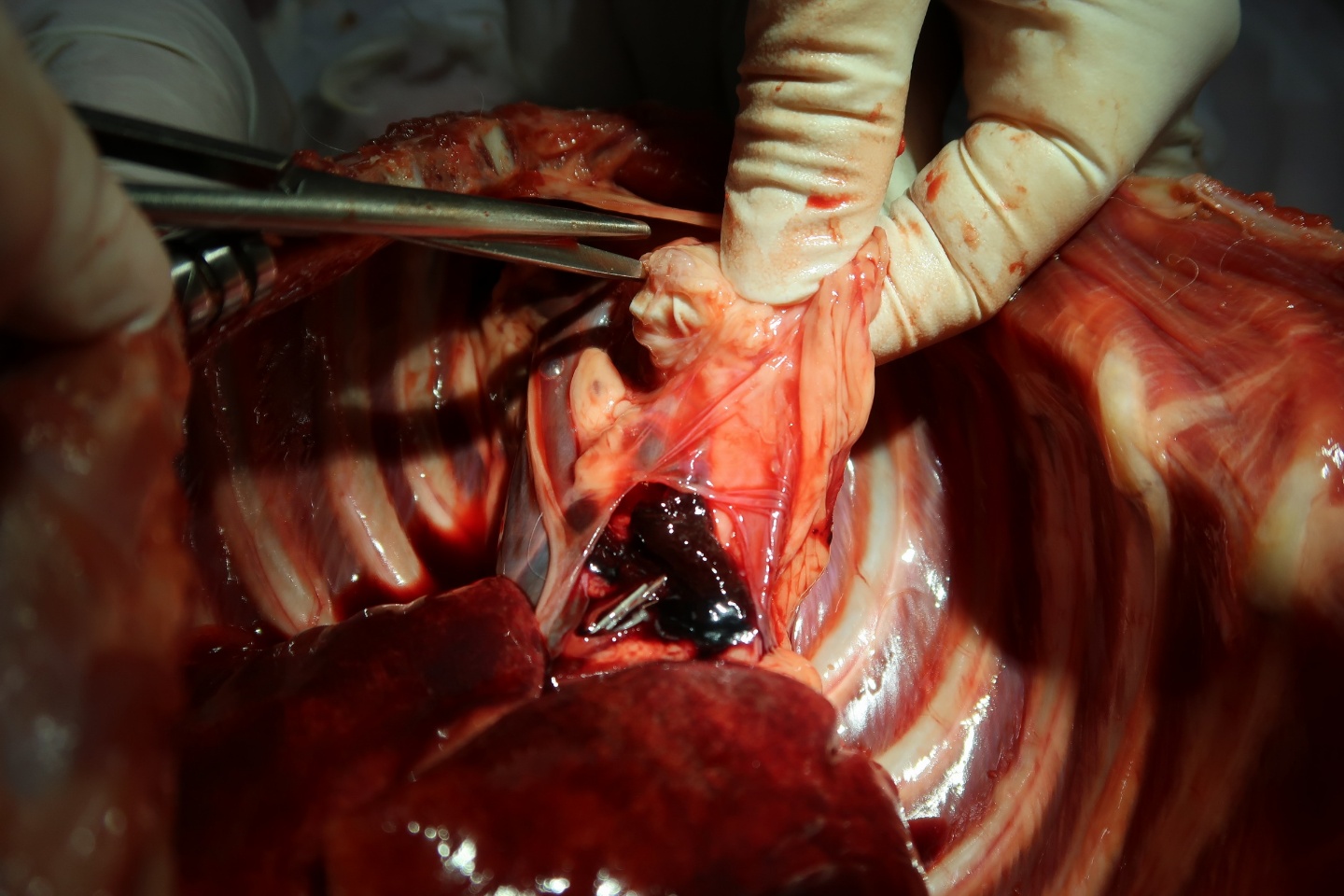

Supplement: Supplementary file 1 [file vetsci-12-01045-s001.zip › KakaoTalk_20221022_163449407_28.jpg]

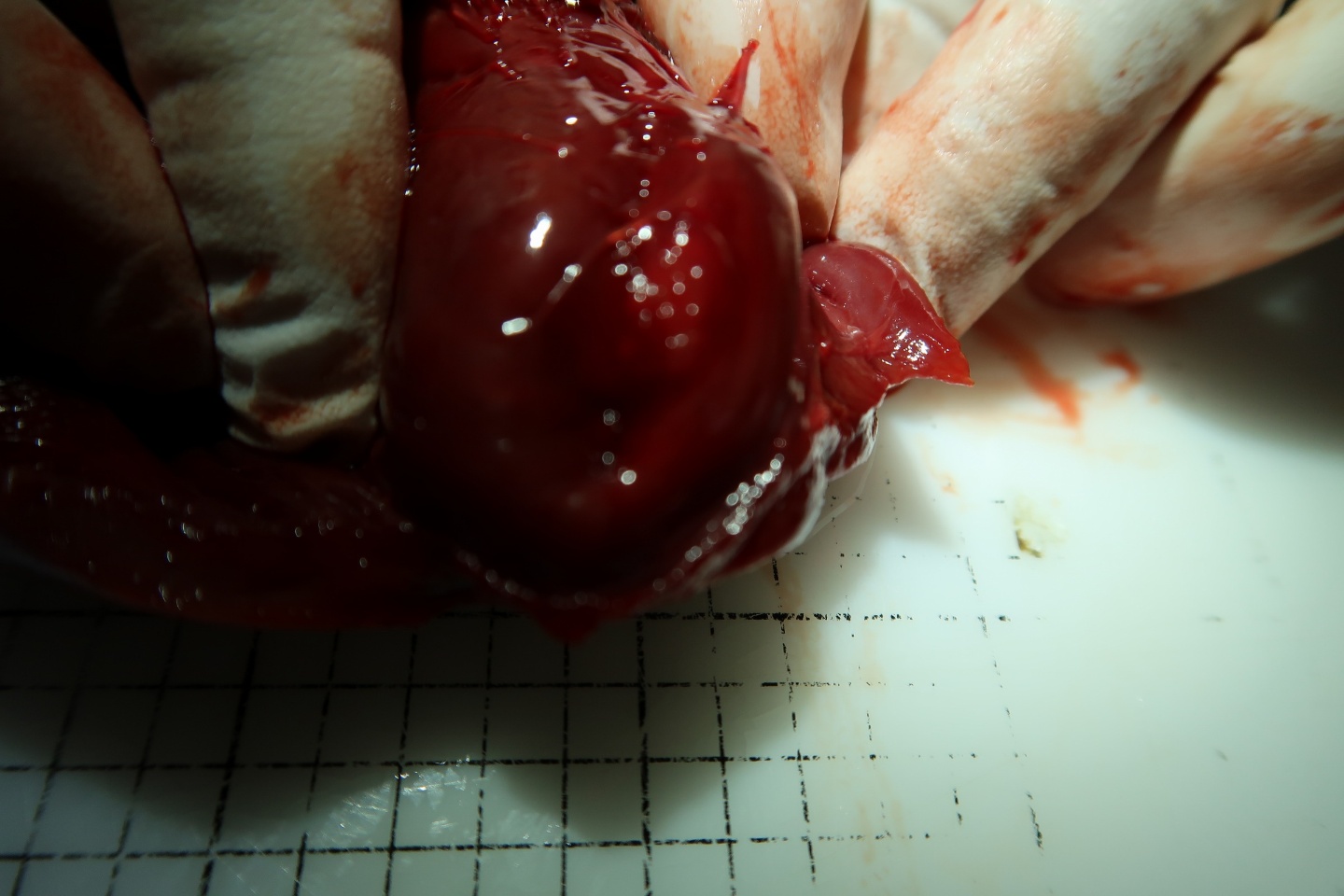

Supplement: Supplementary file 1 [file vetsci-12-01045-s001.zip › KakaoTalk_20221022_163449407_29.jpg]

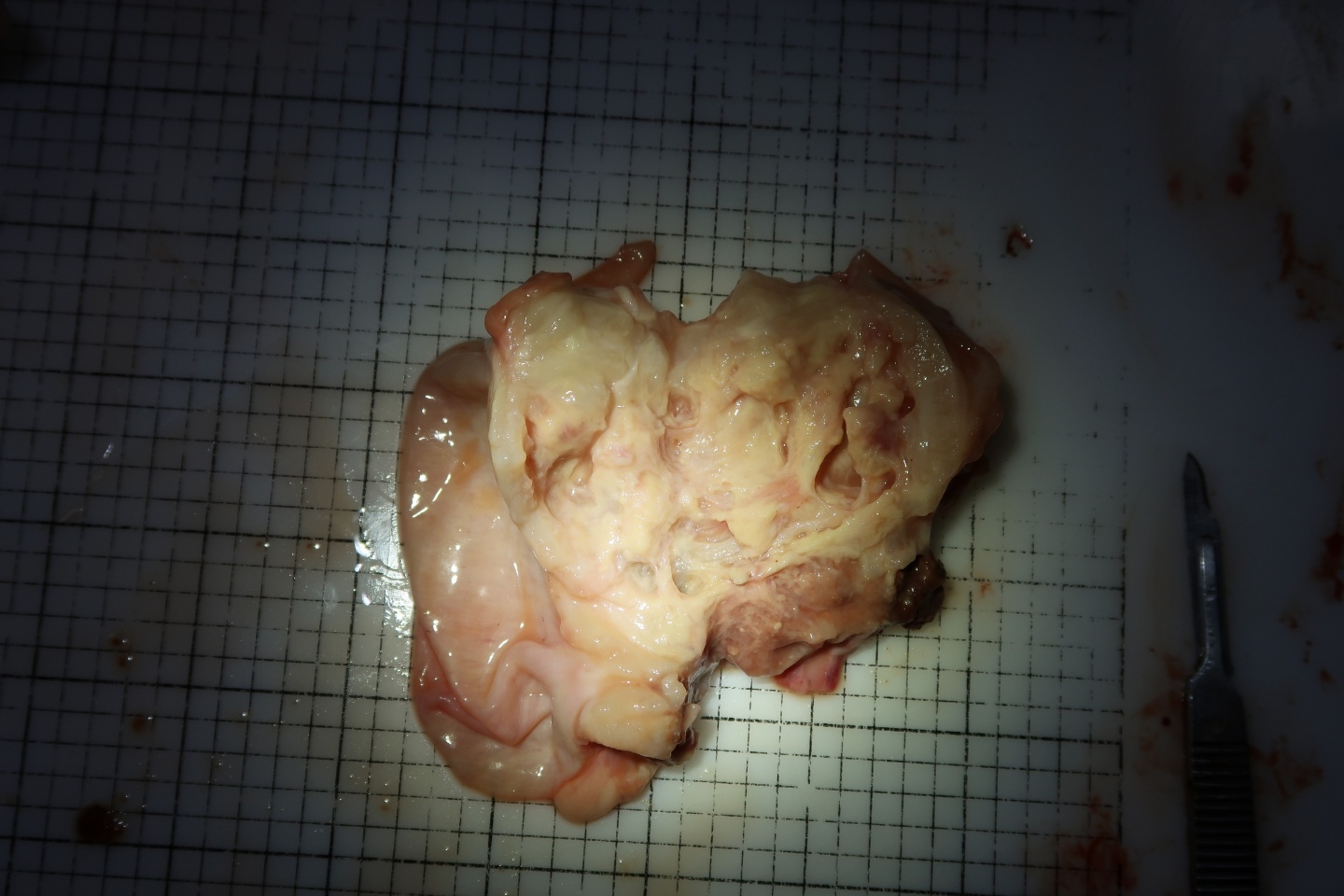

Supplement: Supplementary file 1 [file vetsci-12-01045-s001.zip › KakaoTalk_20221022_163714238.jpg]

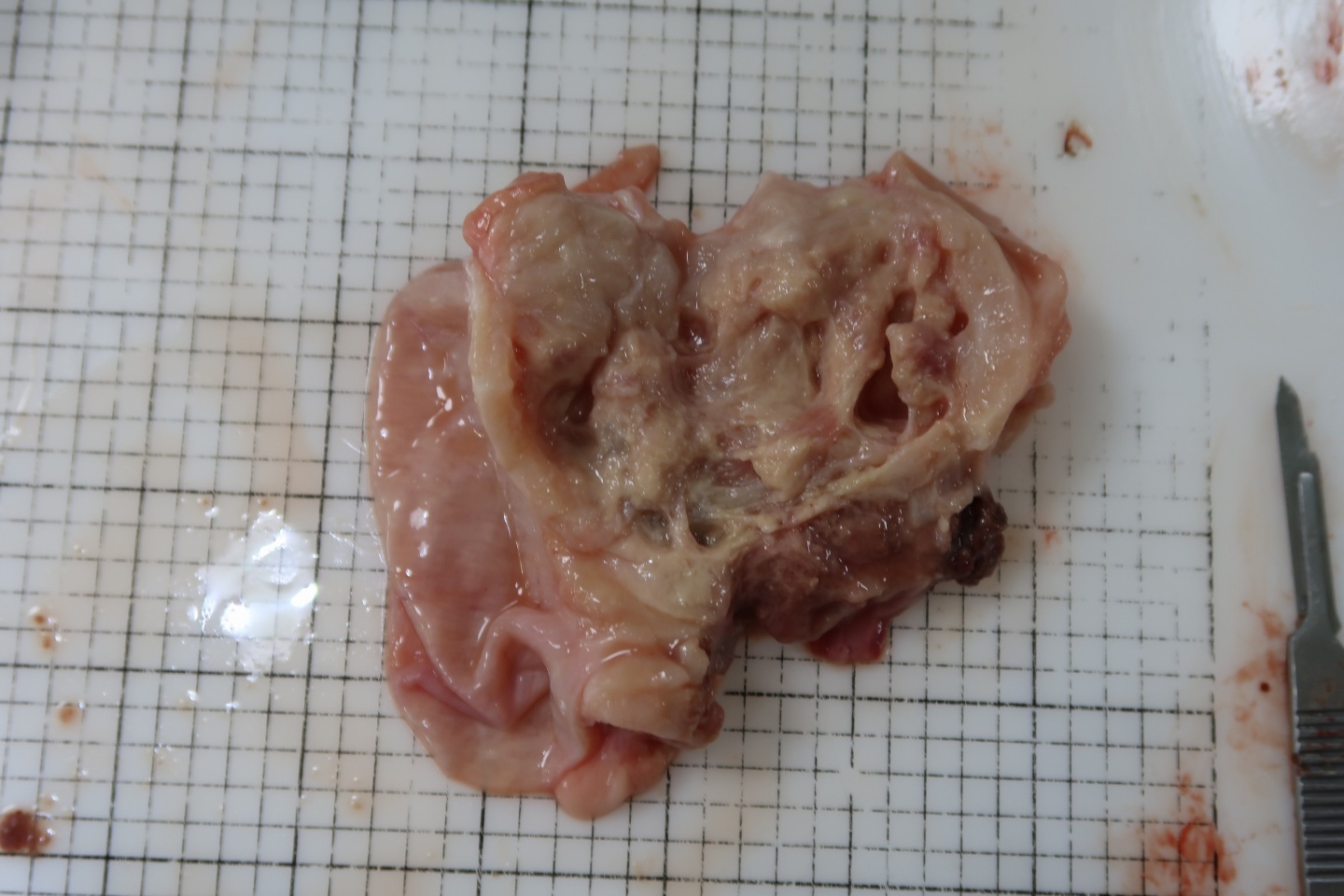

Supplement: Supplementary file 1 [file vetsci-12-01045-s001.zip › KakaoTalk_20221022_163714238_01.jpg]

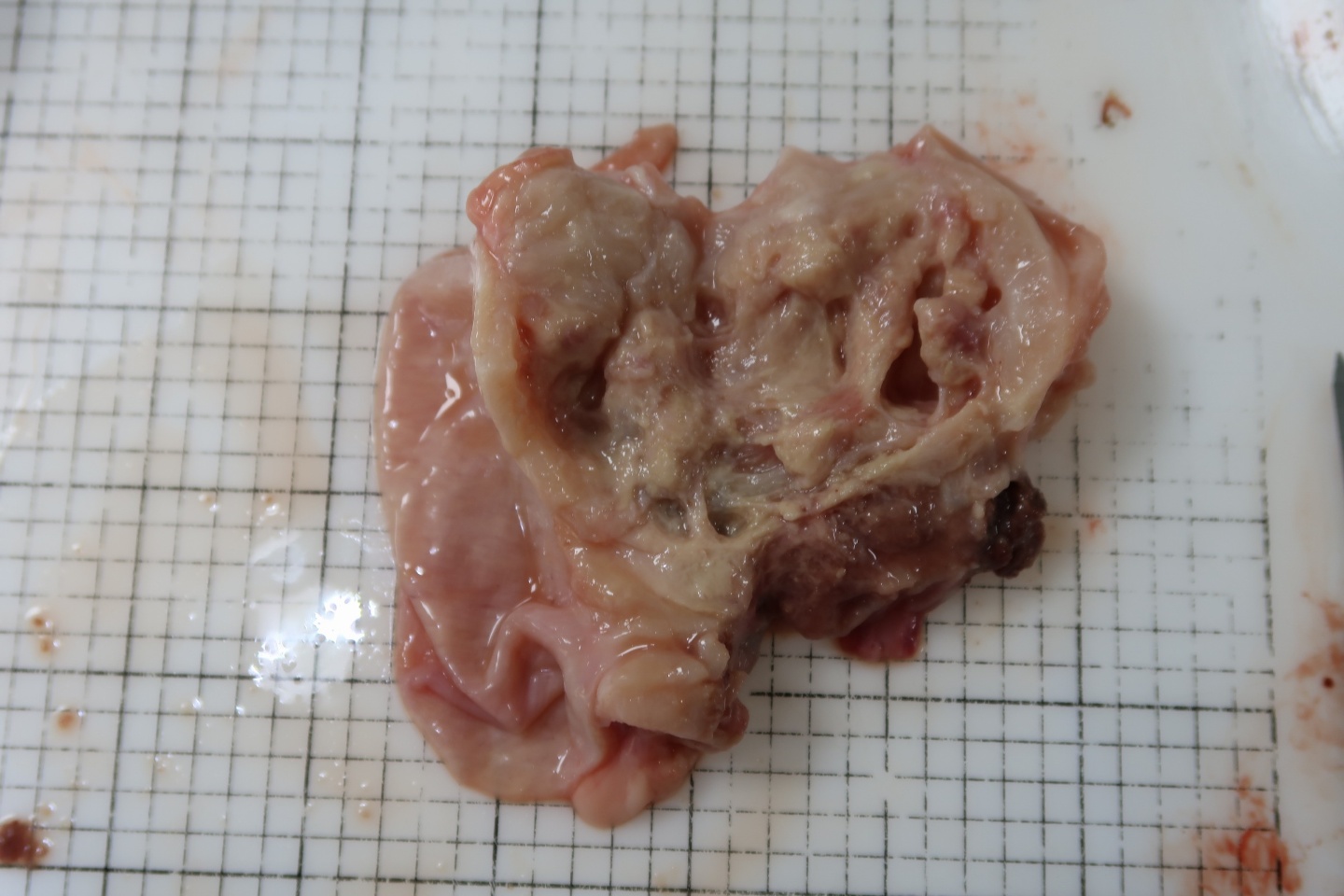

Supplement: Supplementary file 1 [file vetsci-12-01045-s001.zip › KakaoTalk_20221022_163714238_02.jpg]

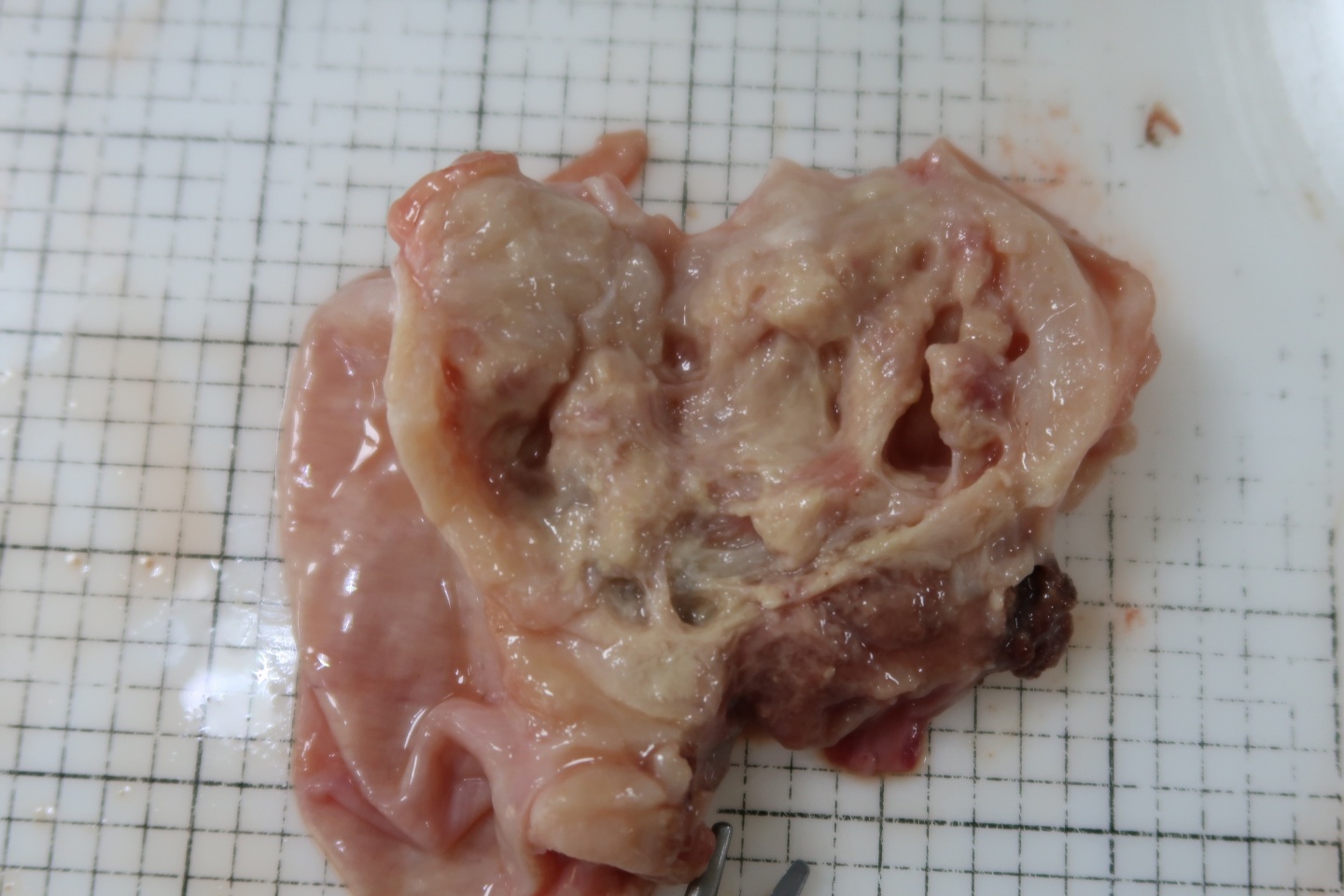

Supplement: Supplementary file 1 [file vetsci-12-01045-s001.zip › KakaoTalk_20221022_163714238_03.jpg]

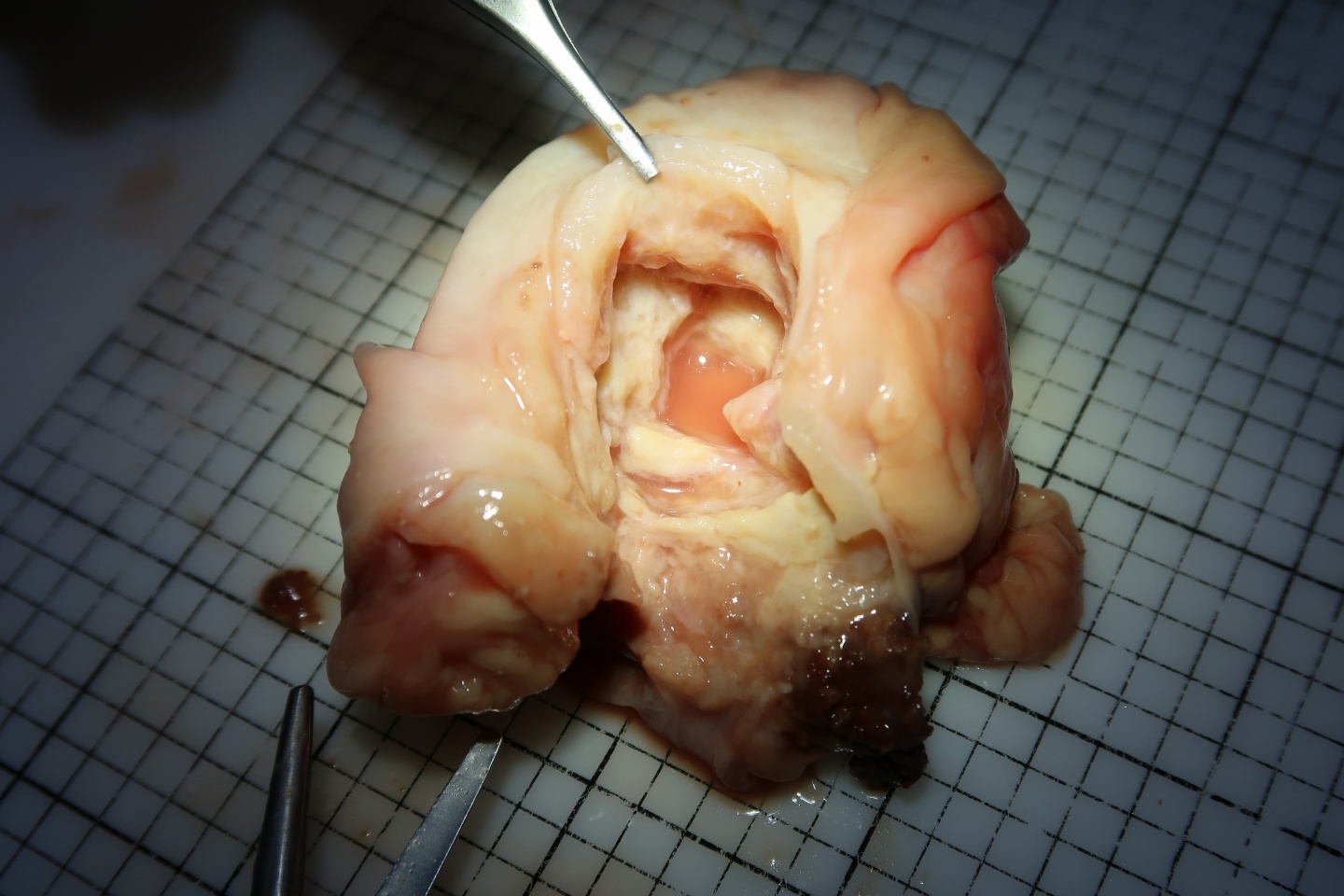

Supplement: Supplementary file 1 [file vetsci-12-01045-s001.zip › KakaoTalk_20221022_163714238_04.jpg]

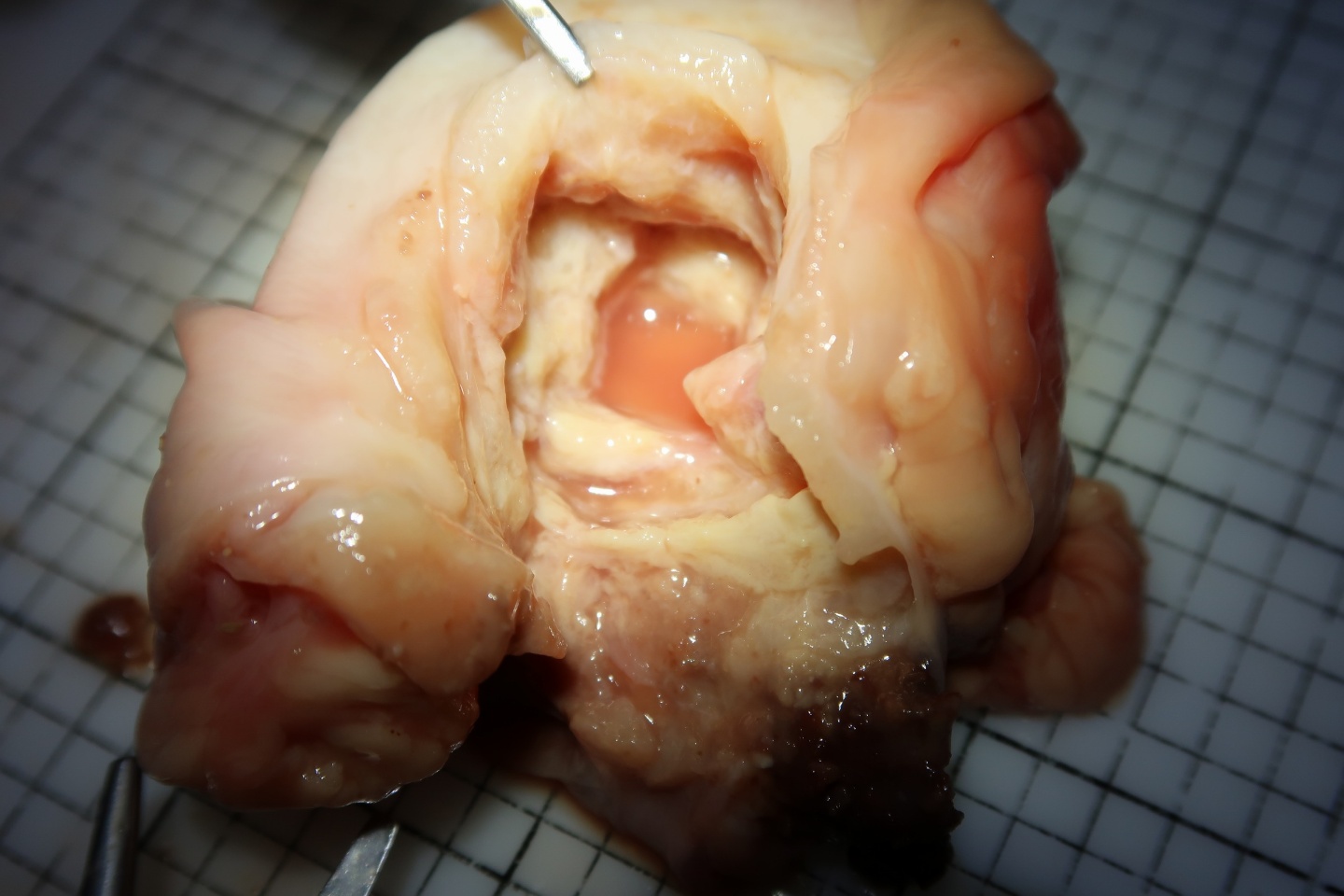

Supplement: Supplementary file 1 [file vetsci-12-01045-s001.zip › KakaoTalk_20221022_163714238_05.jpg]

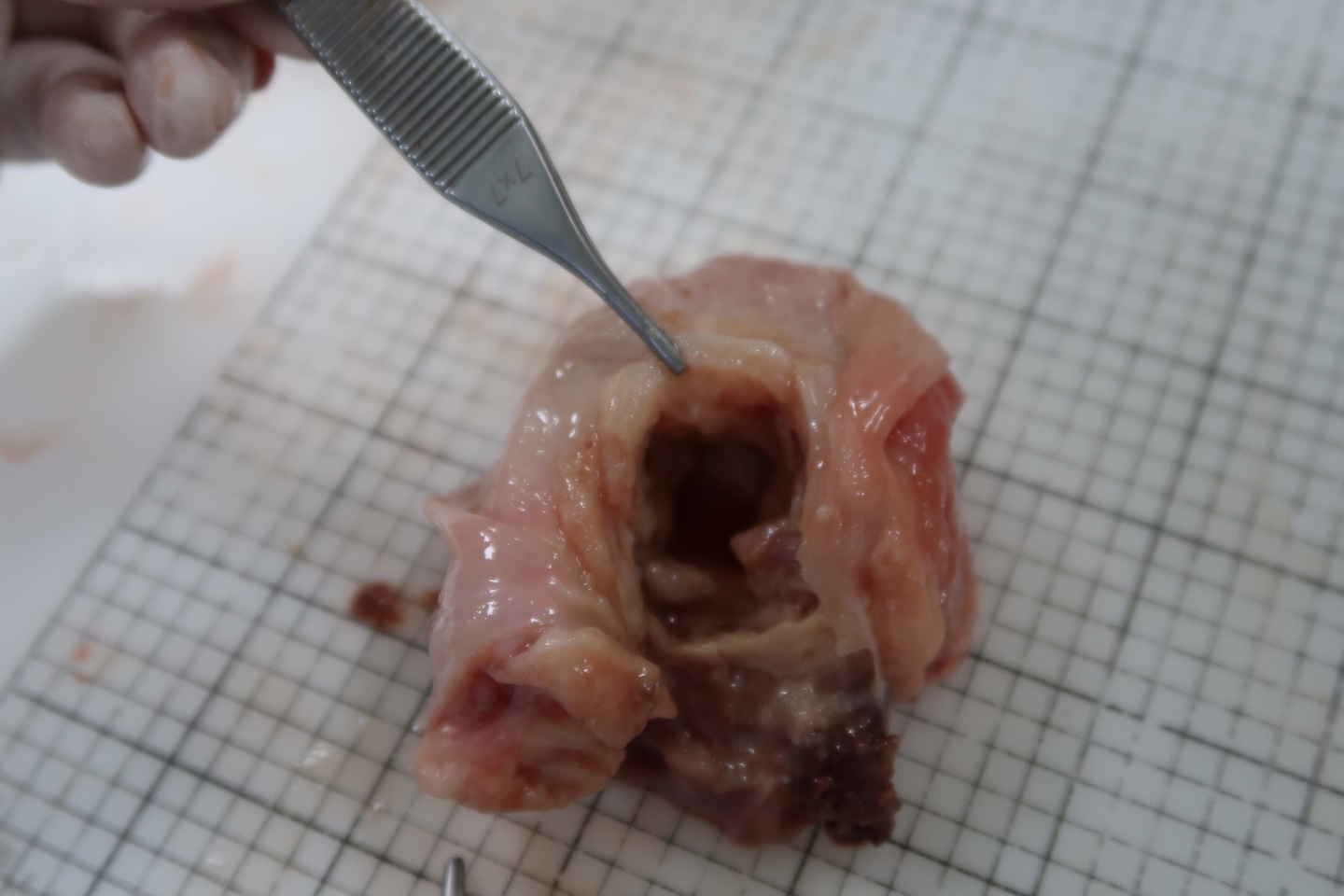

Supplement: Supplementary file 1 [file vetsci-12-01045-s001.zip › KakaoTalk_20221022_163714238_06.jpg]

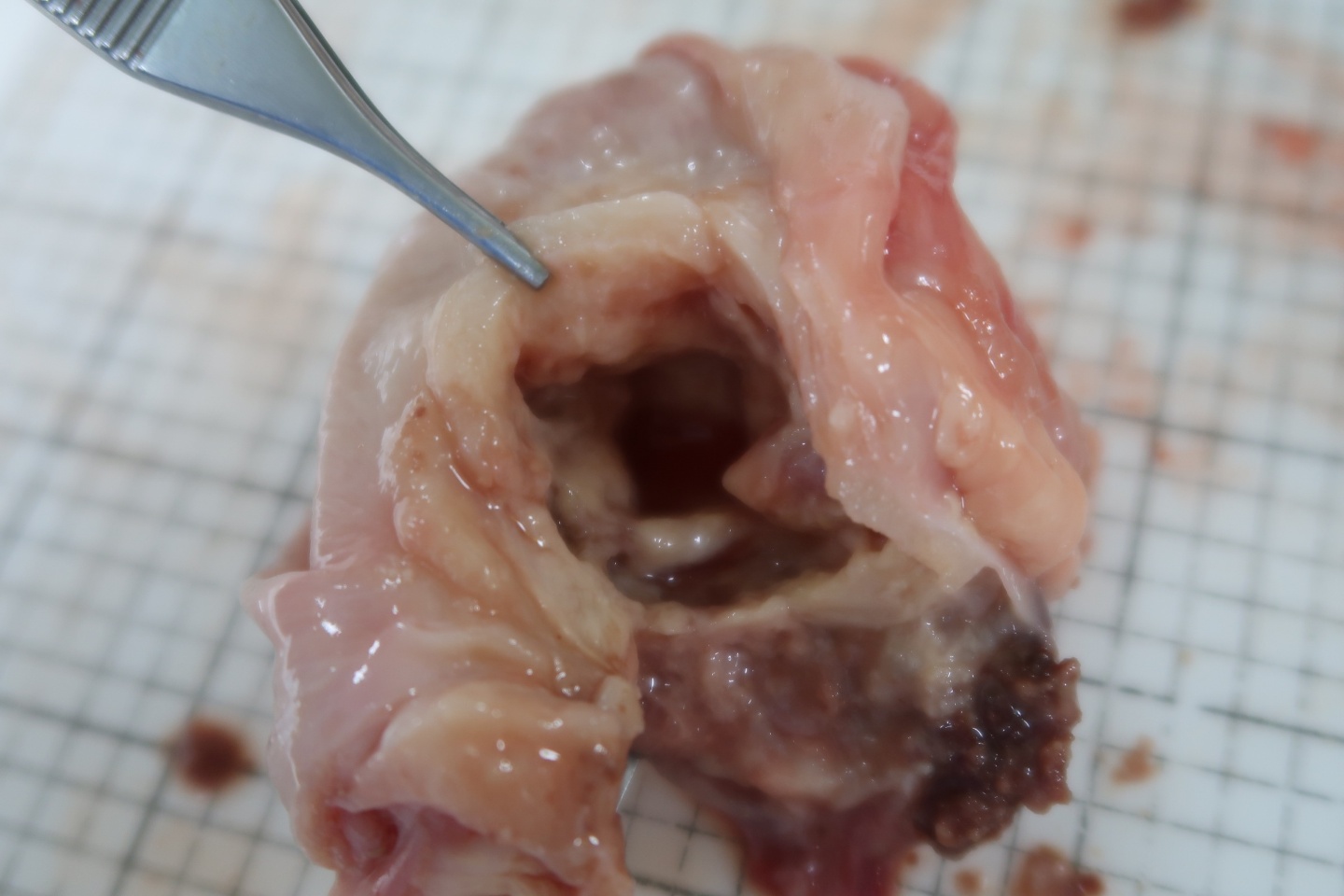

Supplement: Supplementary file 1 [file vetsci-12-01045-s001.zip › KakaoTalk_20221022_163714238_07.jpg]

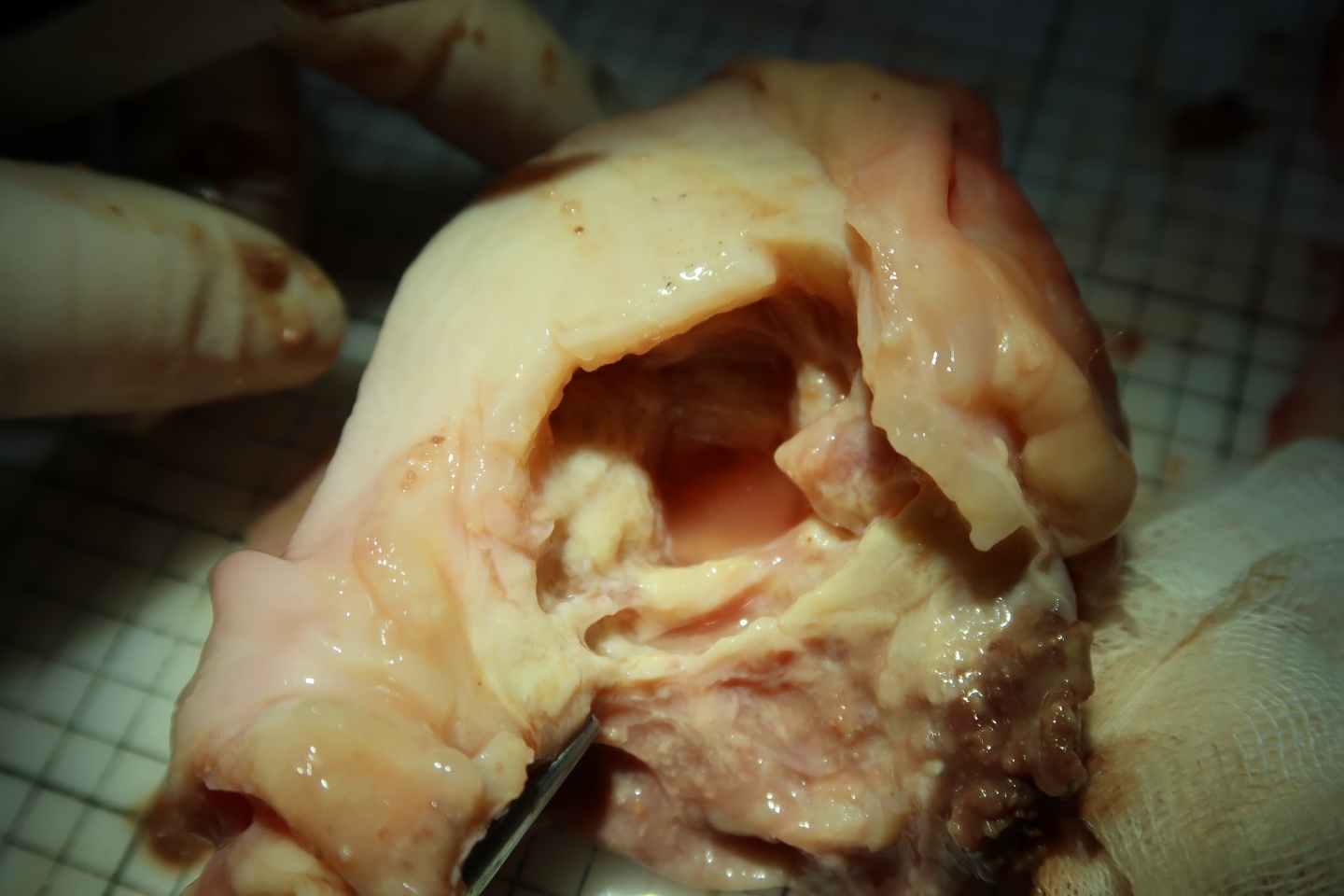

Supplement: Supplementary file 1 [file vetsci-12-01045-s001.zip › KakaoTalk_20221022_163714238_08.jpg]

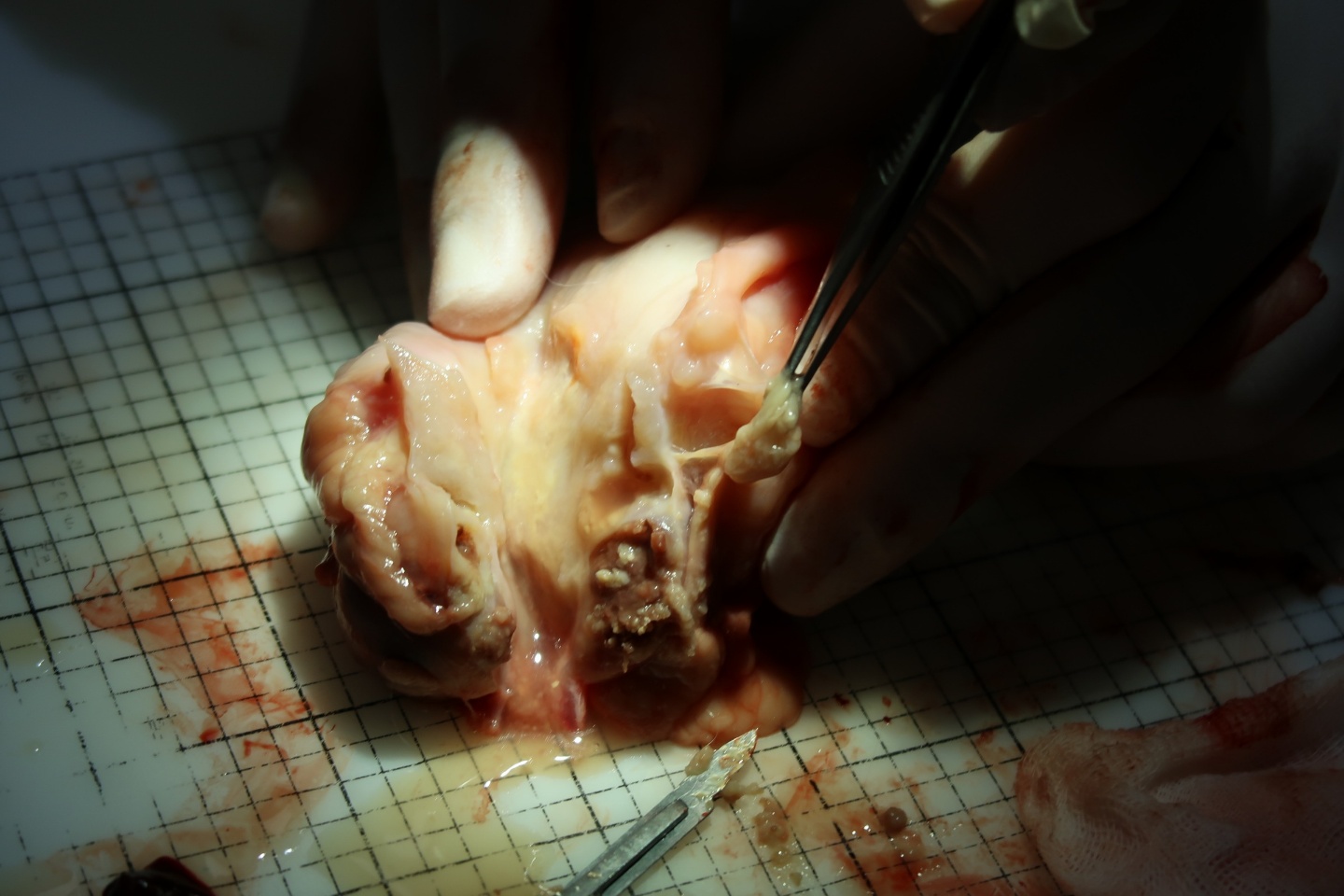

Supplement: Supplementary file 1 [file vetsci-12-01045-s001.zip › KakaoTalk_20221022_163714238_09.jpg]

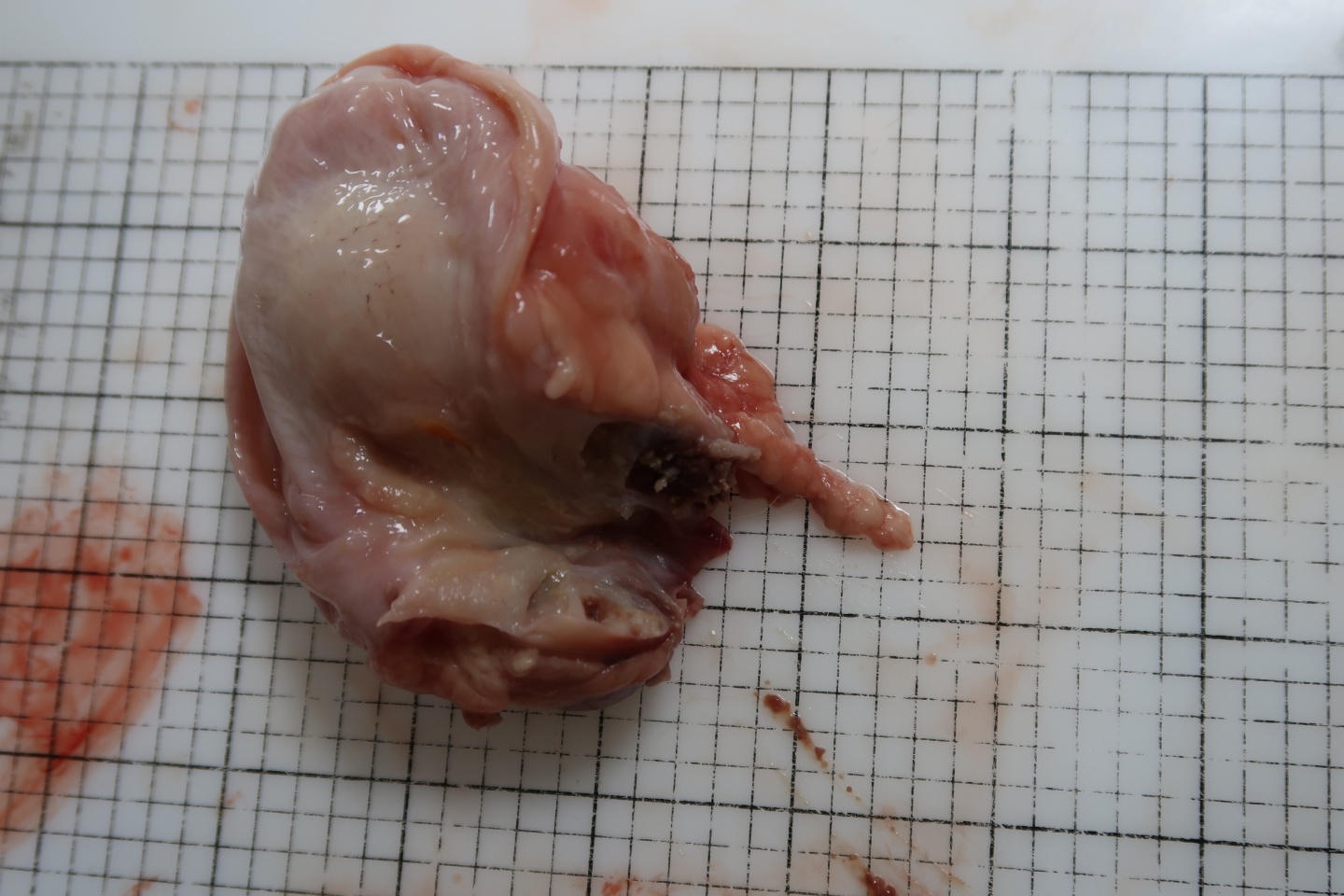

Supplement: Supplementary file 1 [file vetsci-12-01045-s001.zip › KakaoTalk_20221022_163714238_10.jpg]

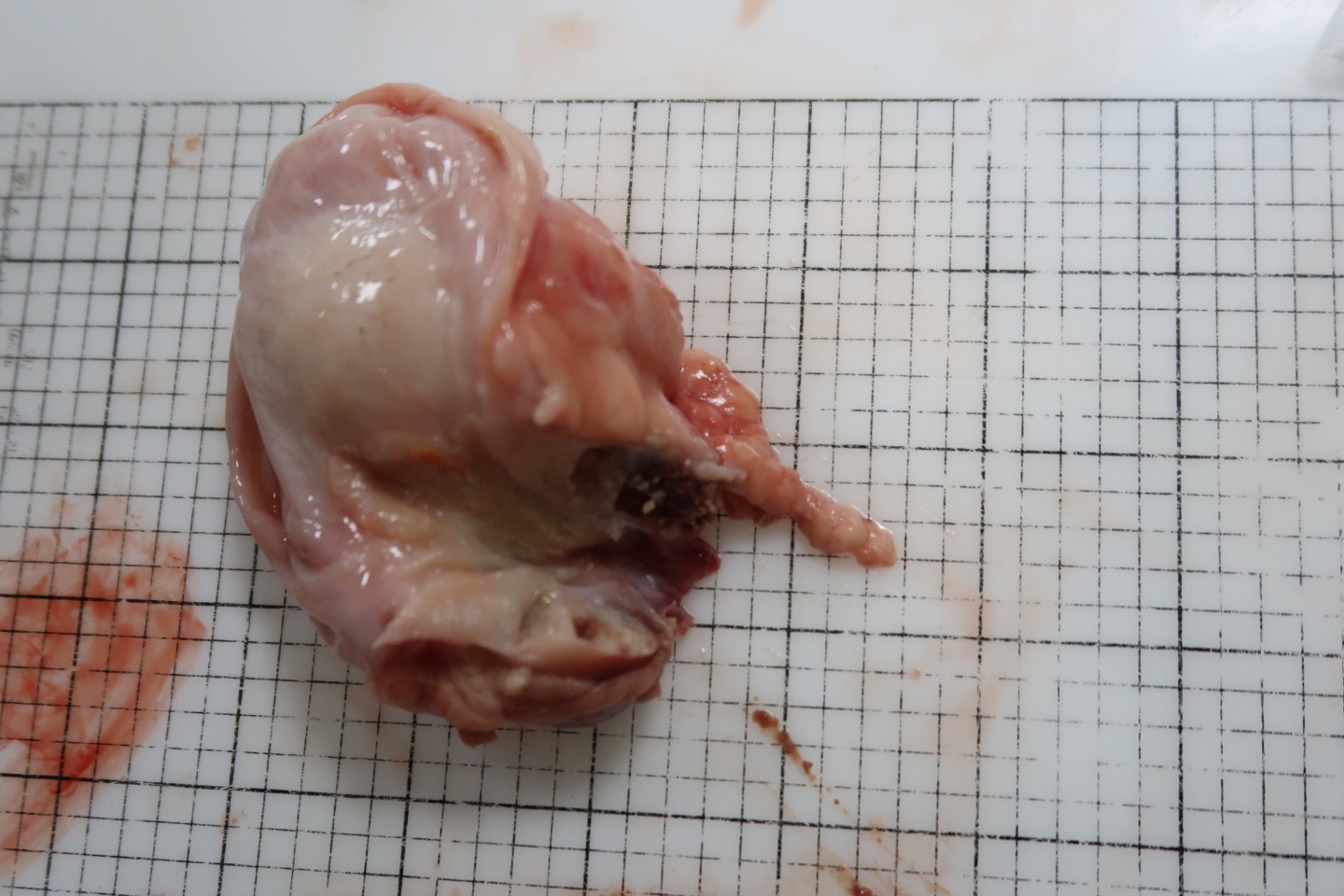

Supplement: Supplementary file 1 [file vetsci-12-01045-s001.zip › KakaoTalk_20221022_163714238_11.jpg]

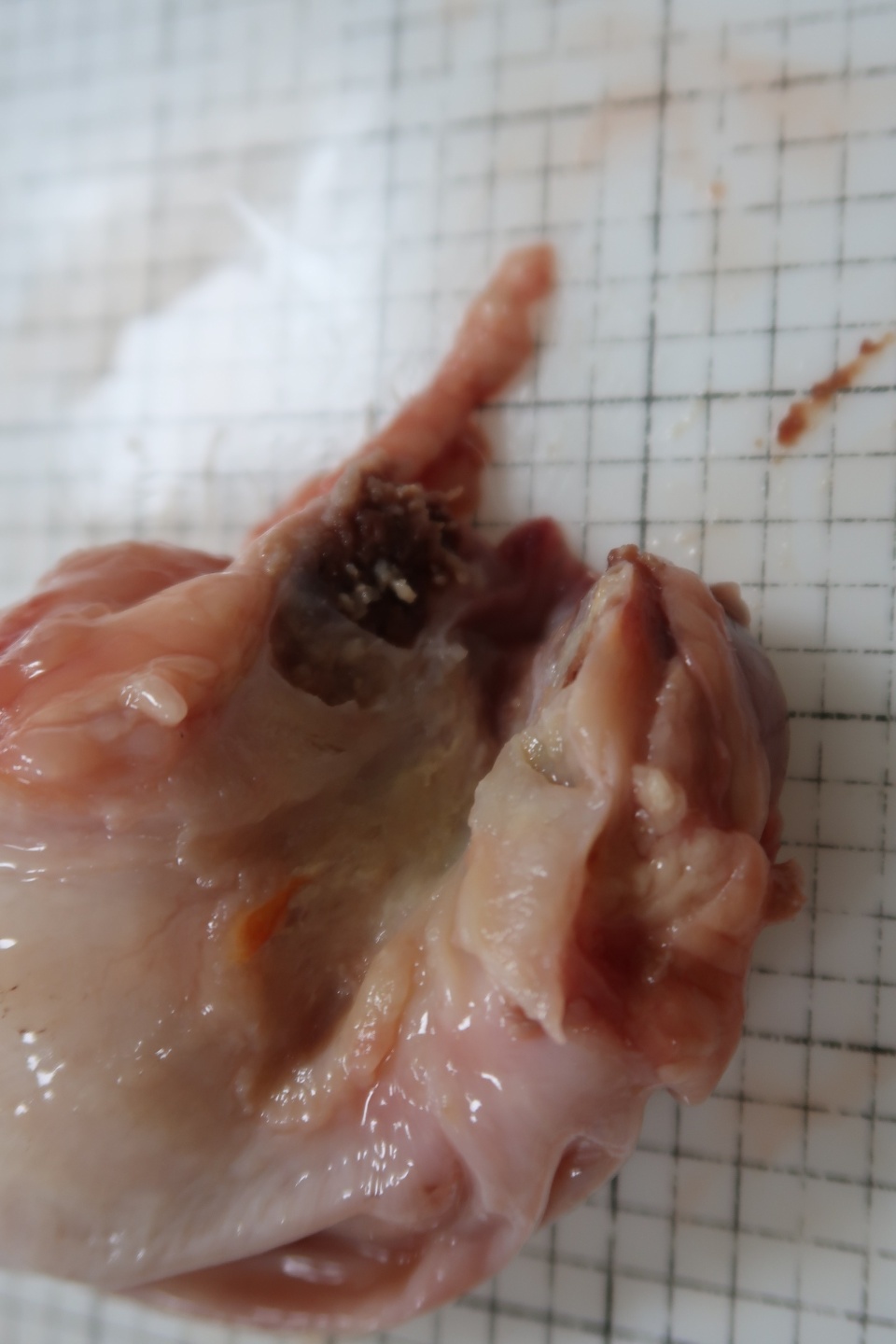

Supplement: Supplementary file 1 [file vetsci-12-01045-s001.zip › KakaoTalk_20221022_163714238_12.jpg]

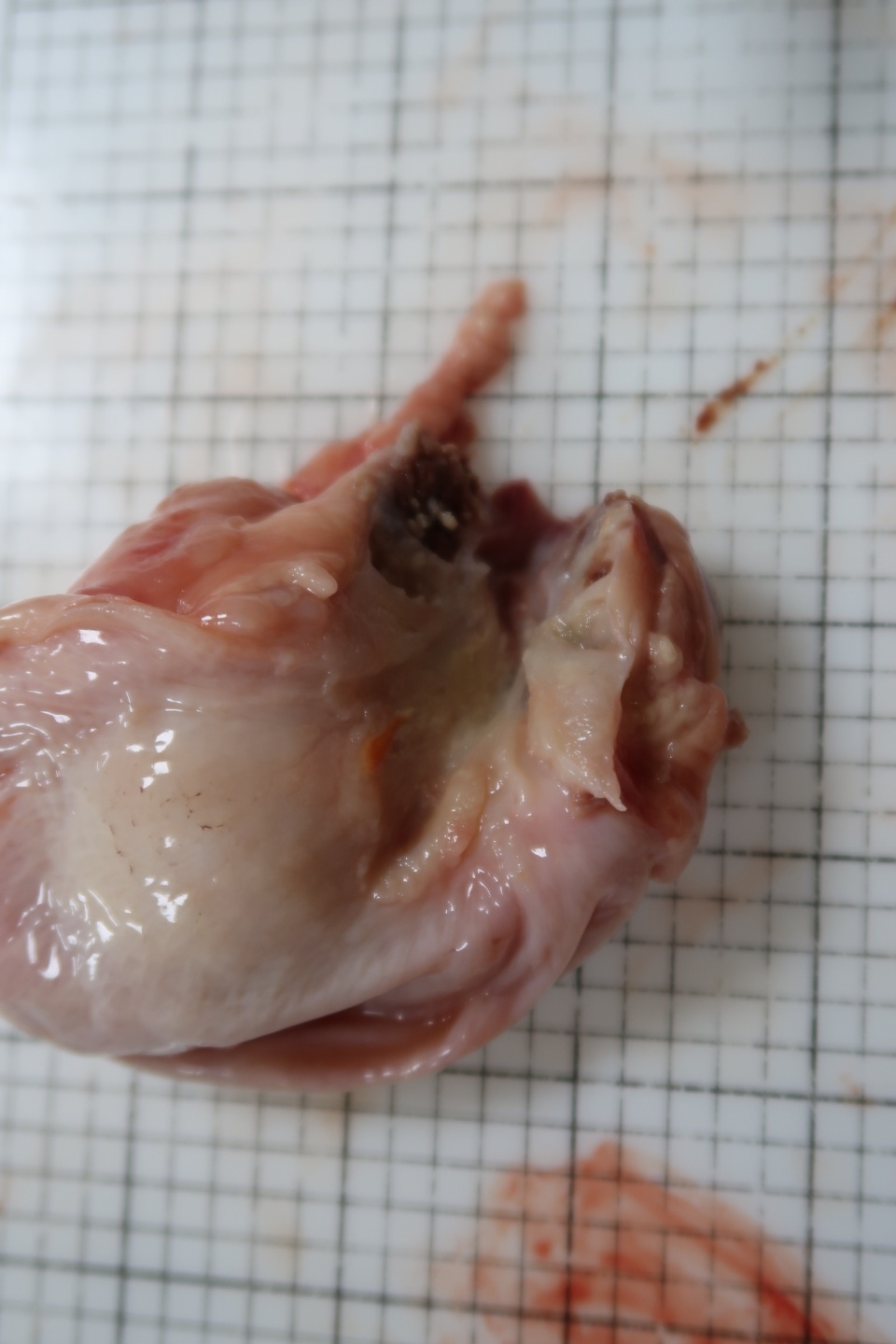

Supplement: Supplementary file 1 [file vetsci-12-01045-s001.zip › KakaoTalk_20221022_163714238_13.jpg]

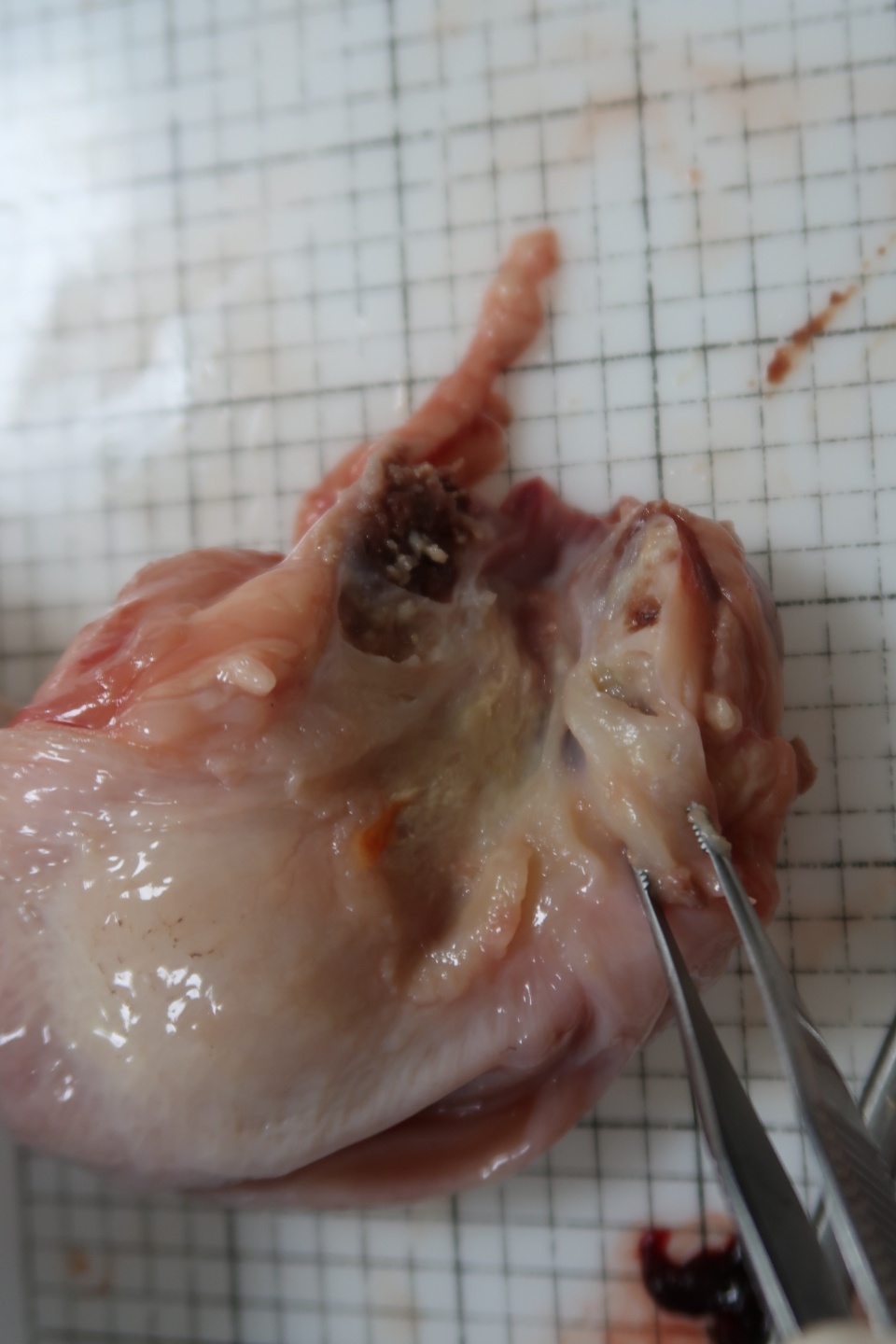

Supplement: Supplementary file 1 [file vetsci-12-01045-s001.zip › KakaoTalk_20221022_163714238_14.jpg]

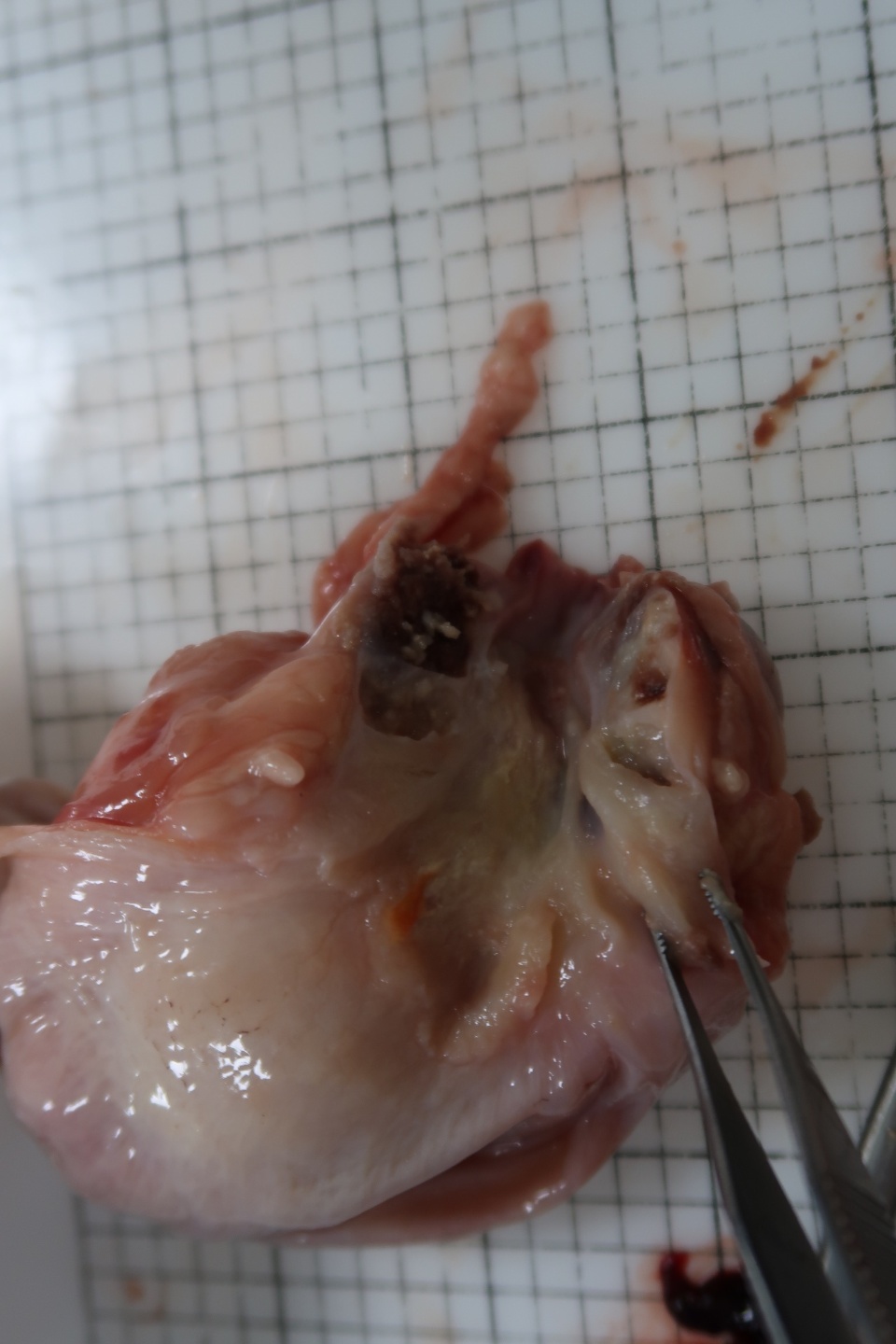

Supplement: Supplementary file 1 [file vetsci-12-01045-s001.zip › KakaoTalk_20221022_163714238_15.jpg]

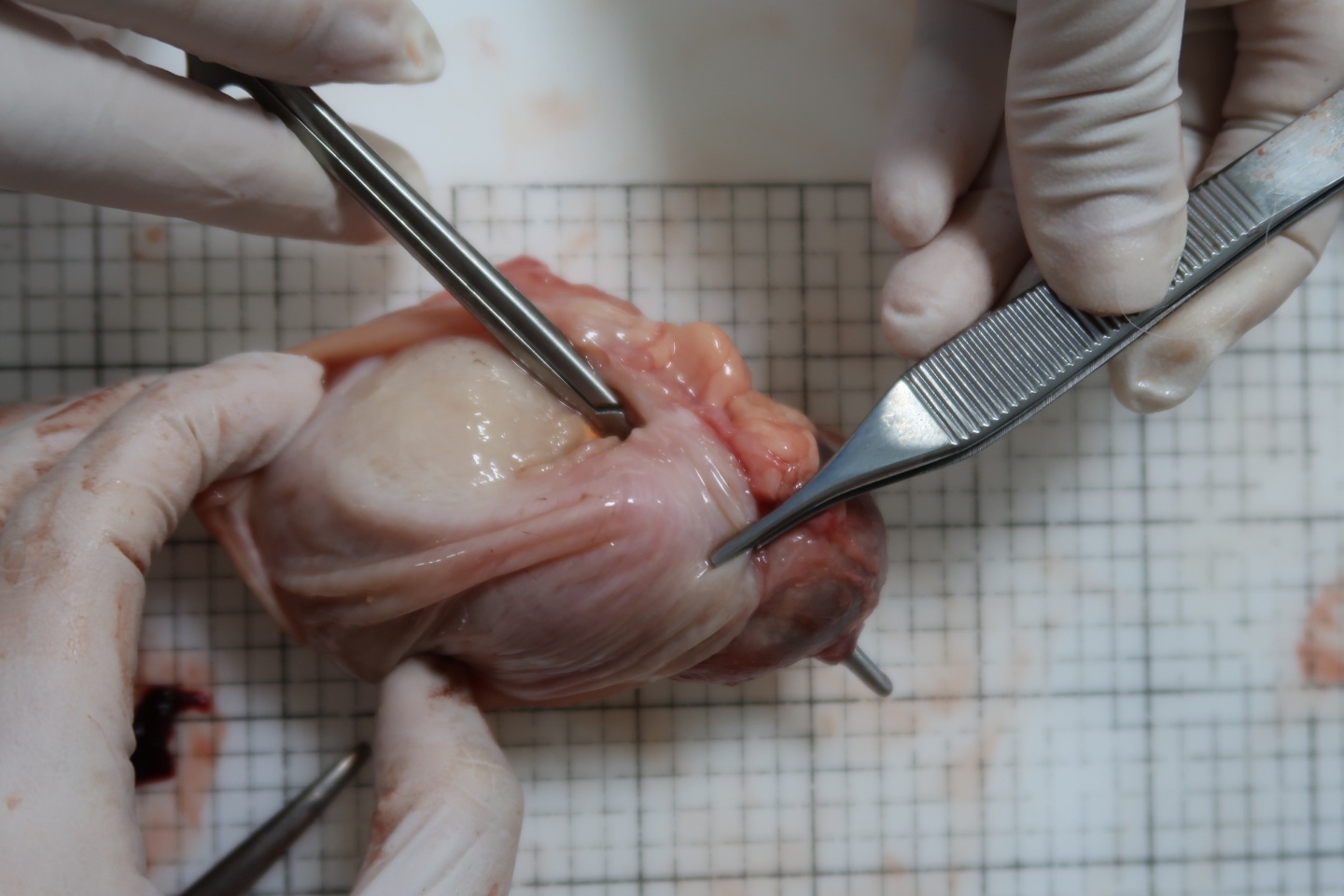

Supplement: Supplementary file 1 [file vetsci-12-01045-s001.zip › KakaoTalk_20221022_163714238_16.jpg]

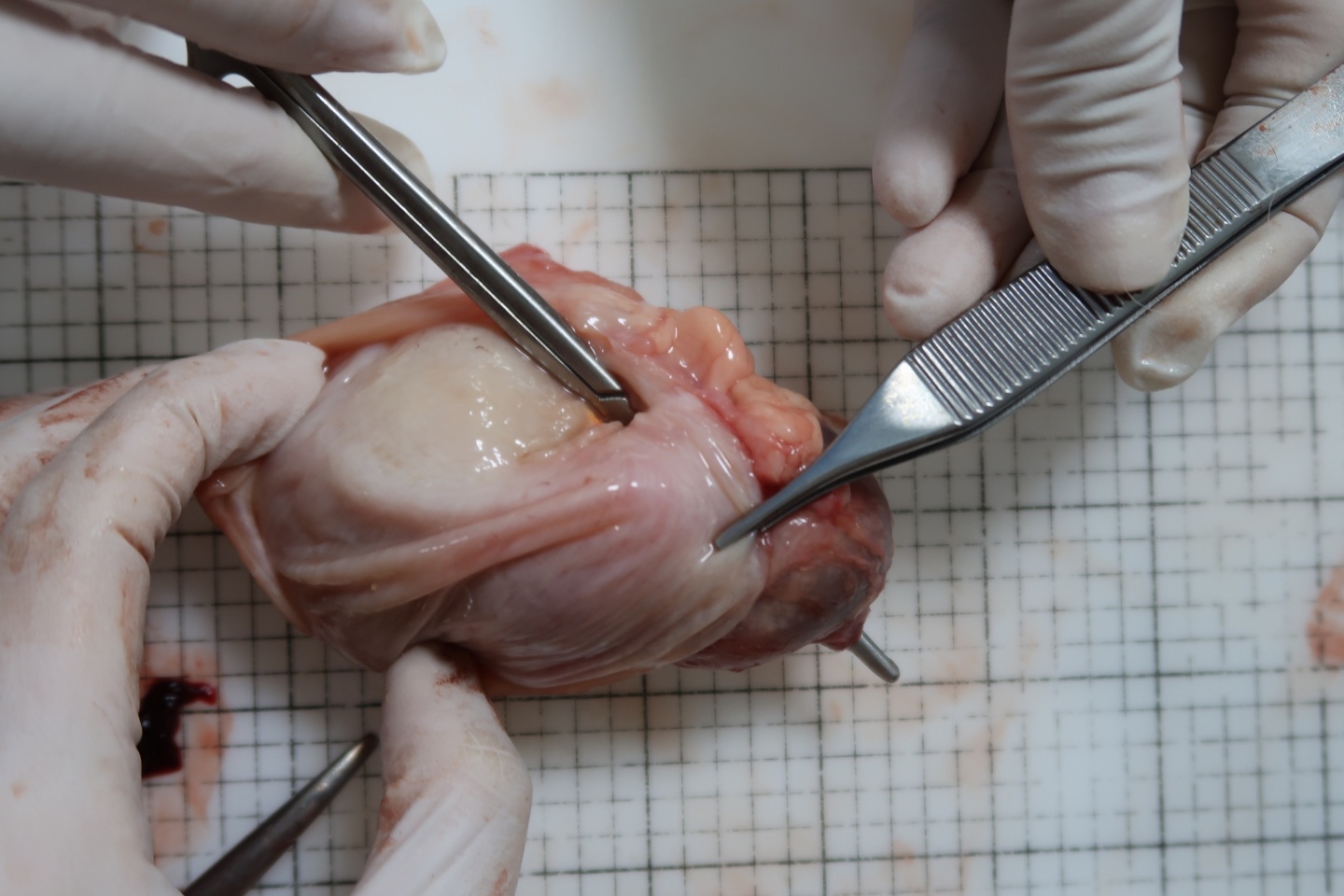

Supplement: Supplementary file 1 [file vetsci-12-01045-s001.zip › KakaoTalk_20221022_163714238_17.jpg]

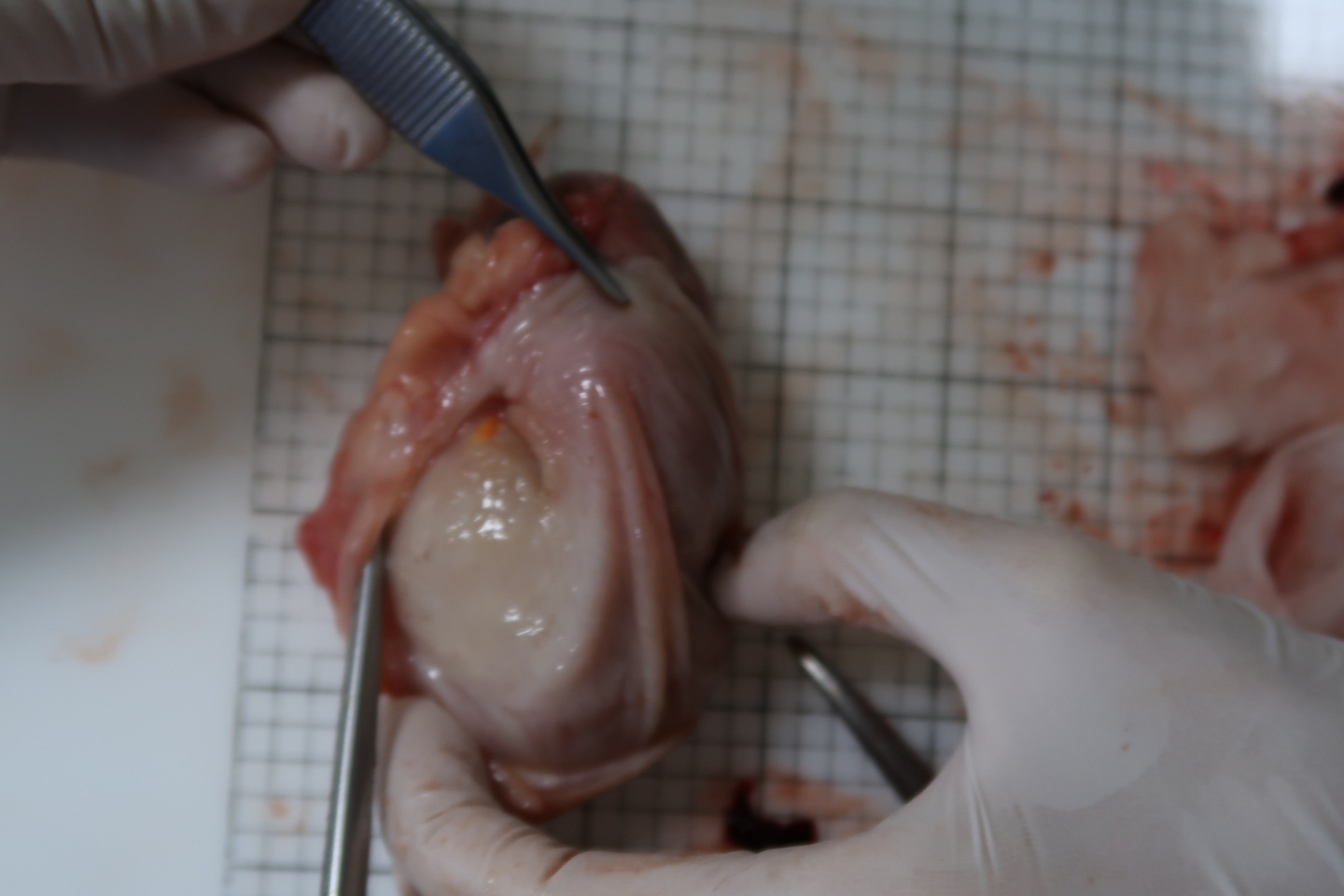

Supplement: Supplementary file 1 [file vetsci-12-01045-s001.zip › KakaoTalk_20221022_163714238_18.jpg]

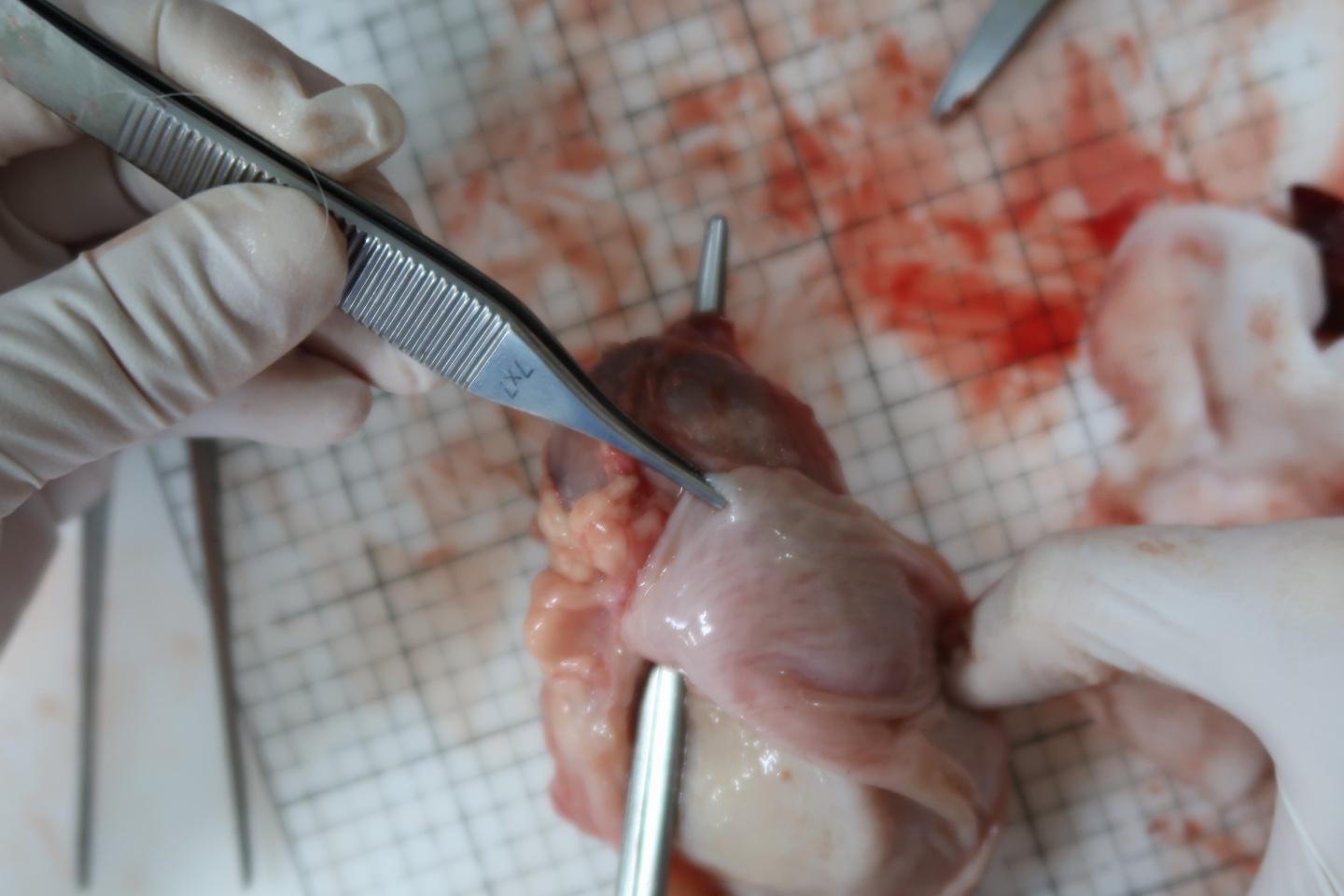

Supplement: Supplementary file 1 [file vetsci-12-01045-s001.zip › KakaoTalk_20221022_163714238_19.jpg]

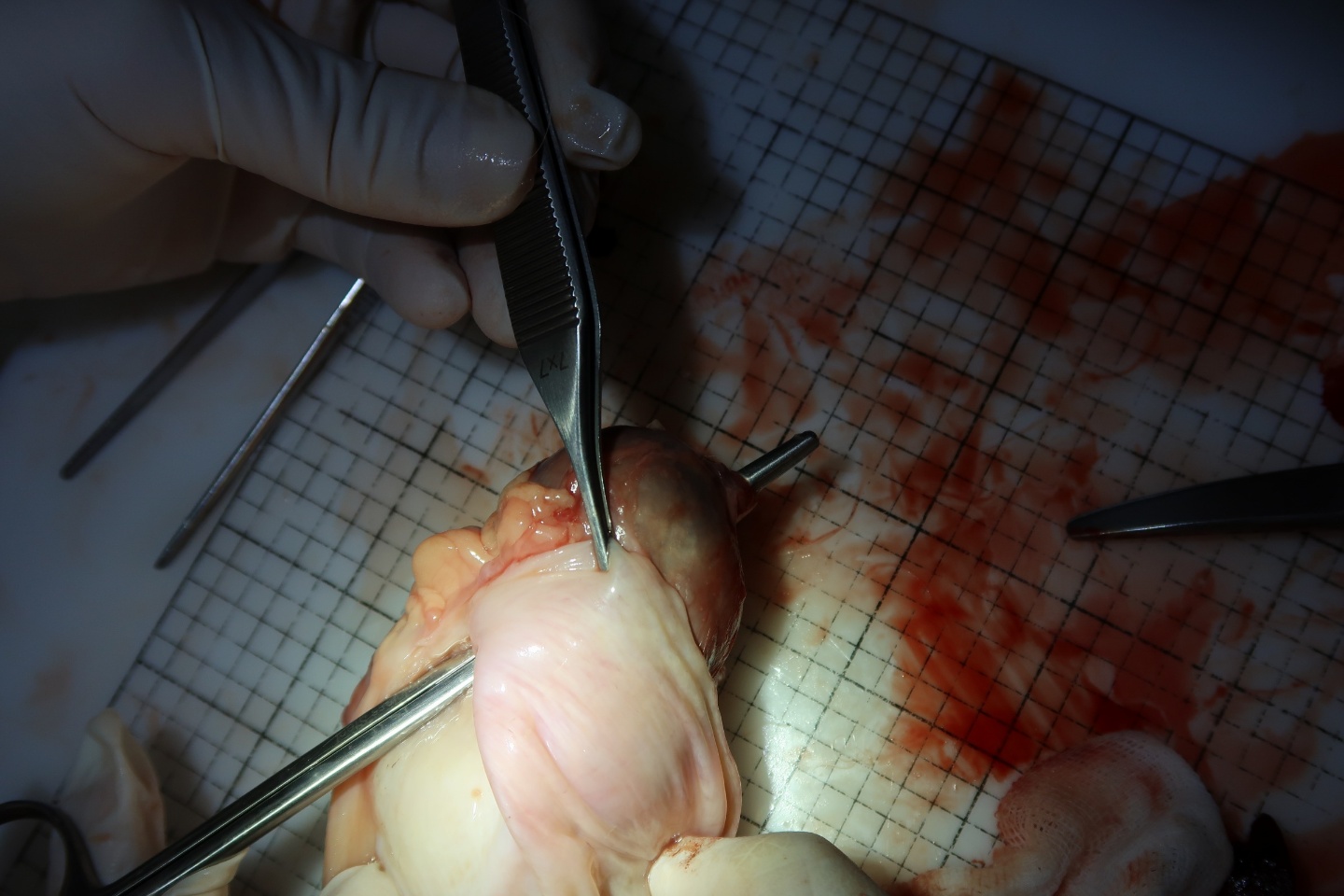

Supplement: Supplementary file 1 [file vetsci-12-01045-s001.zip › KakaoTalk_20221022_163714238_20.jpg]

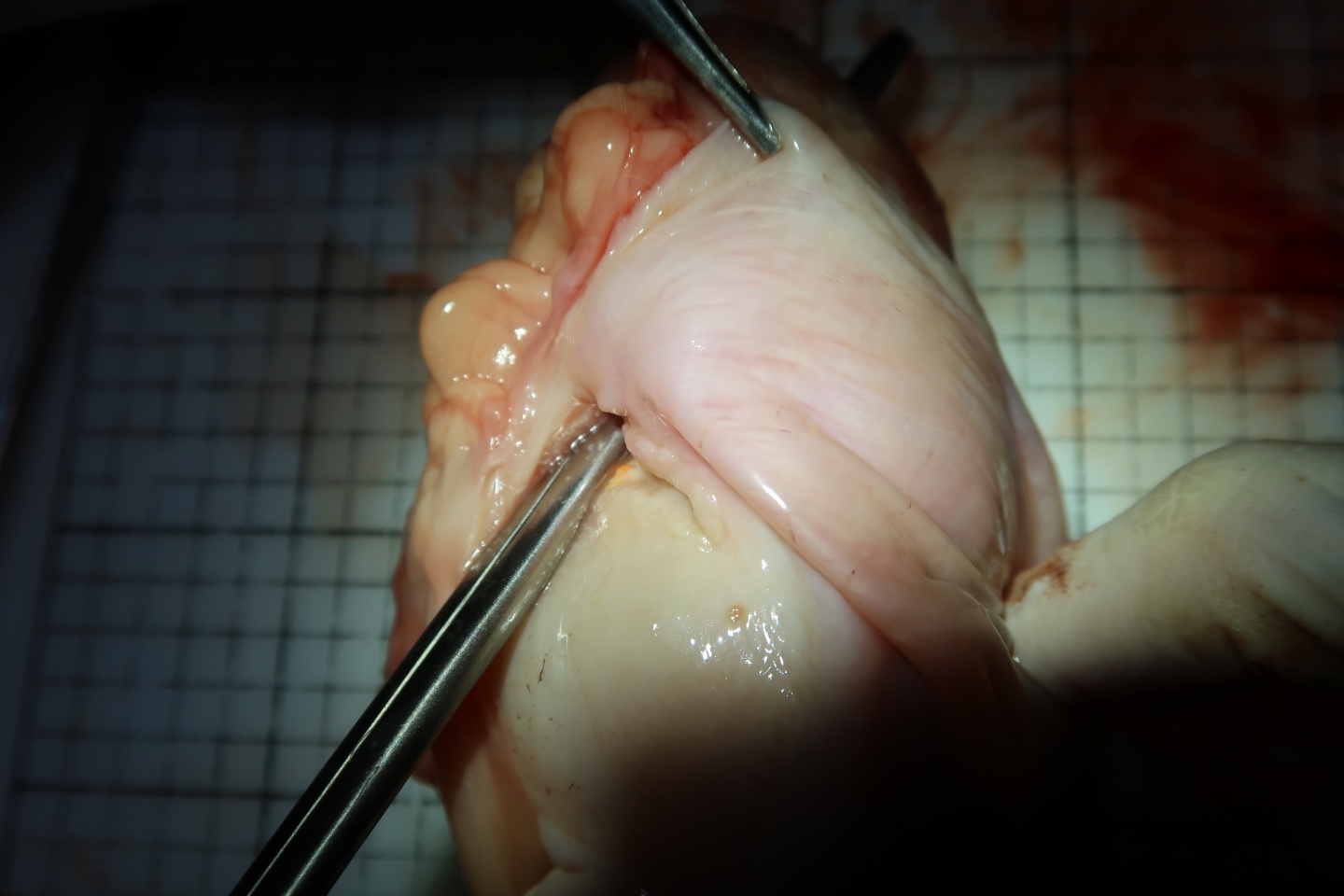

Supplement: Supplementary file 1 [file vetsci-12-01045-s001.zip › KakaoTalk_20221022_163714238_21.jpg]

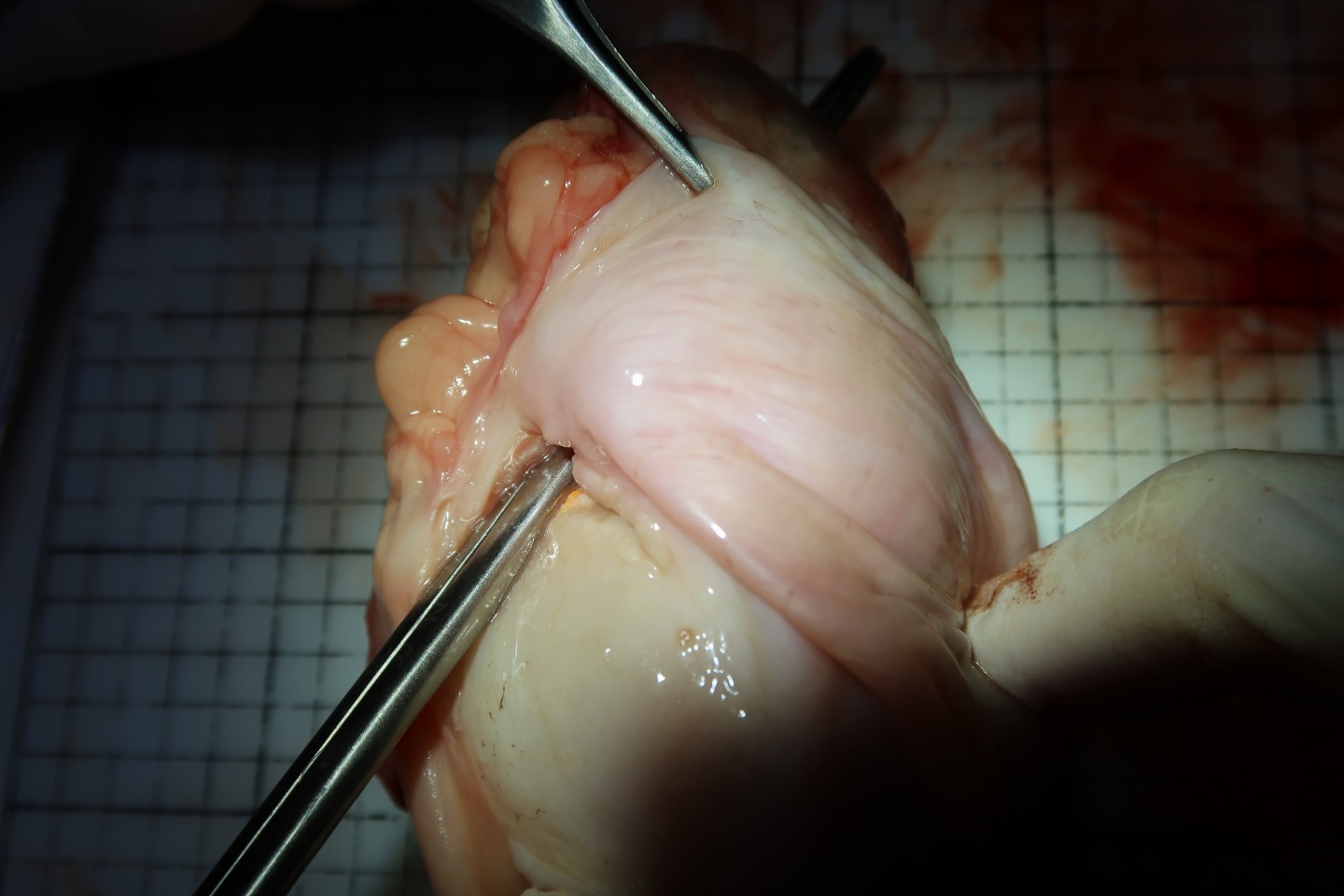

Supplement: Supplementary file 1 [file vetsci-12-01045-s001.zip › KakaoTalk_20221022_163714238_22.jpg]

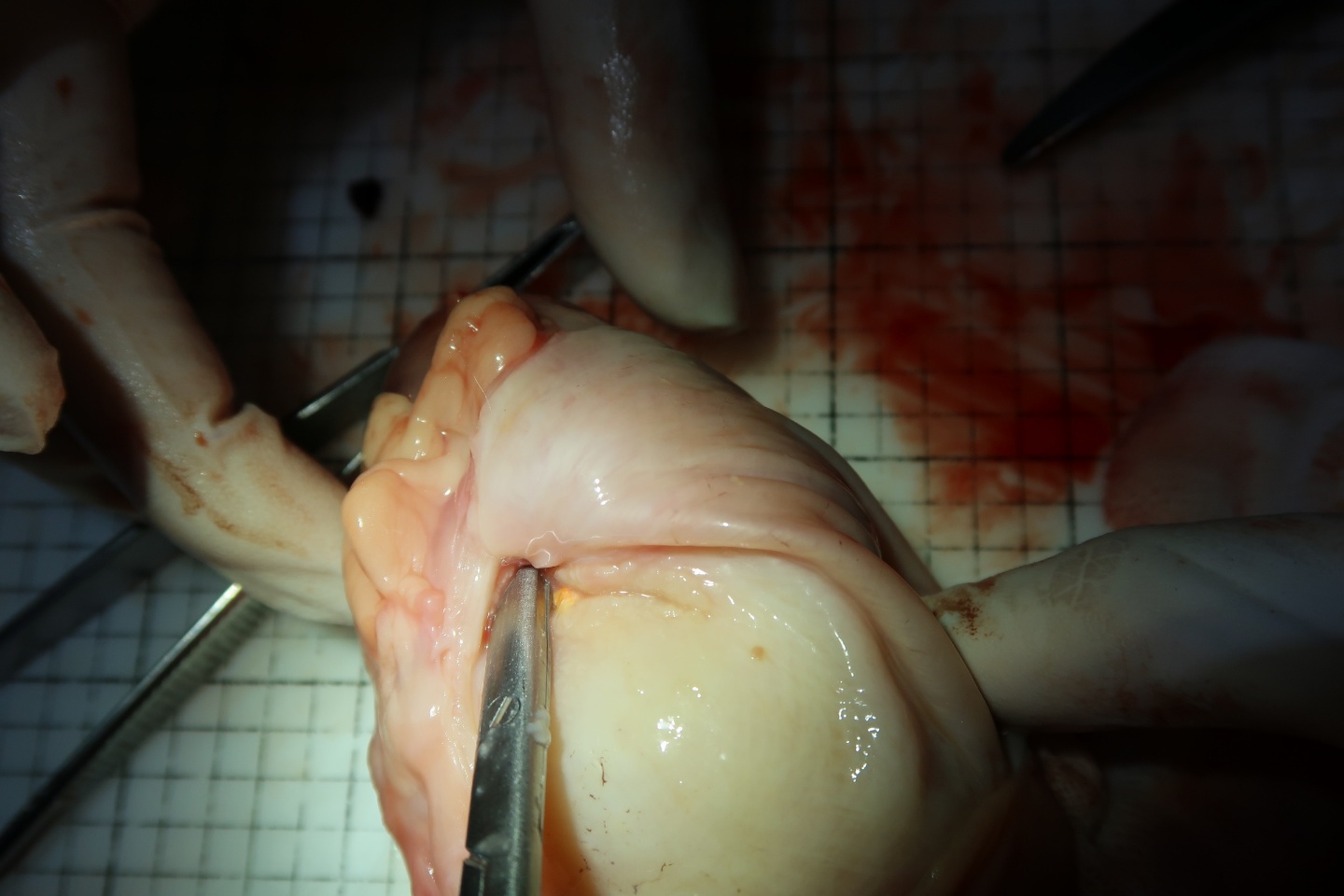

Supplement: Supplementary file 1 [file vetsci-12-01045-s001.zip › KakaoTalk_20221022_163714238_23.jpg]

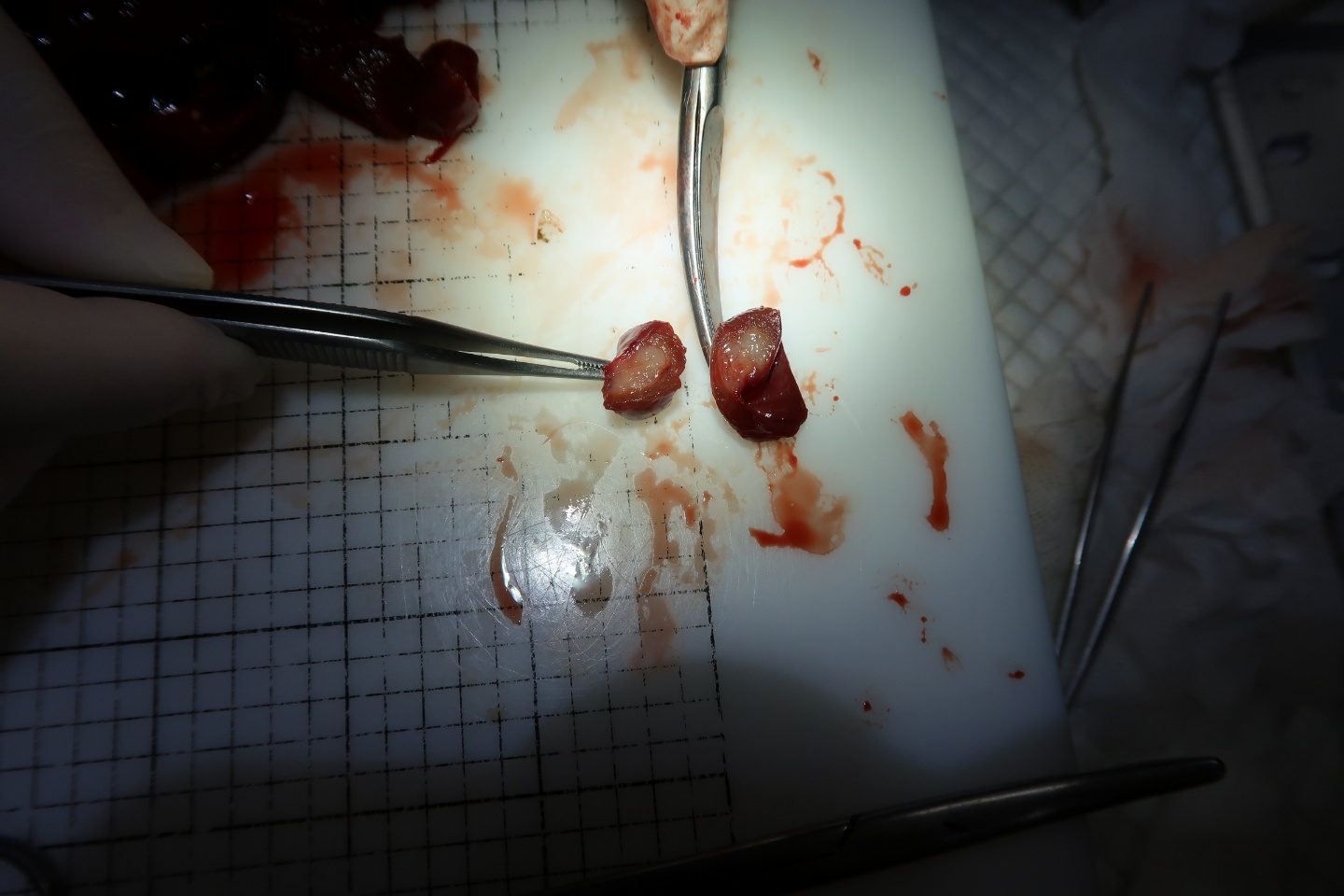

Supplement: Supplementary file 1 [file vetsci-12-01045-s001.zip › KakaoTalk_20221022_163714238_24.jpg]

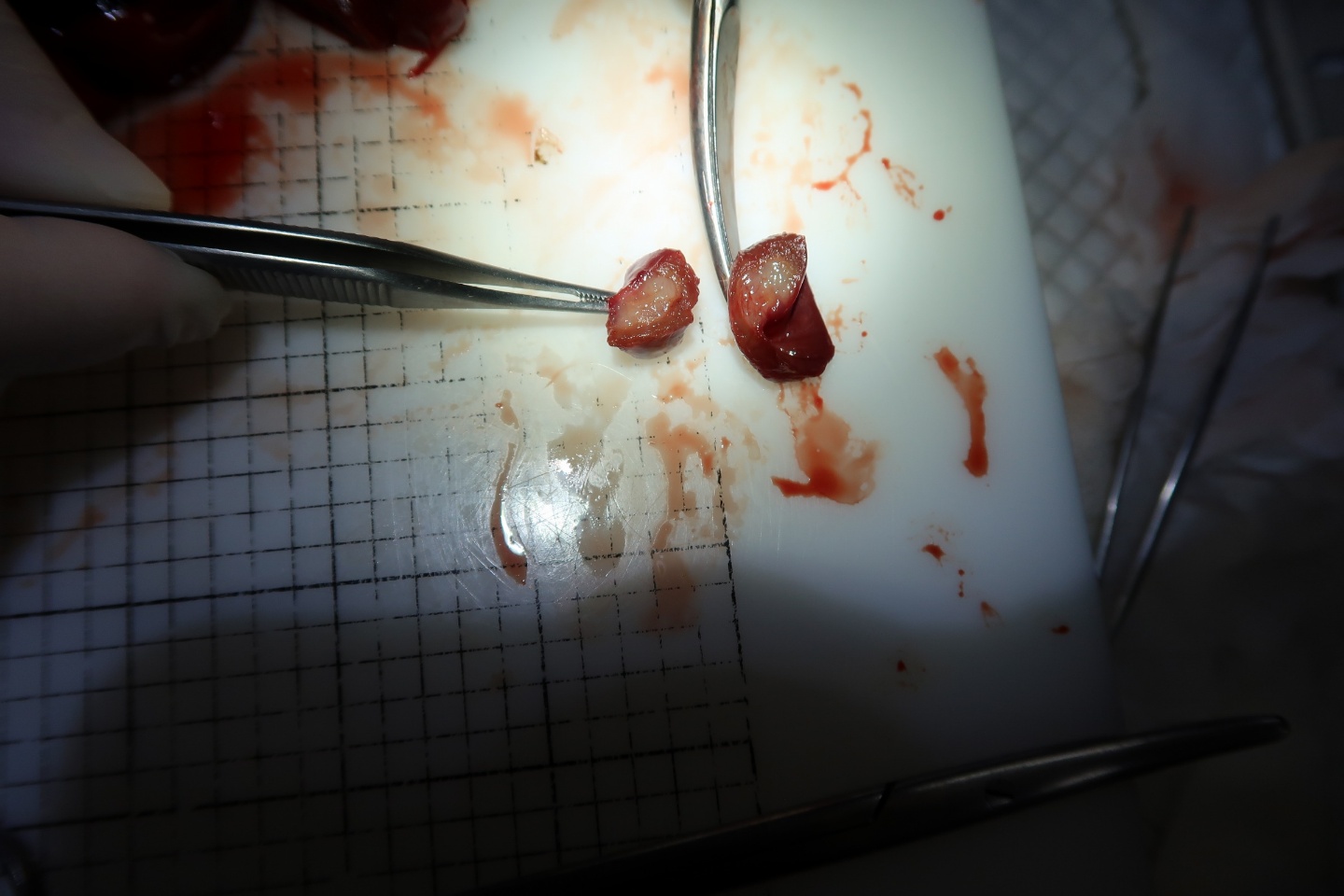

Supplement: Supplementary file 1 [file vetsci-12-01045-s001.zip › KakaoTalk_20221022_163714238_25.jpg]

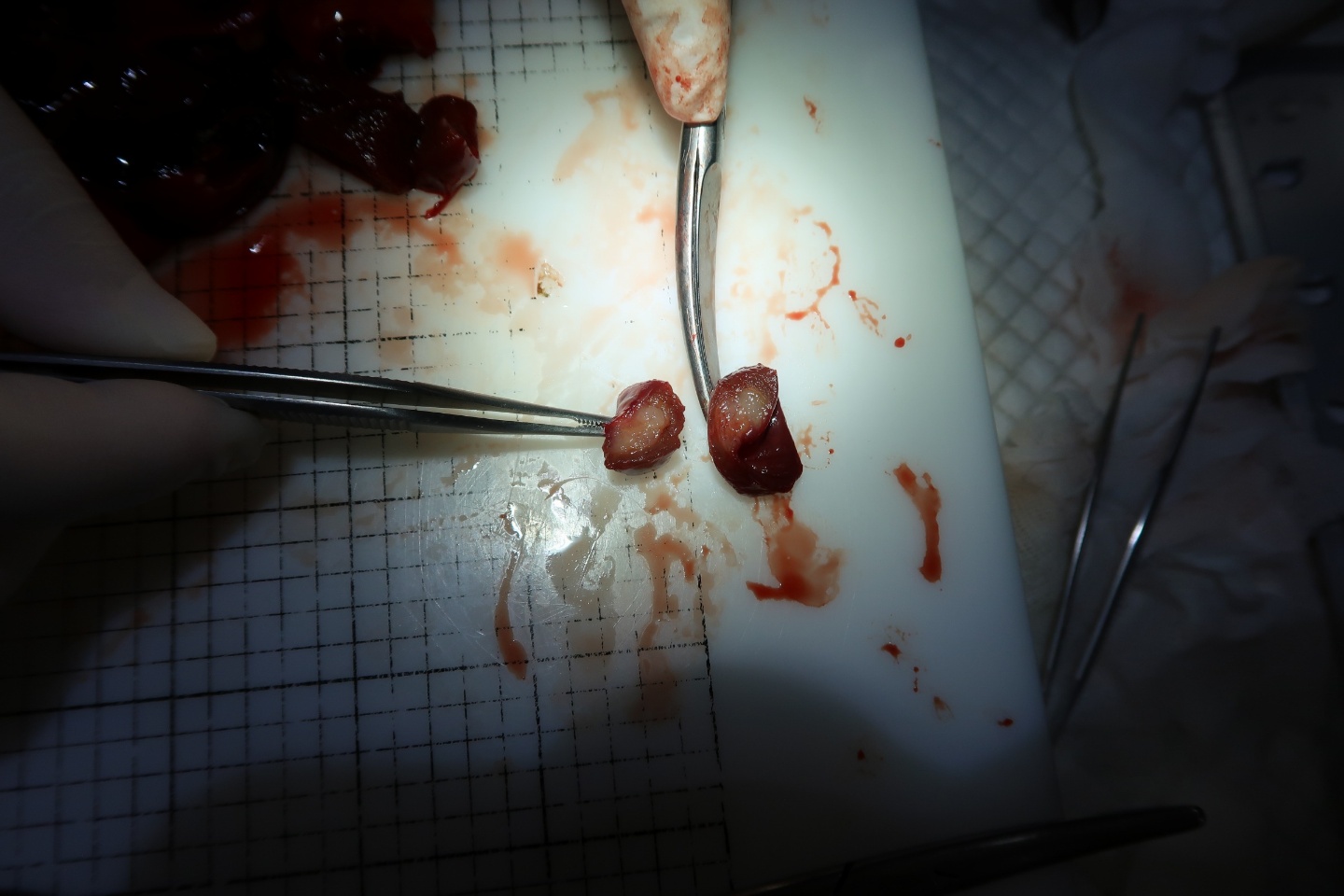

Supplement: Supplementary file 1 [file vetsci-12-01045-s001.zip › KakaoTalk_20221022_163714238_26.jpg]

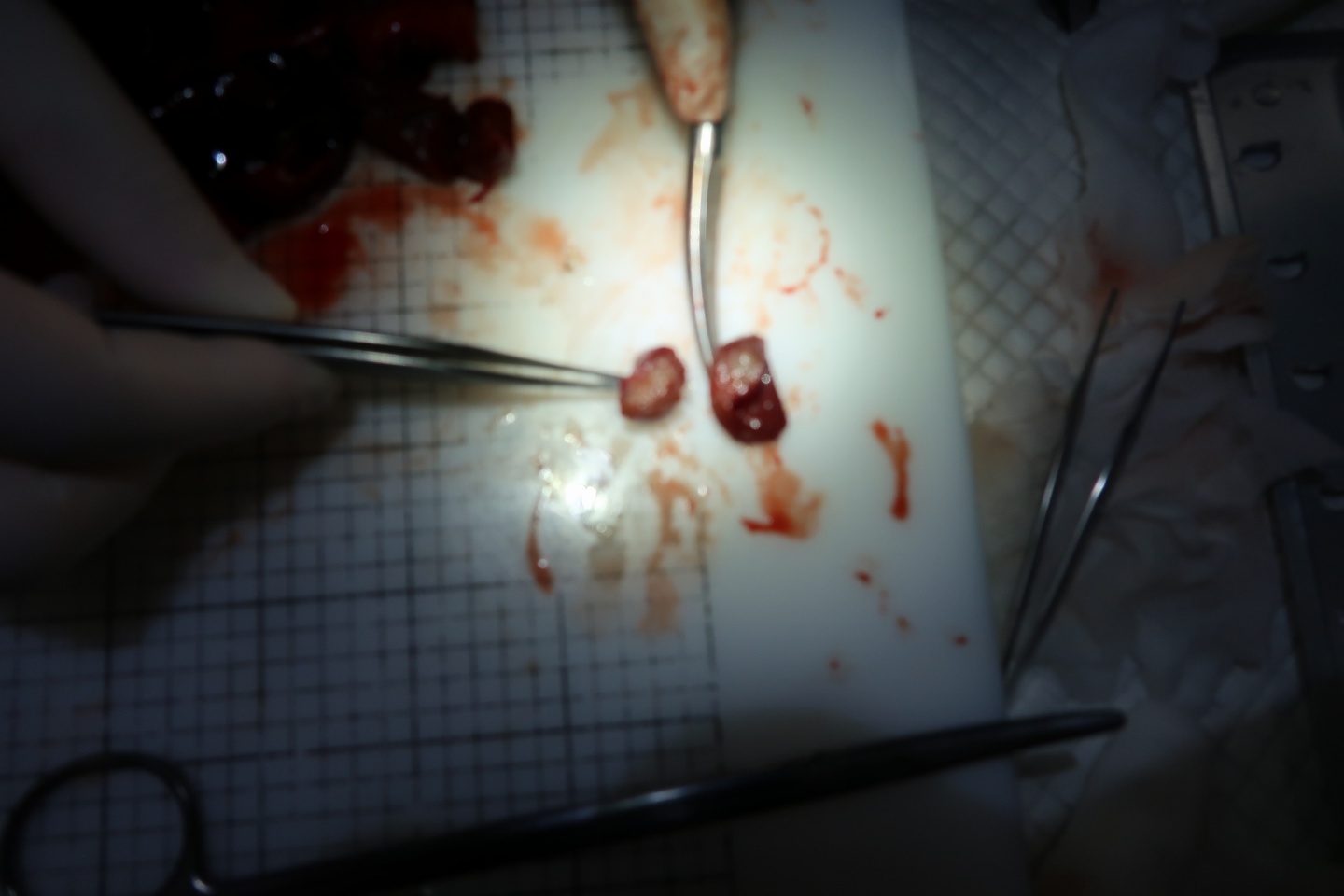

Supplement: Supplementary file 1 [file vetsci-12-01045-s001.zip › KakaoTalk_20221022_163714238_27.jpg]

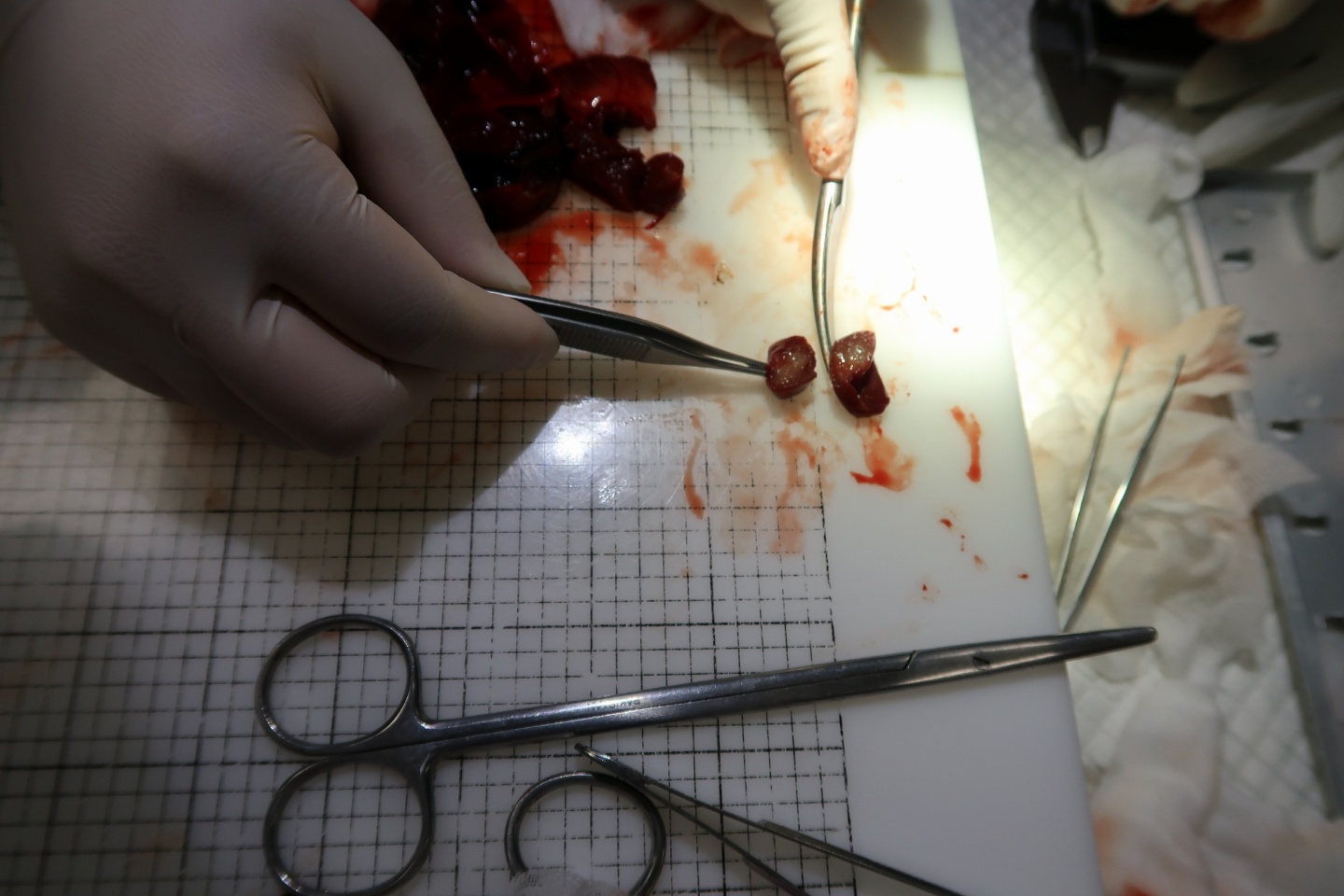

Supplement: Supplementary file 1 [file vetsci-12-01045-s001.zip › KakaoTalk_20221022_163714238_28.jpg]

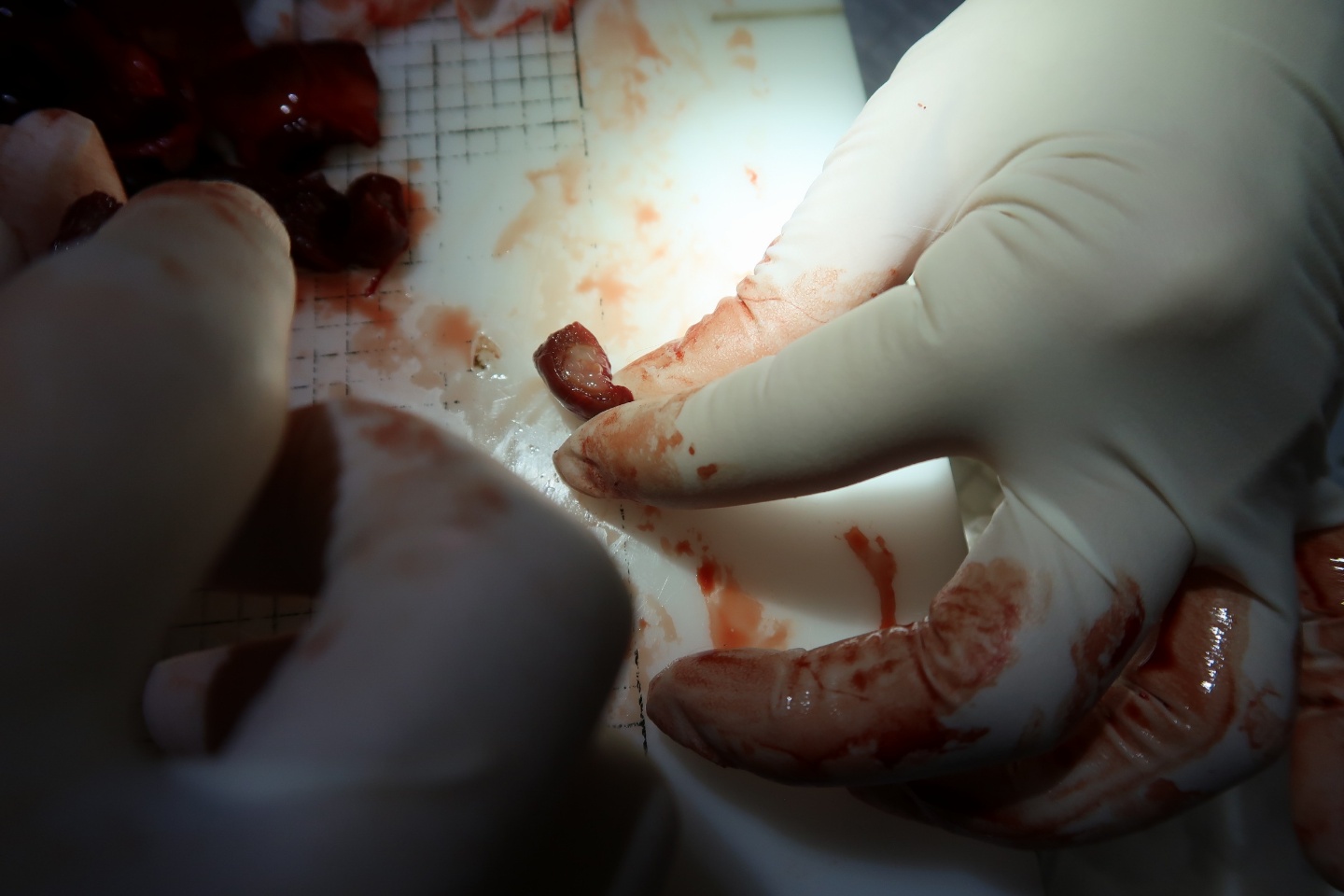

Supplement: Supplementary file 1 [file vetsci-12-01045-s001.zip › KakaoTalk_20221022_163714238_29.jpg]

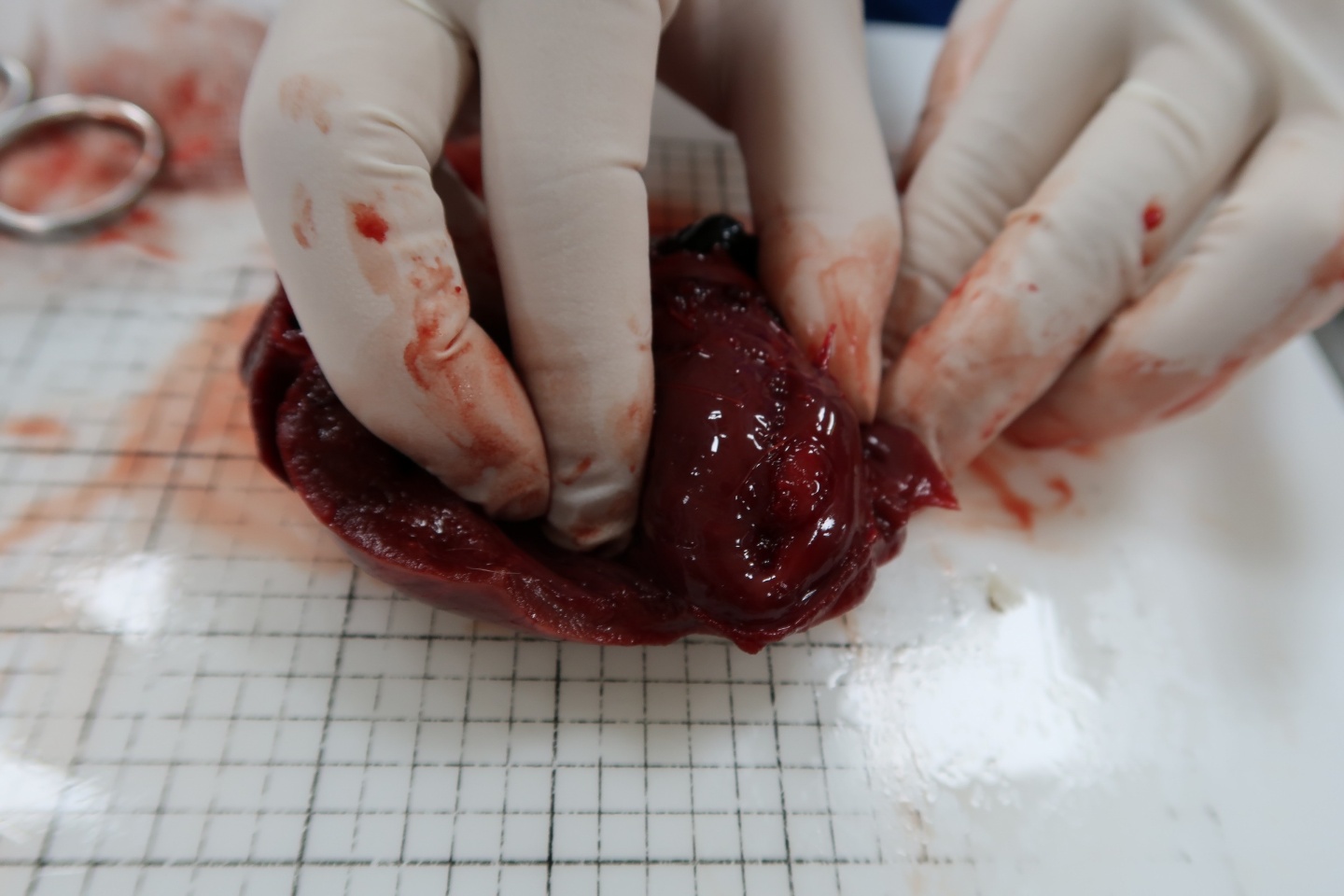

Supplement: Supplementary file 1 [file vetsci-12-01045-s001.zip › KakaoTalk_20221022_163804746_01.jpg]

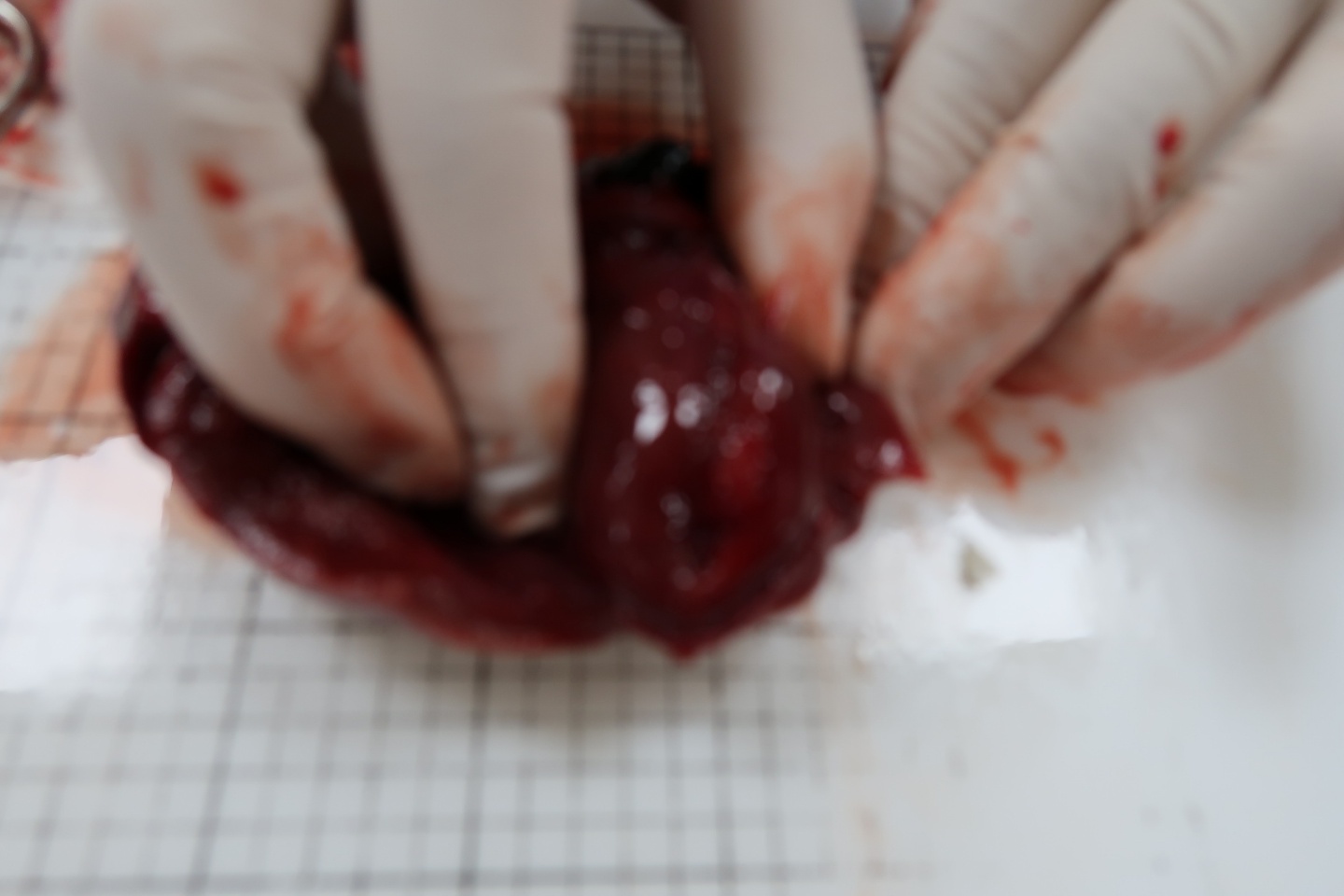

Supplement: Supplementary file 1 [file vetsci-12-01045-s001.zip › KakaoTalk_20221022_163804746_02.jpg]

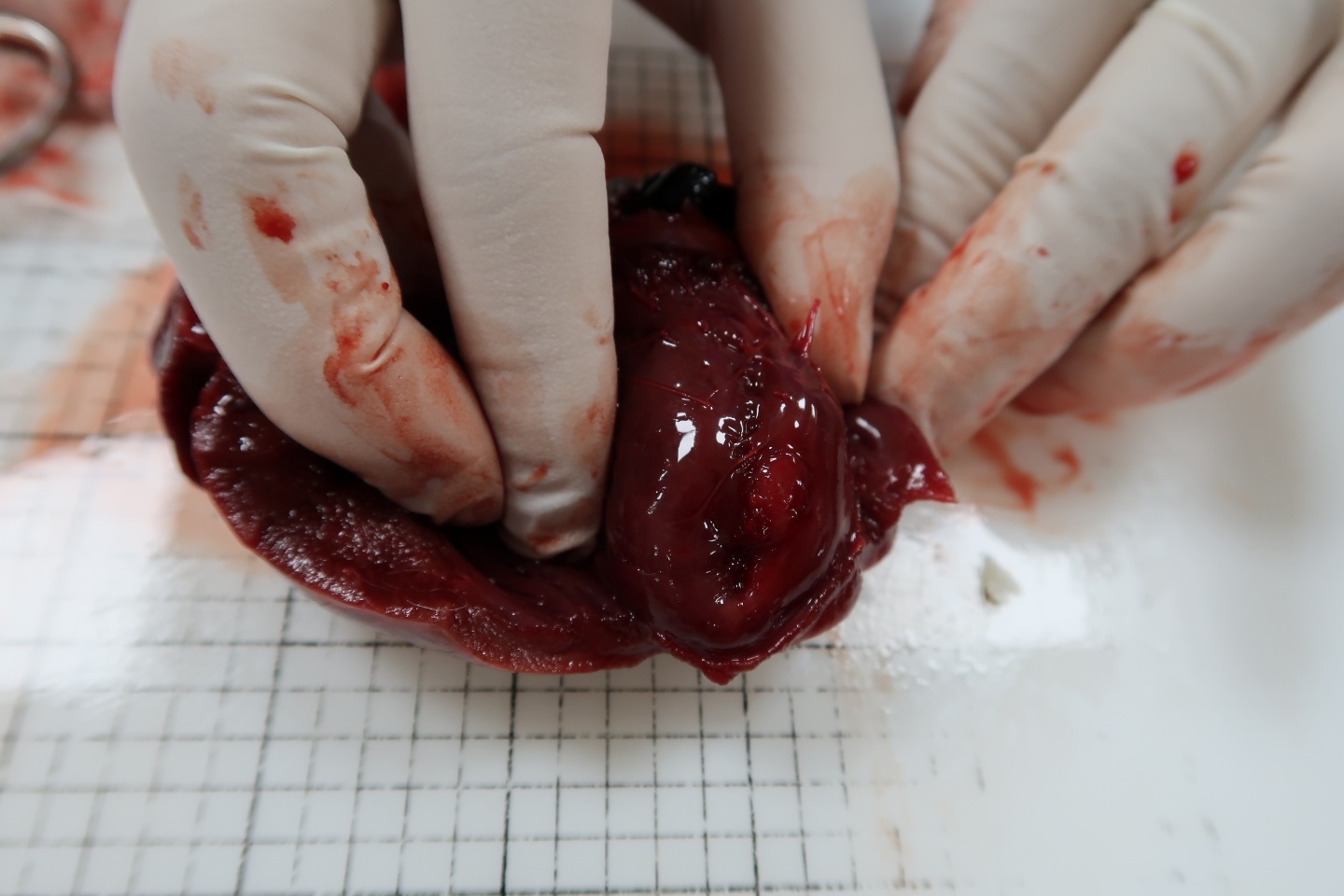

Supplement: Supplementary file 1 [file vetsci-12-01045-s001.zip › KakaoTalk_20221022_163804746_03.jpg]

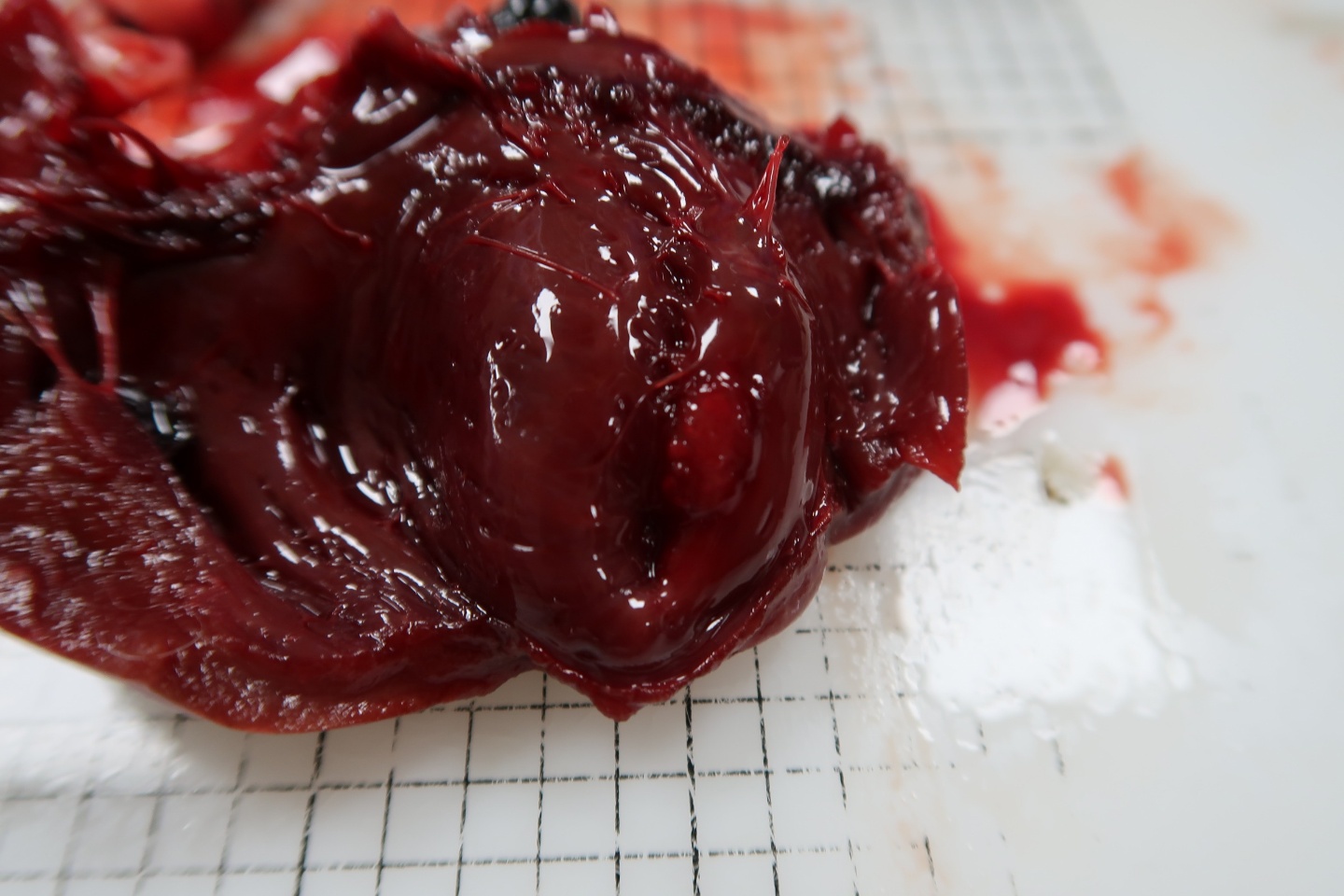

Supplement: Supplementary file 1 [file vetsci-12-01045-s001.zip › KakaoTalk_20221022_163804746_04.jpg]

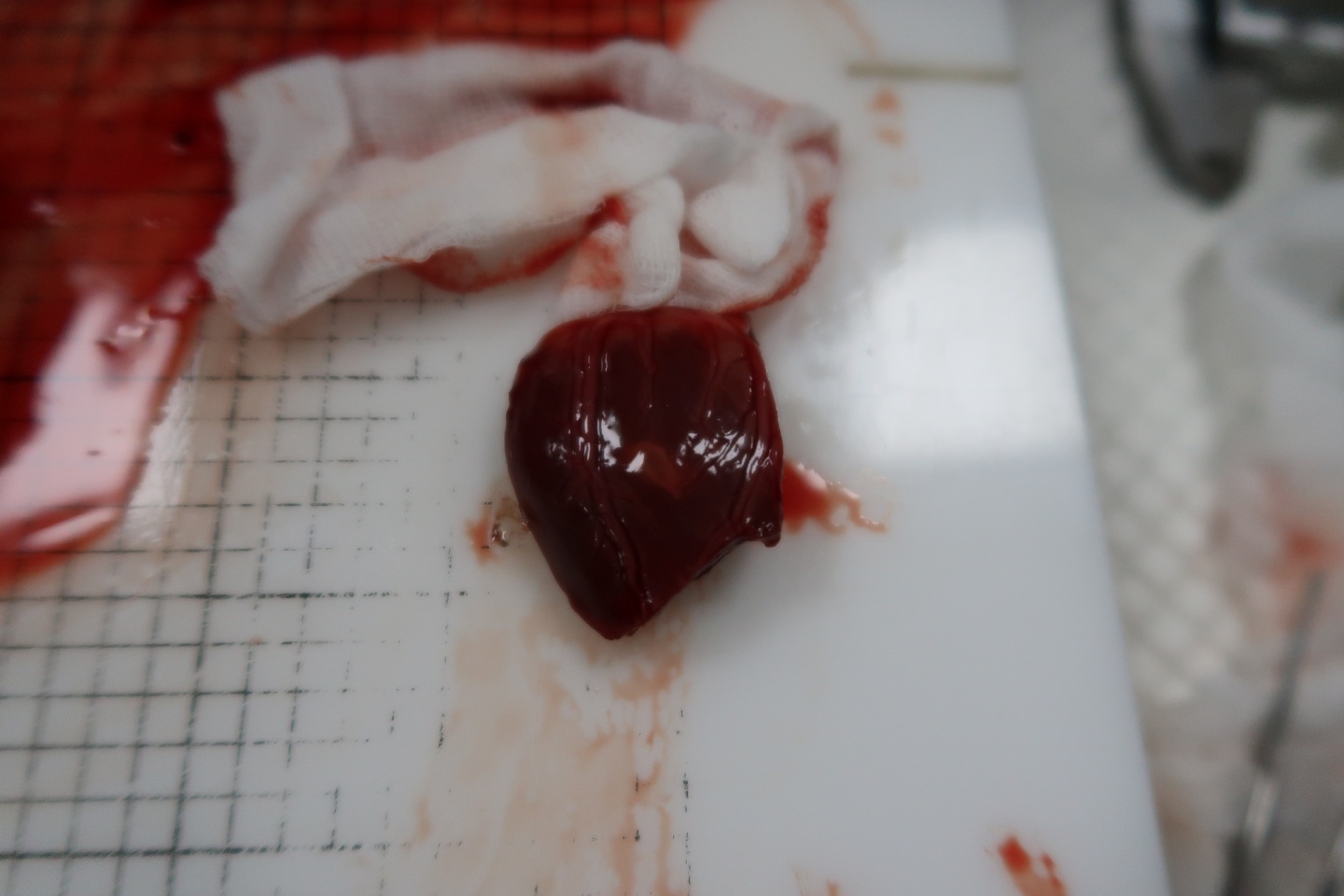

Supplement: Supplementary file 1 [file vetsci-12-01045-s001.zip › KakaoTalk_20221022_163804746_05.jpg]

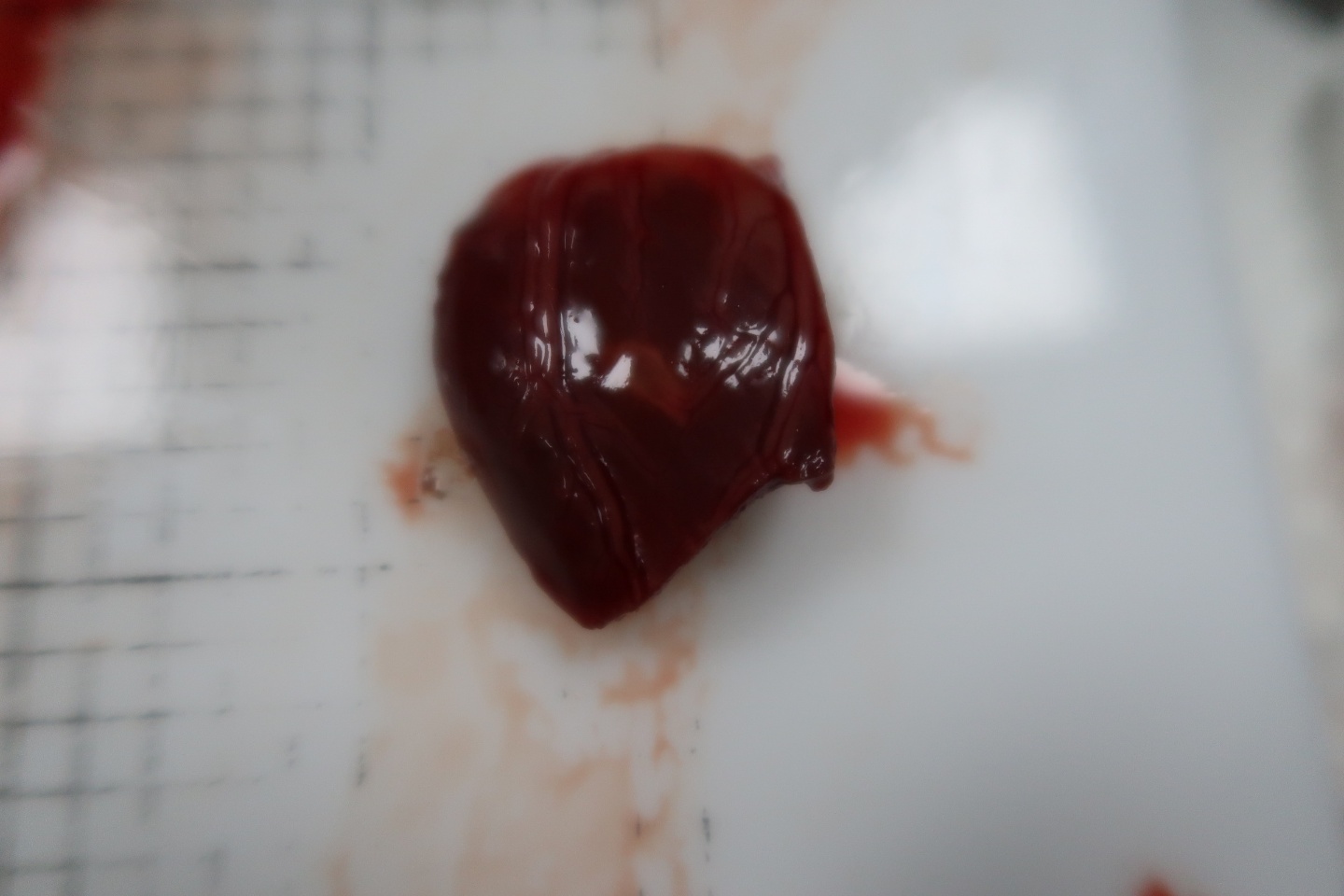

Supplement: Supplementary file 1 [file vetsci-12-01045-s001.zip › KakaoTalk_20221022_163804746_06.jpg]

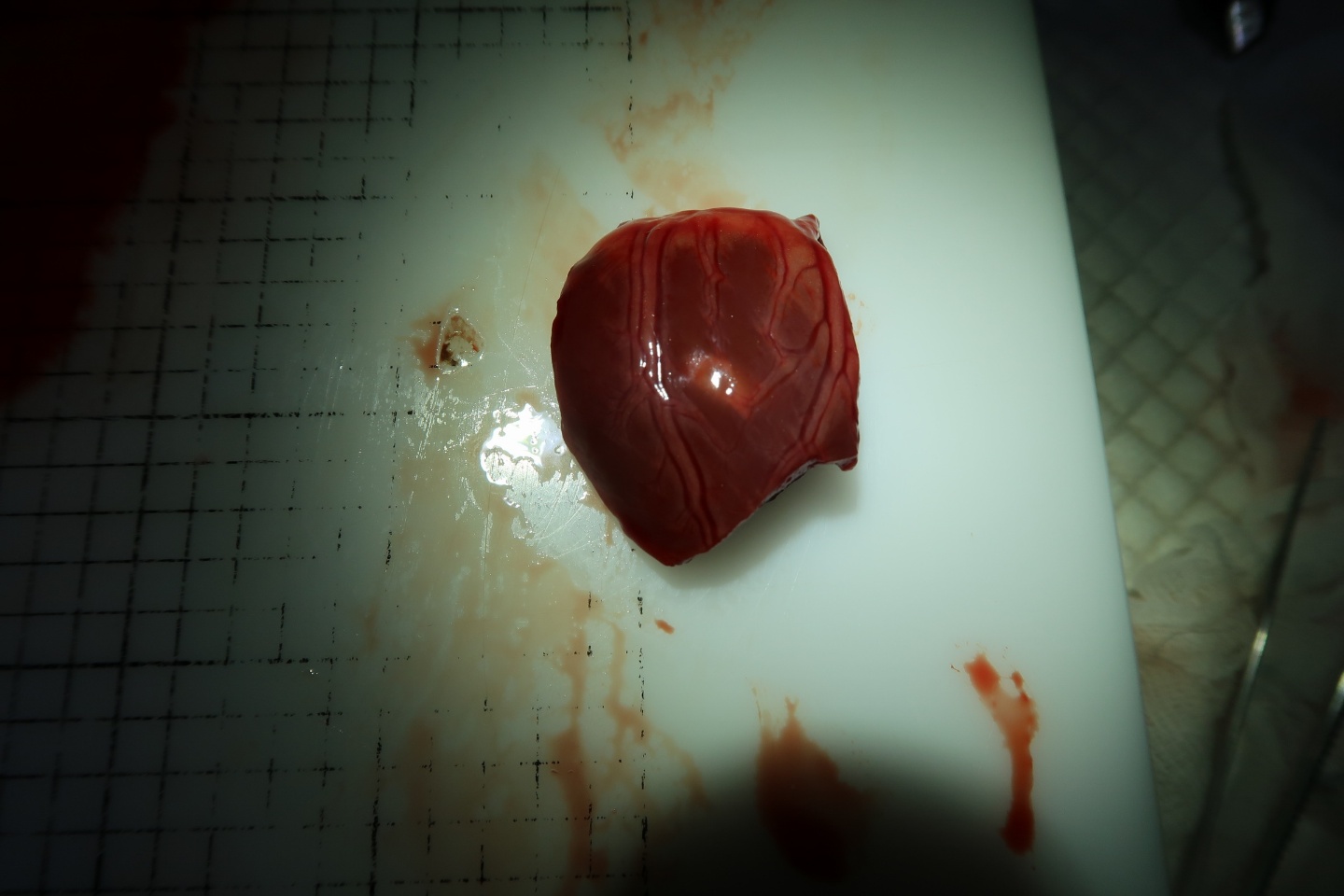

Supplement: Supplementary file 1 [file vetsci-12-01045-s001.zip › KakaoTalk_20221022_163804746_07.jpg]

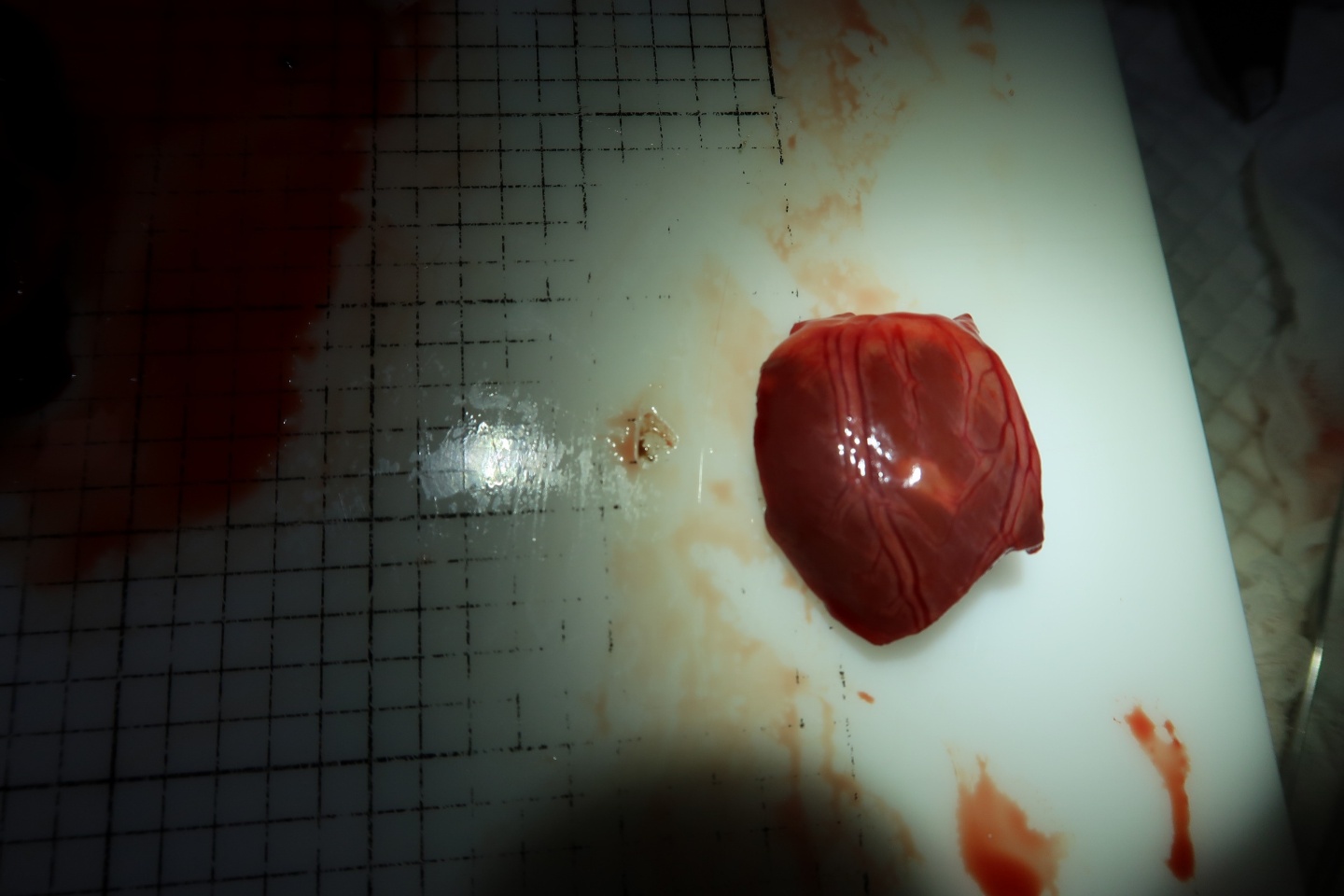

Supplement: Supplementary file 1 [file vetsci-12-01045-s001.zip › KakaoTalk_20221022_163804746_08.jpg]

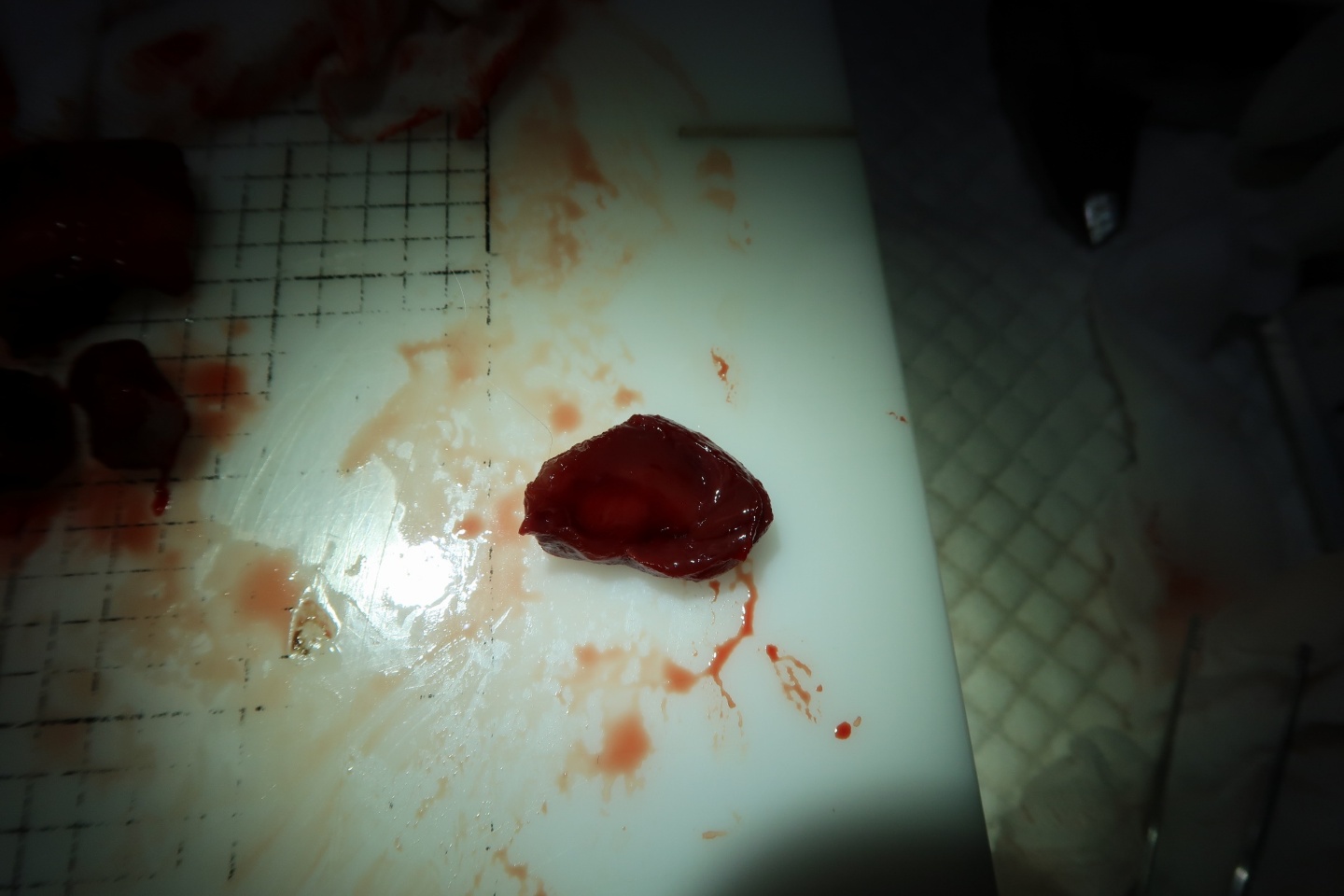

Supplement: Supplementary file 1 [file vetsci-12-01045-s001.zip › KakaoTalk_20221022_163804746_09.jpg]

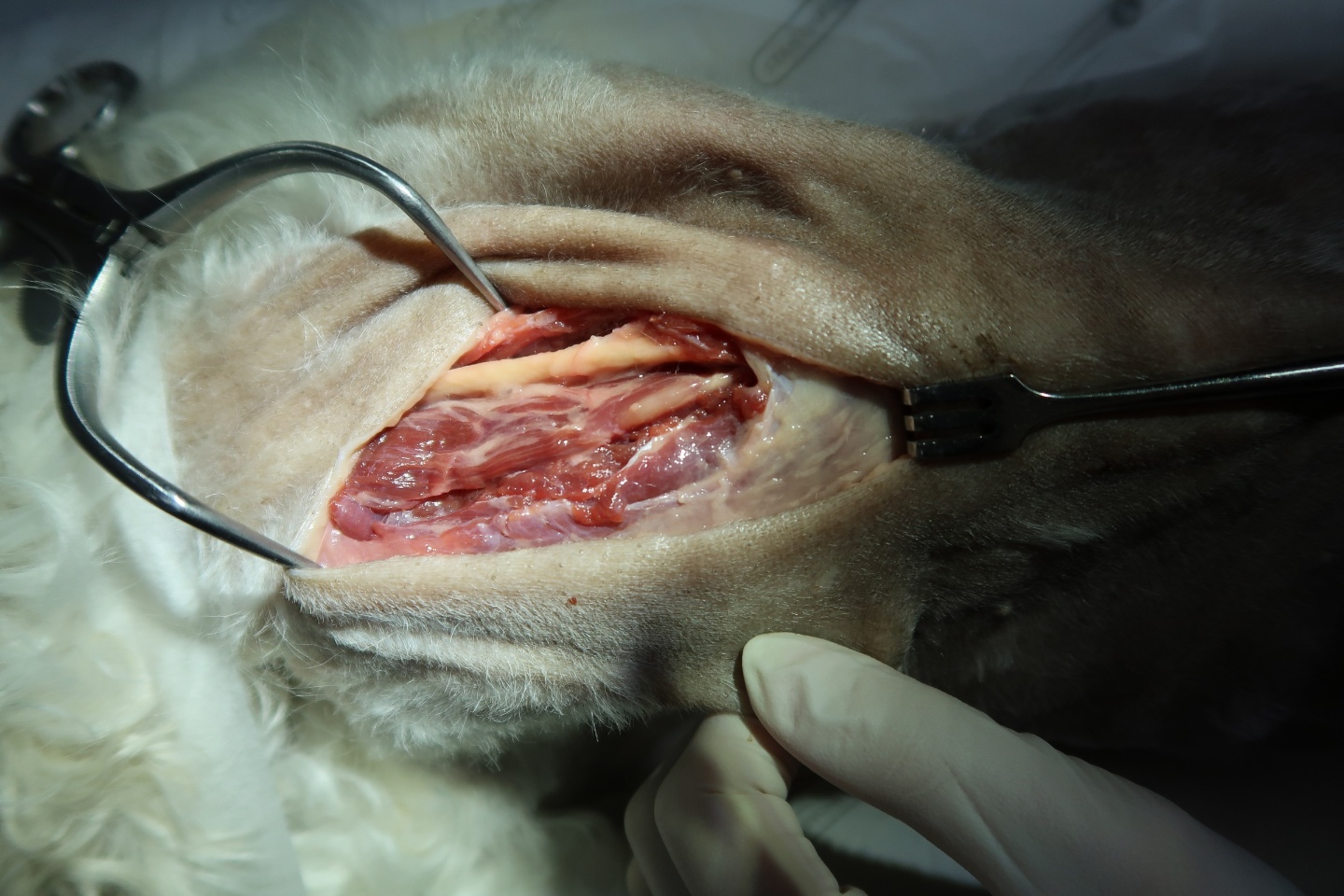

Supplement: Supplementary file 1 [file vetsci-12-01045-s001.zip › KakaoTalk_20221022_164754286.jpg]

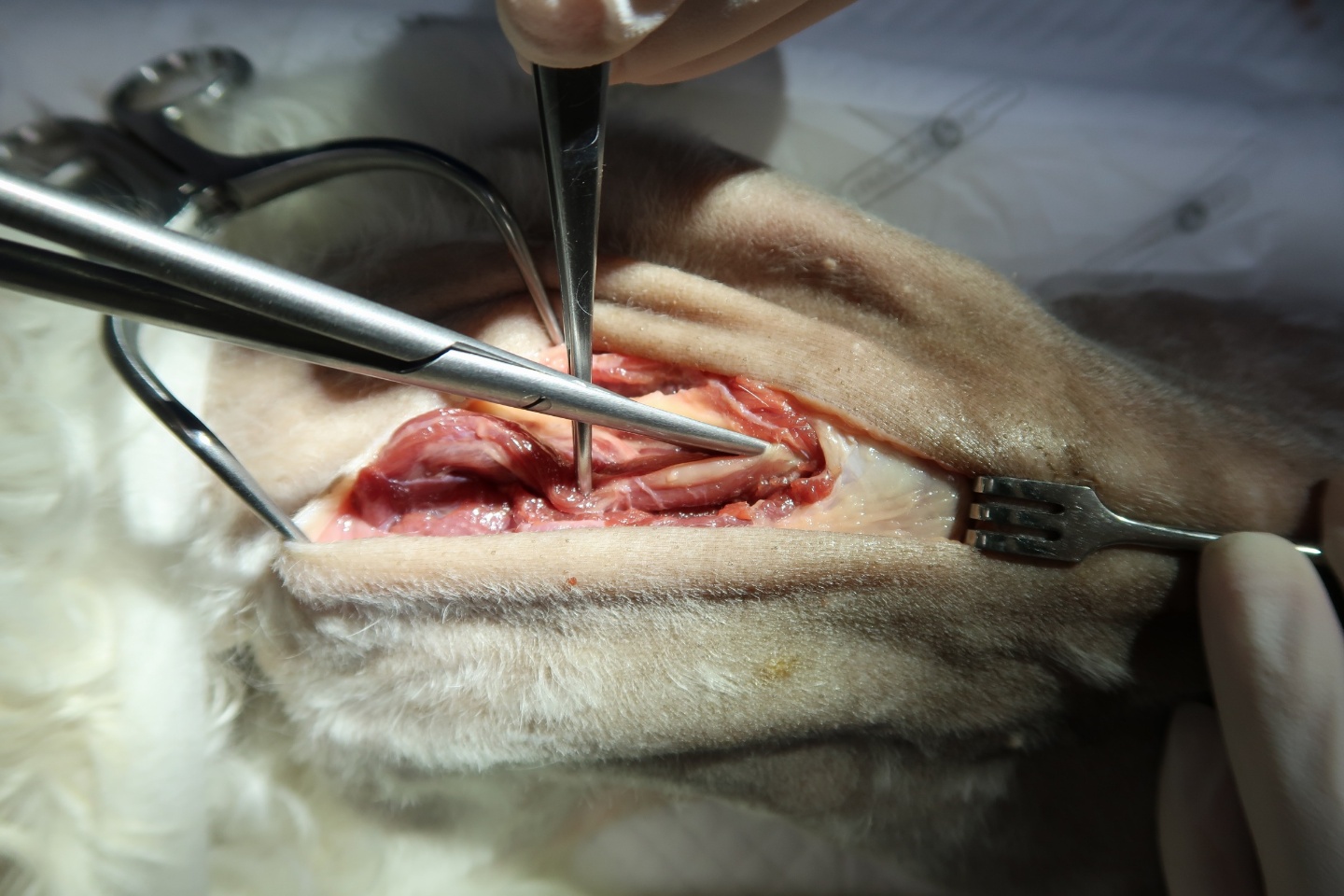

Supplement: Supplementary file 1 [file vetsci-12-01045-s001.zip › KakaoTalk_20221022_164754286_01.jpg]

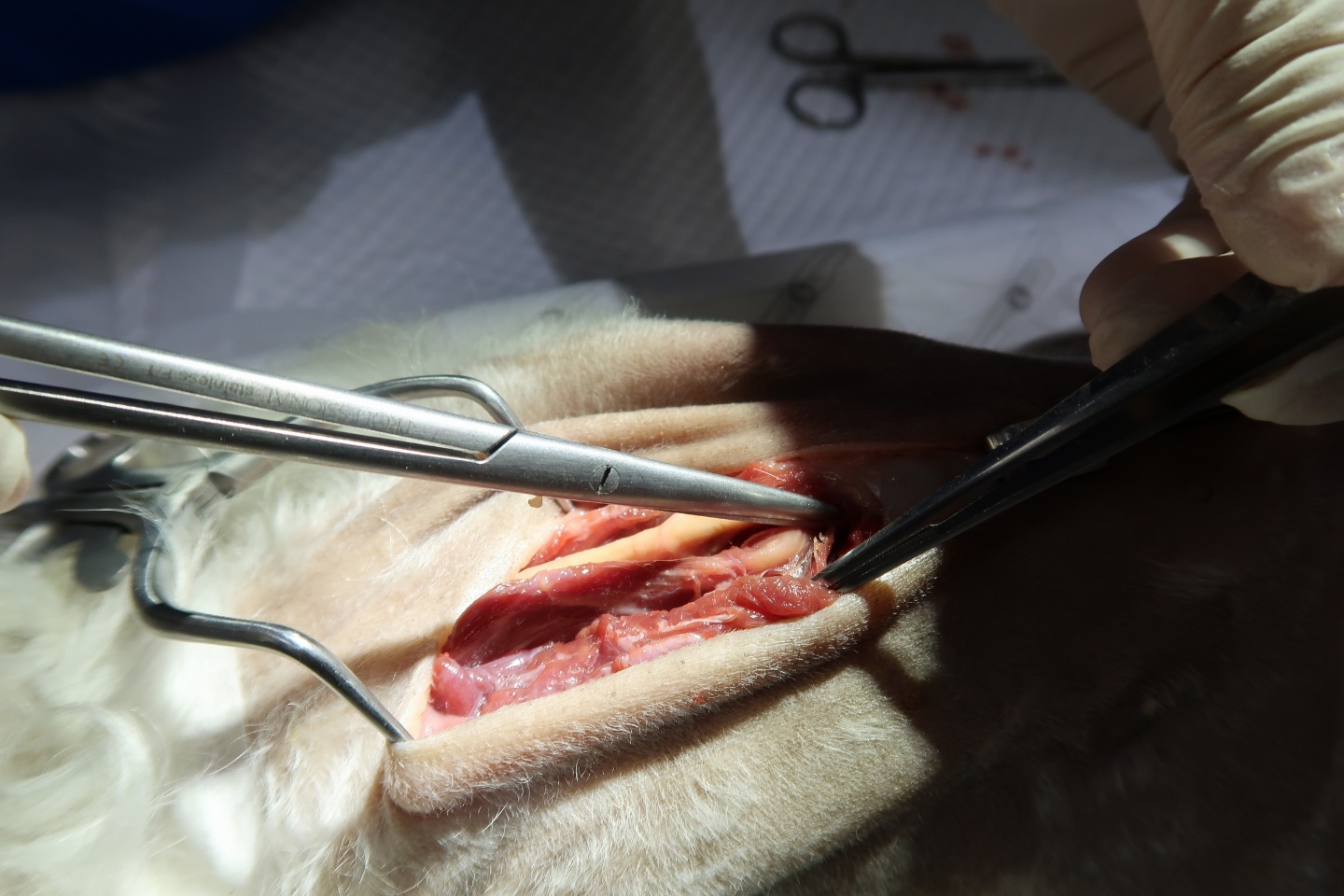

Supplement: Supplementary file 1 [file vetsci-12-01045-s001.zip › KakaoTalk_20221022_164754286_02.jpg]

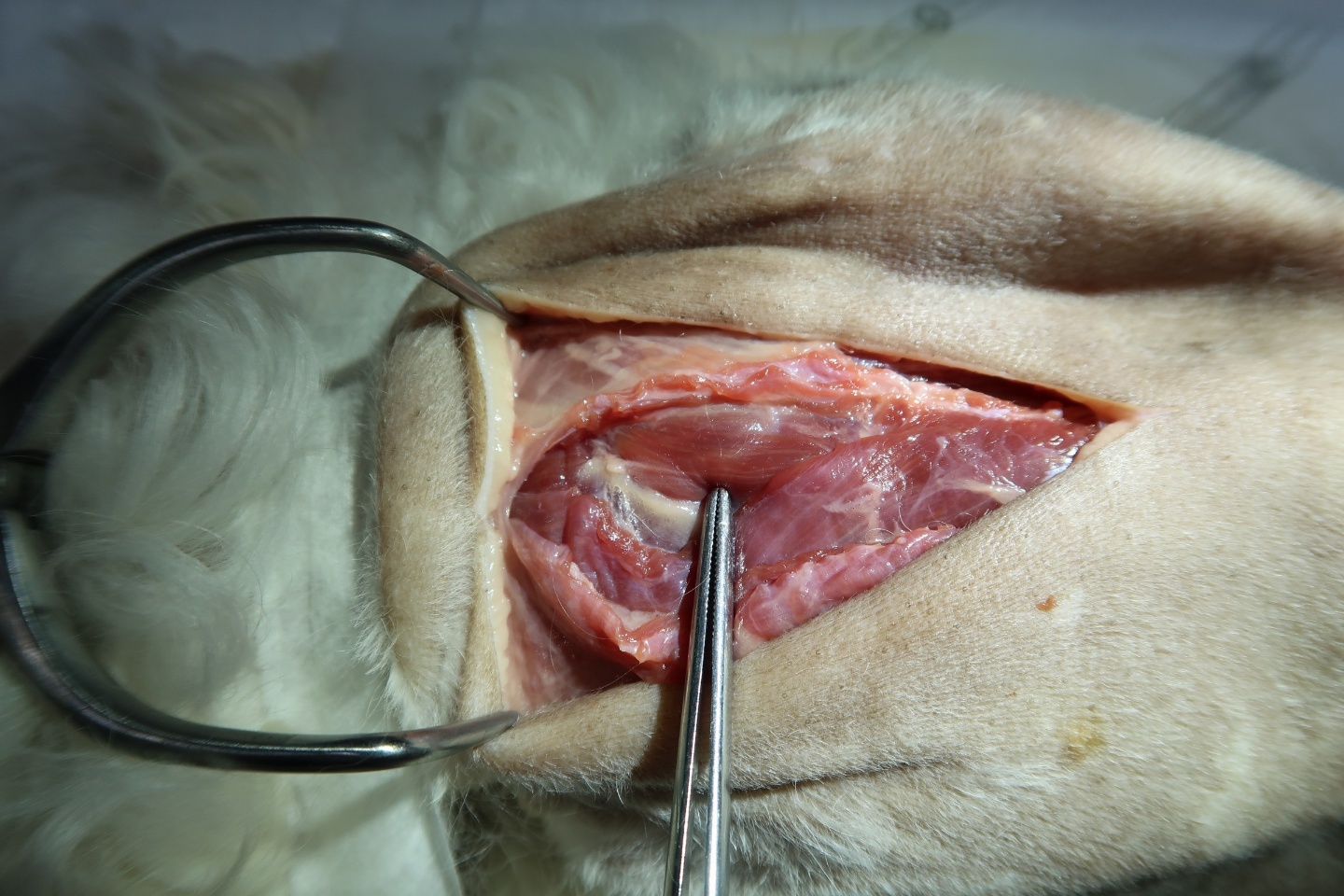

Supplement: Supplementary file 1 [file vetsci-12-01045-s001.zip › KakaoTalk_20221022_164754286_03.jpg]

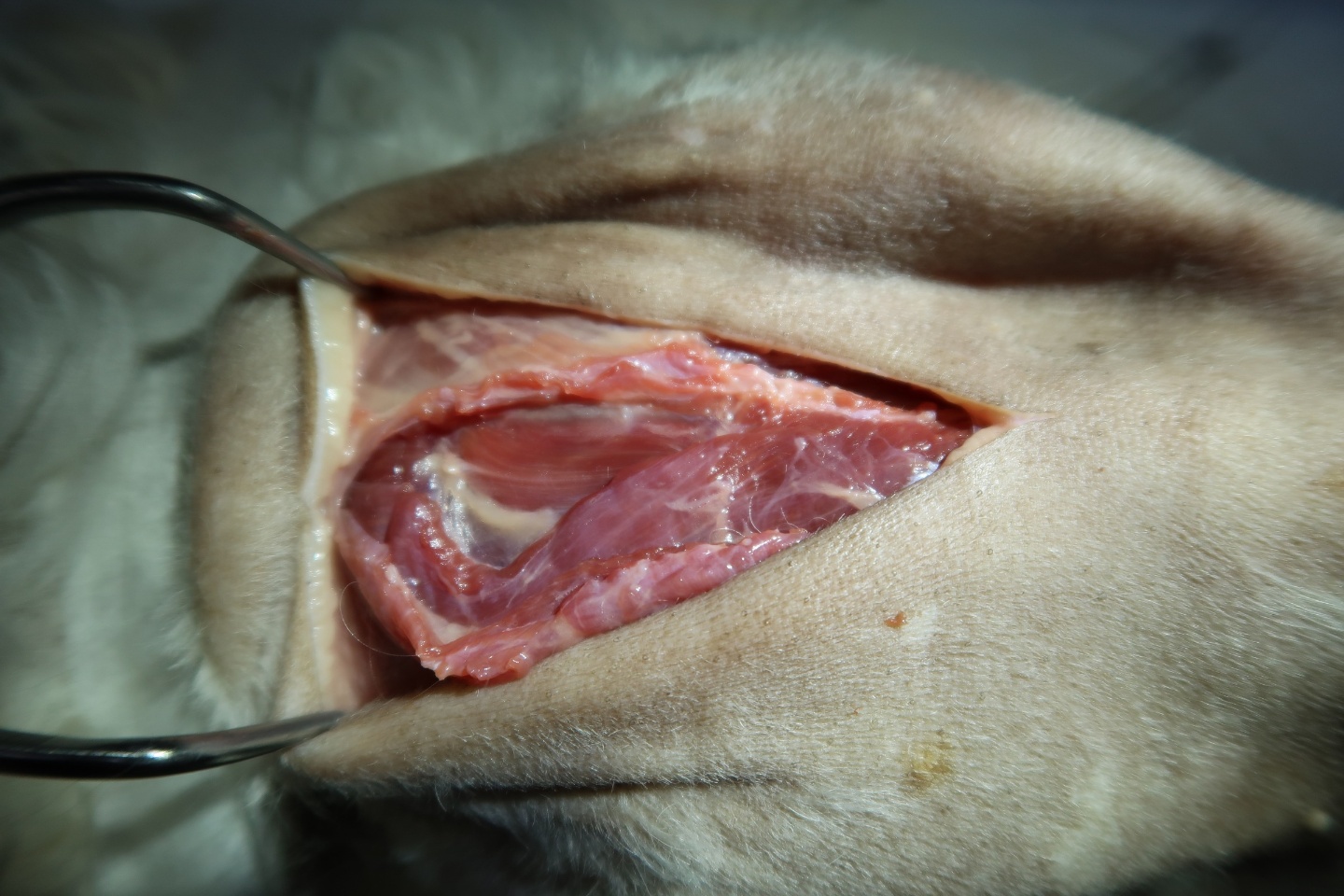

Supplement: Supplementary file 1 [file vetsci-12-01045-s001.zip › KakaoTalk_20221022_164754286_04.jpg]

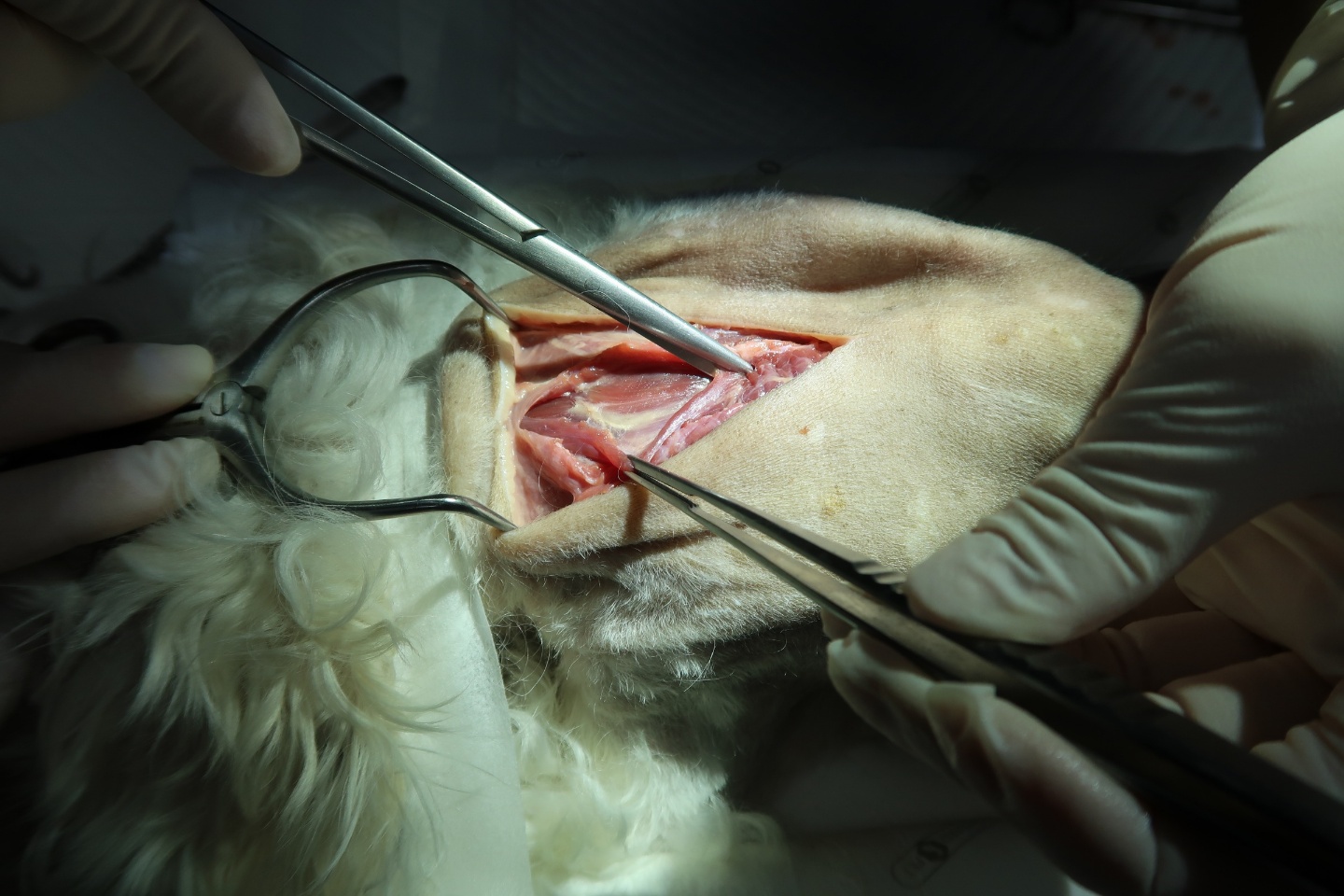

Supplement: Supplementary file 1 [file vetsci-12-01045-s001.zip › KakaoTalk_20221022_164754286_05.jpg]

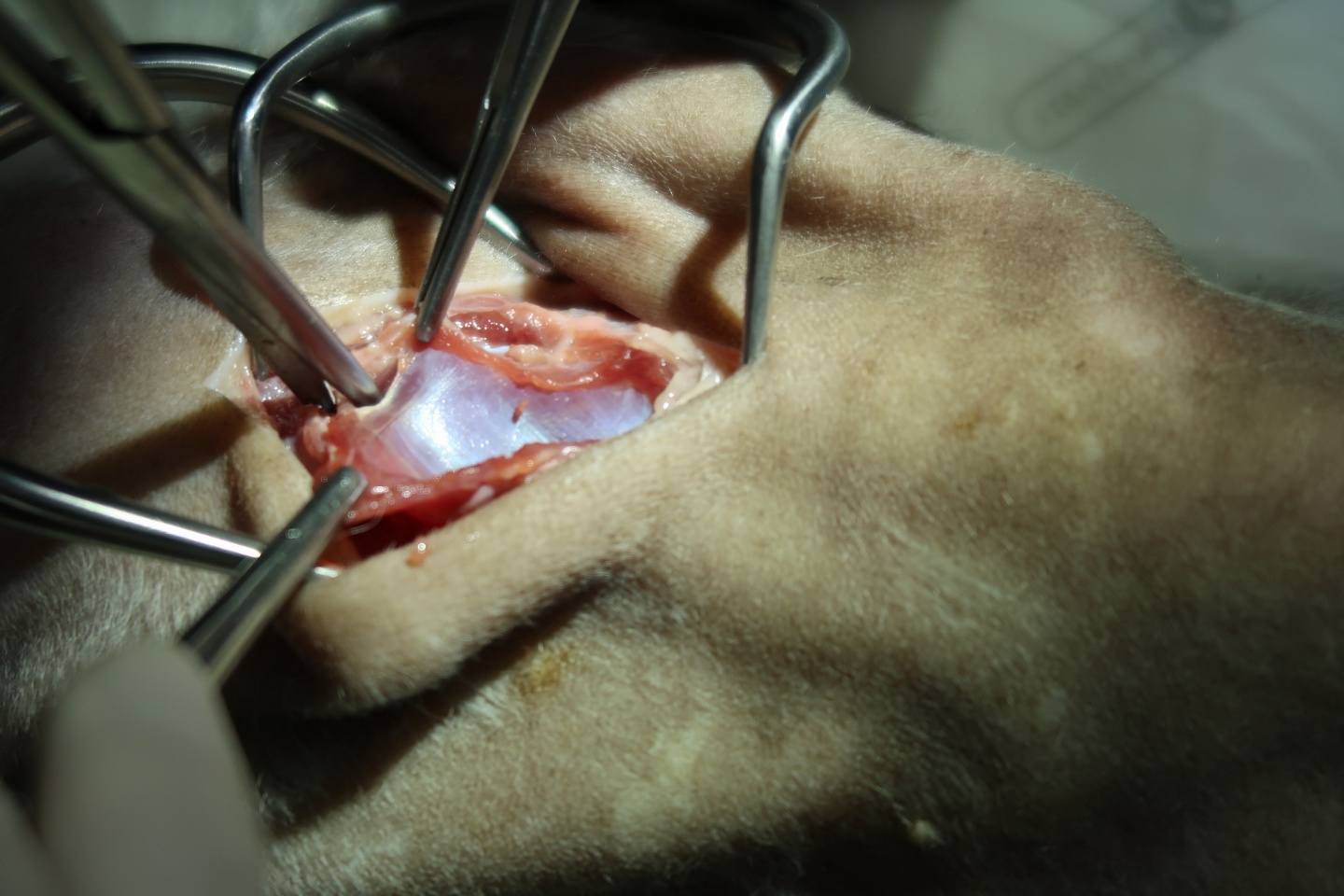

Supplement: Supplementary file 1 [file vetsci-12-01045-s001.zip › KakaoTalk_20221022_164754286_06.jpg]

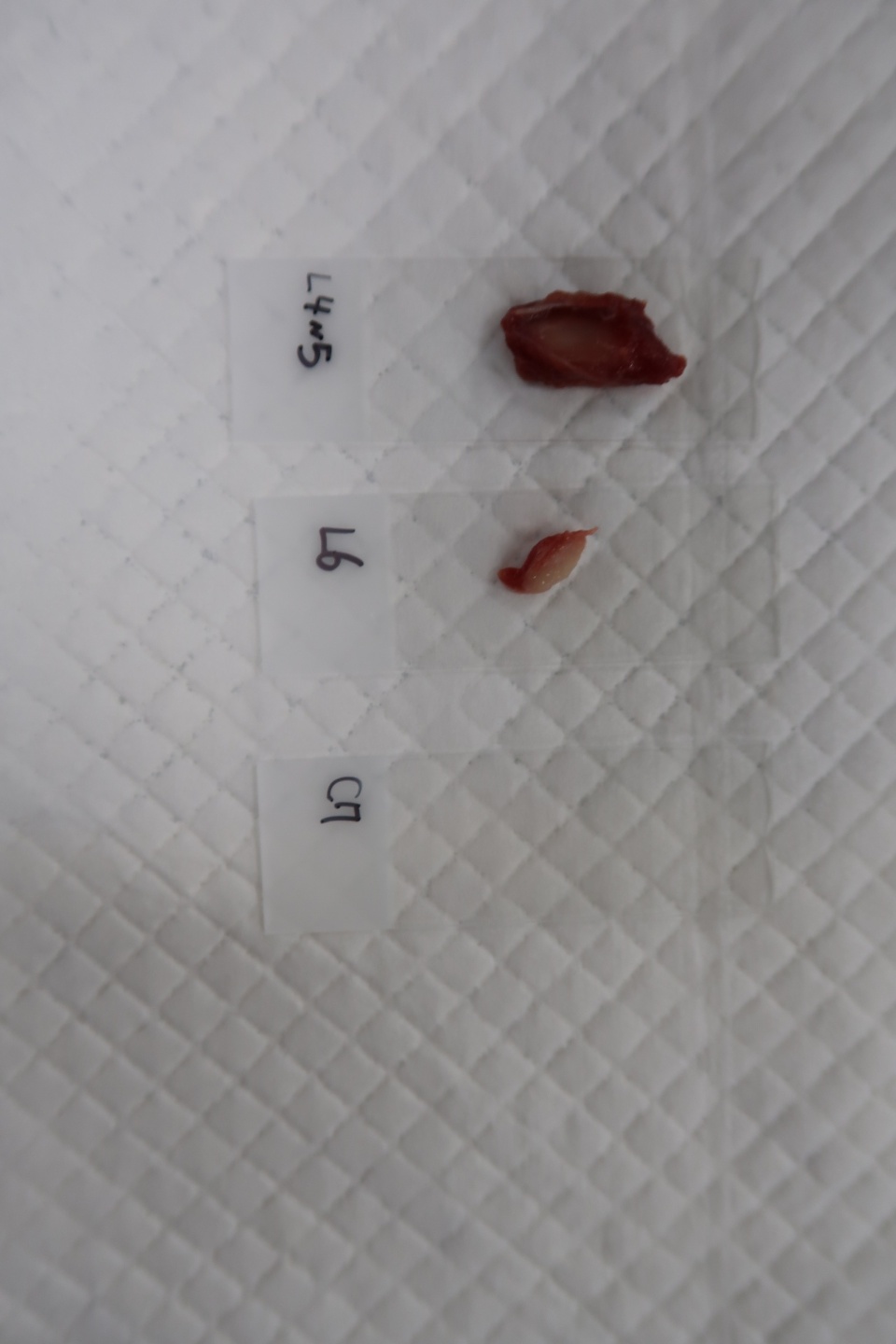

Supplement: Supplementary file 1 [file vetsci-12-01045-s001.zip › KakaoTalk_20221022_164754286_07.jpg]

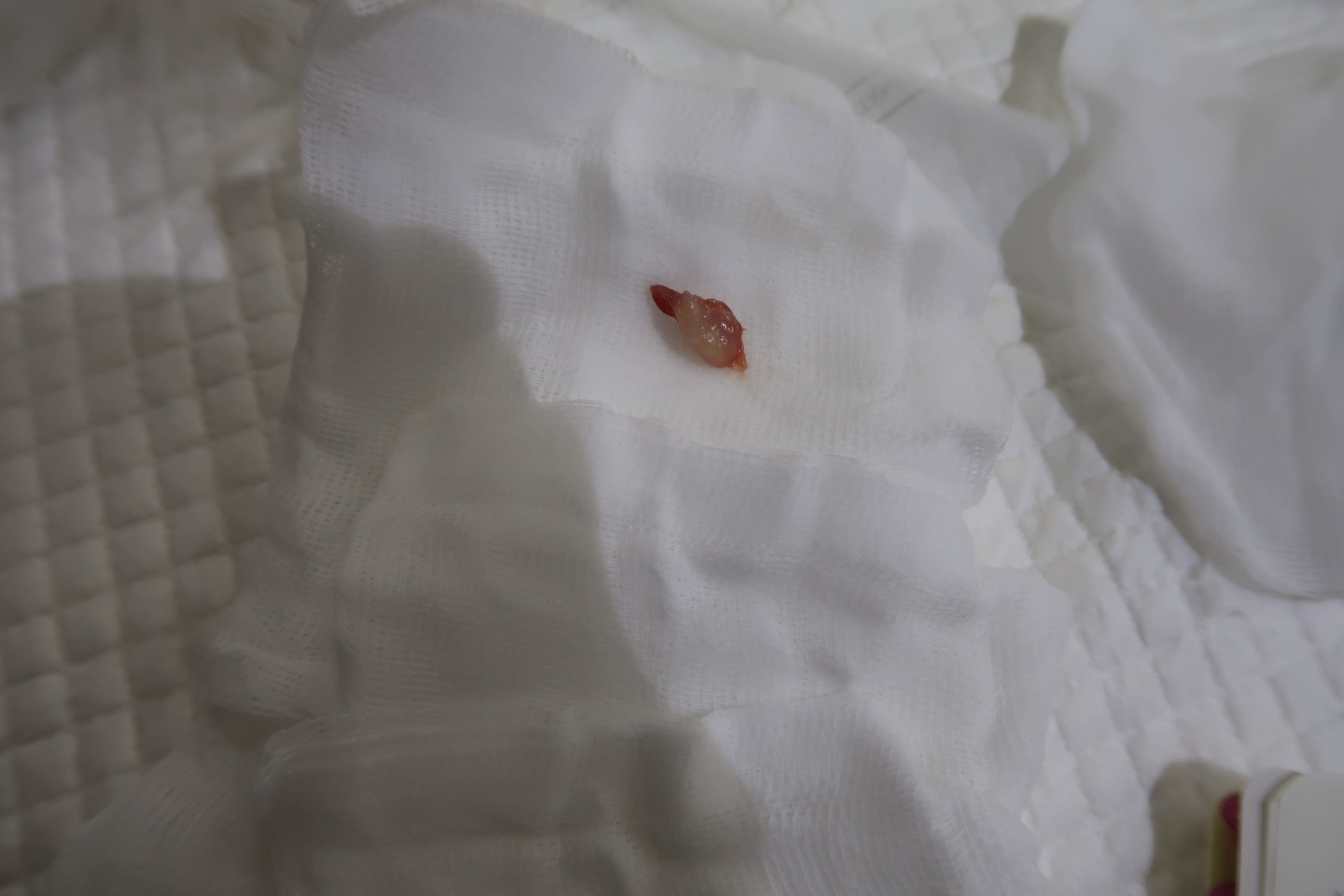

Supplement: Supplementary file 1 [file vetsci-12-01045-s001.zip › KakaoTalk_20221022_164754286_08.jpg]

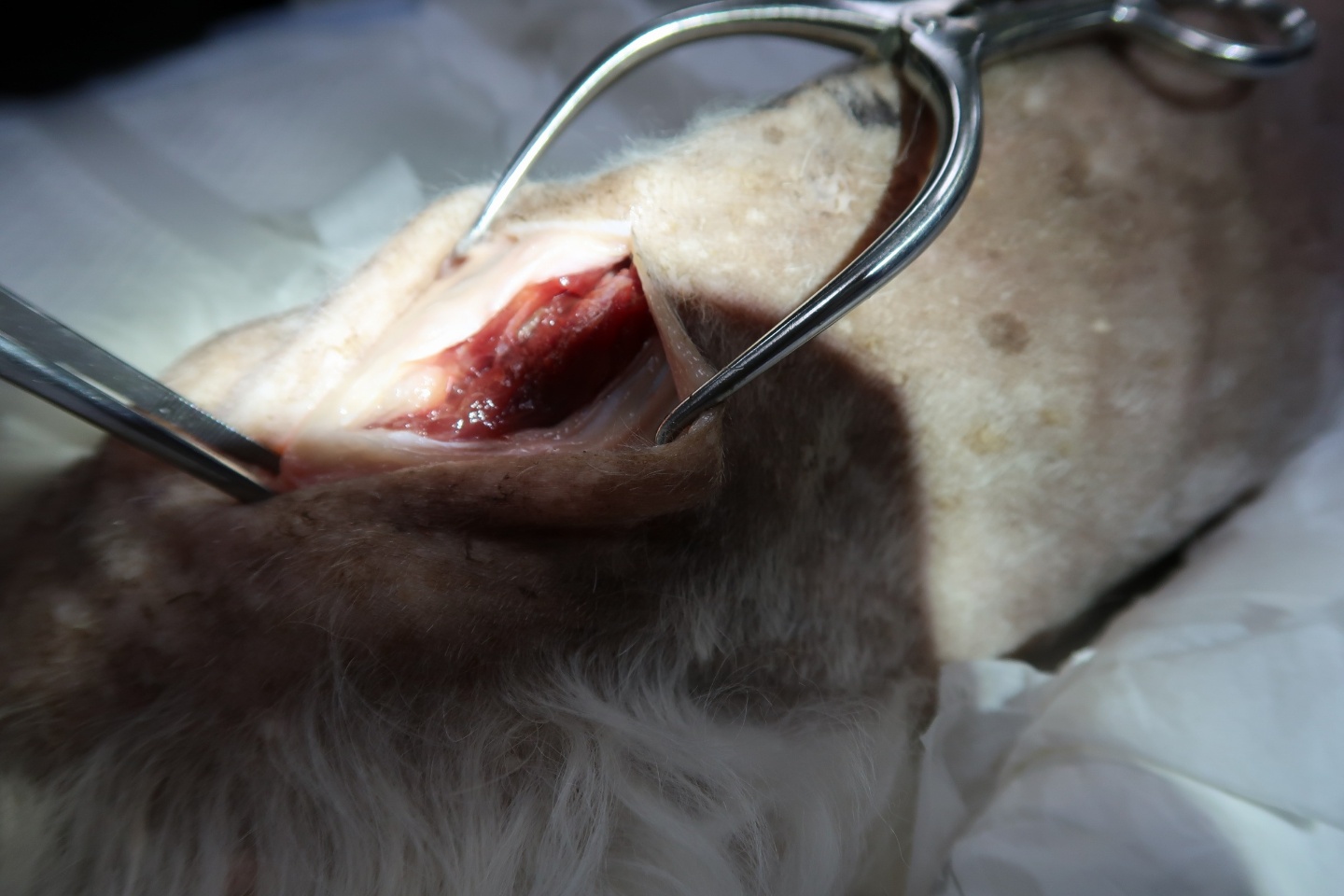

Supplement: Supplementary file 1 [file vetsci-12-01045-s001.zip › KakaoTalk_20221022_164754286_09.jpg]

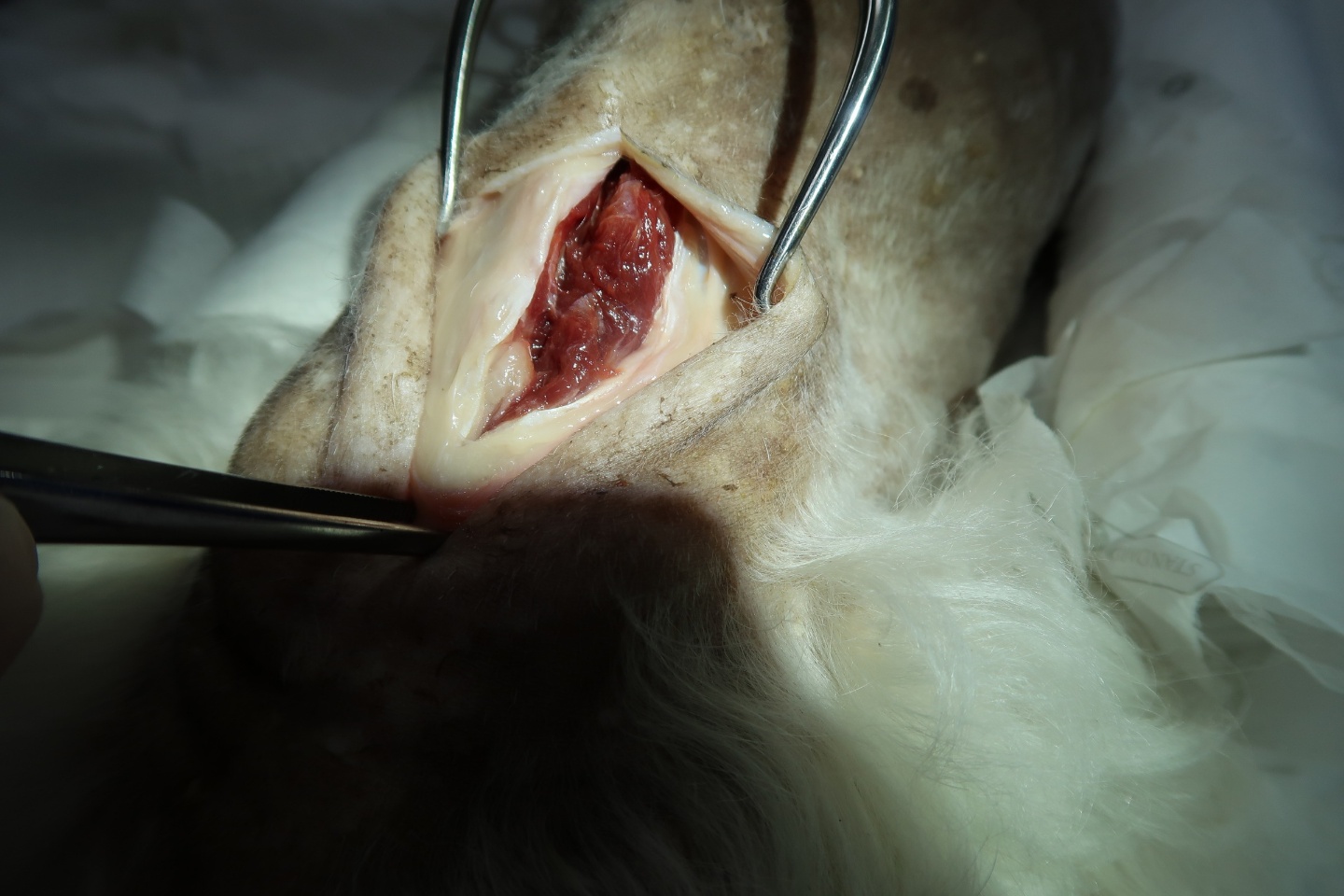

Supplement: Supplementary file 1 [file vetsci-12-01045-s001.zip › KakaoTalk_20221022_164754286_10.jpg]

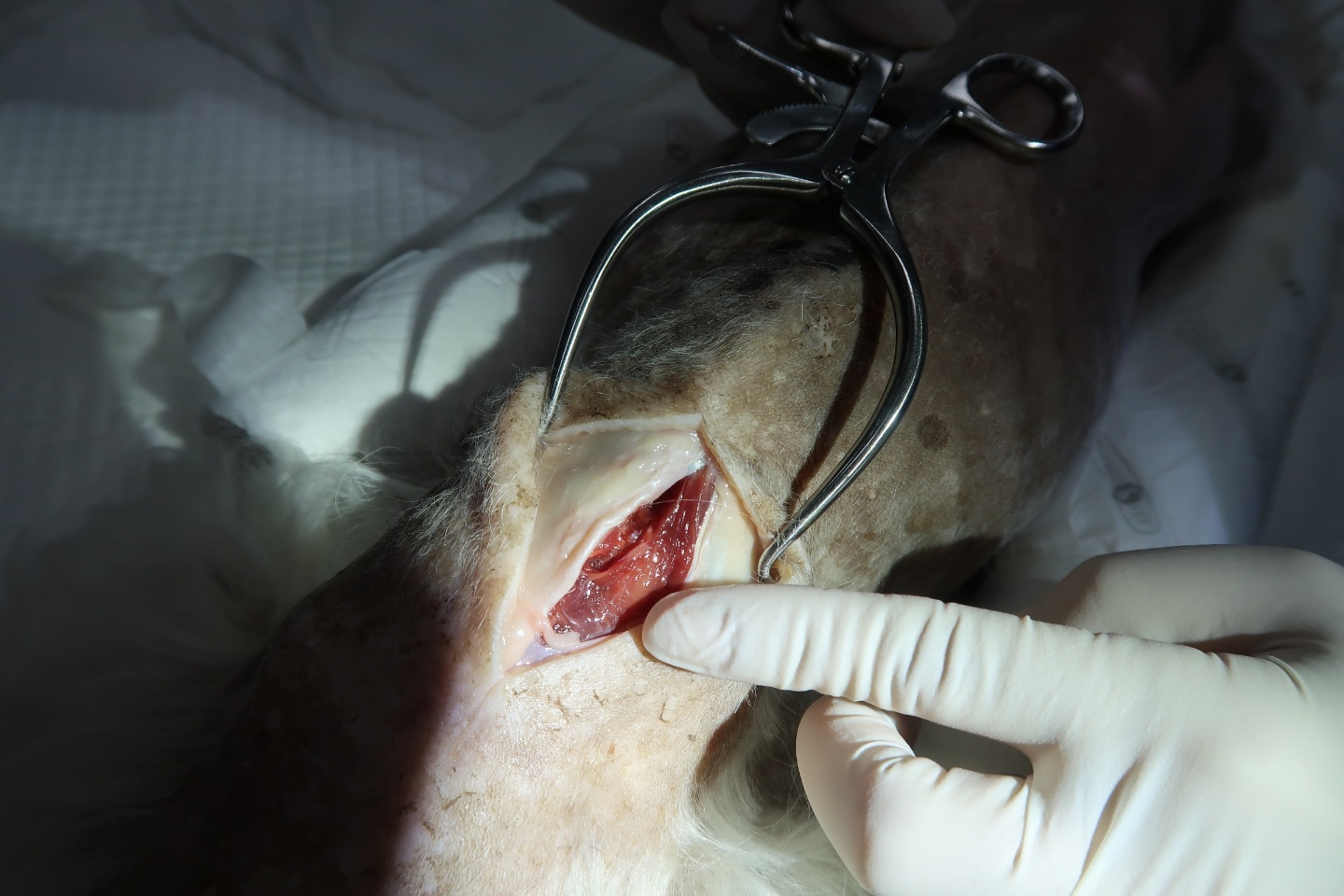

Supplement: Supplementary file 1 [file vetsci-12-01045-s001.zip › KakaoTalk_20221022_164754286_11.jpg]

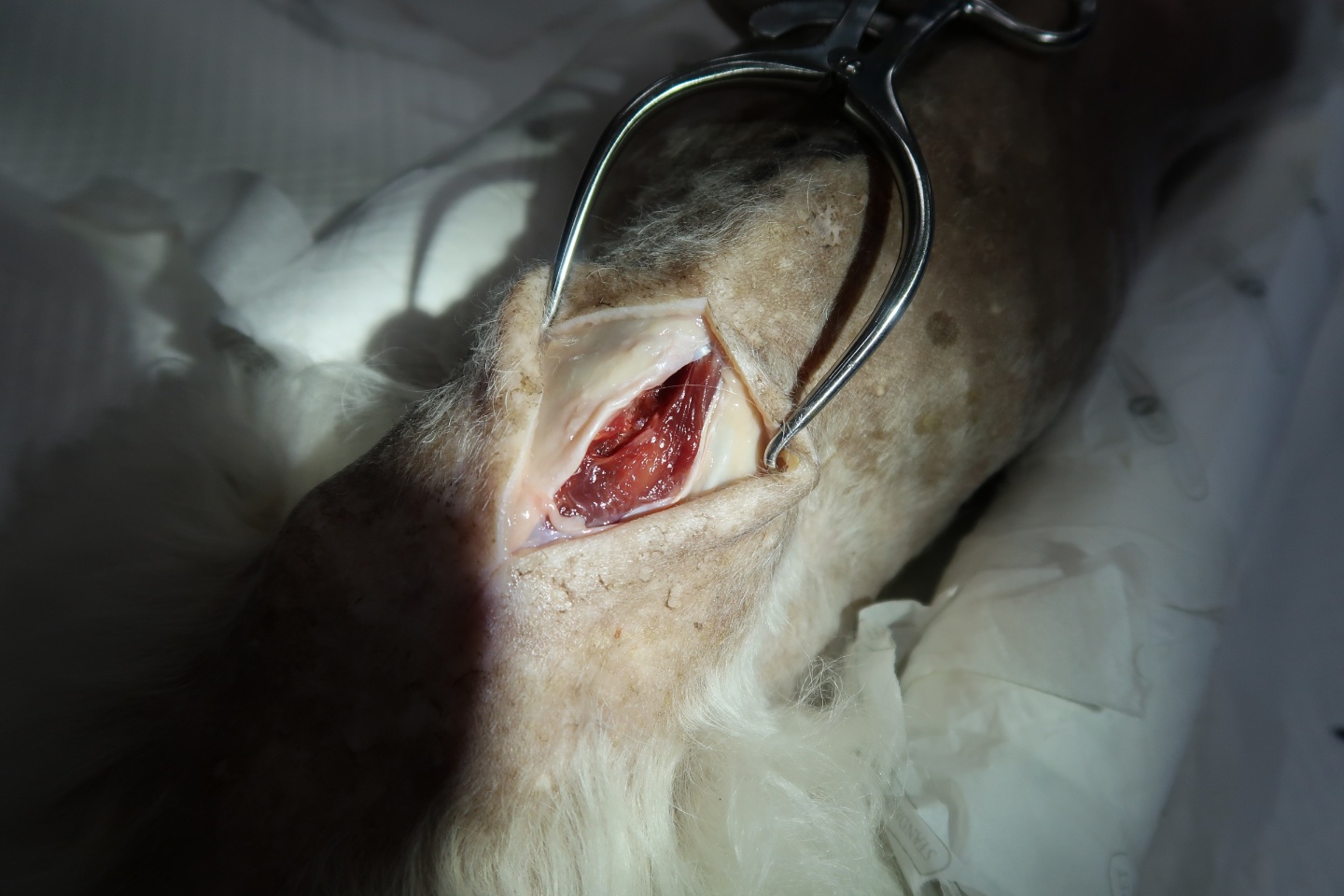

Supplement: Supplementary file 1 [file vetsci-12-01045-s001.zip › KakaoTalk_20221022_164754286_12.jpg]

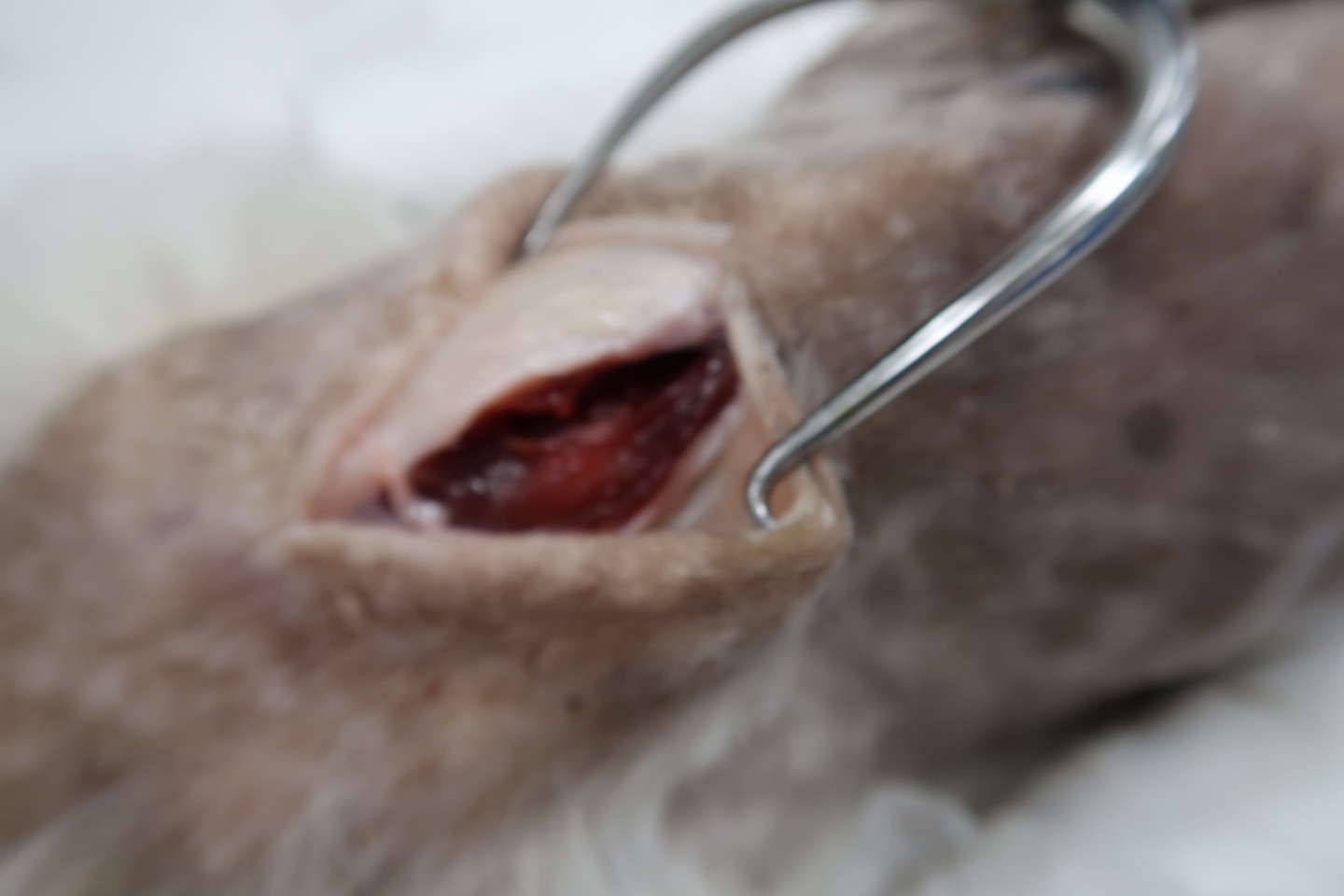

Supplement: Supplementary file 1 [file vetsci-12-01045-s001.zip › KakaoTalk_20221022_164754286_13.jpg]

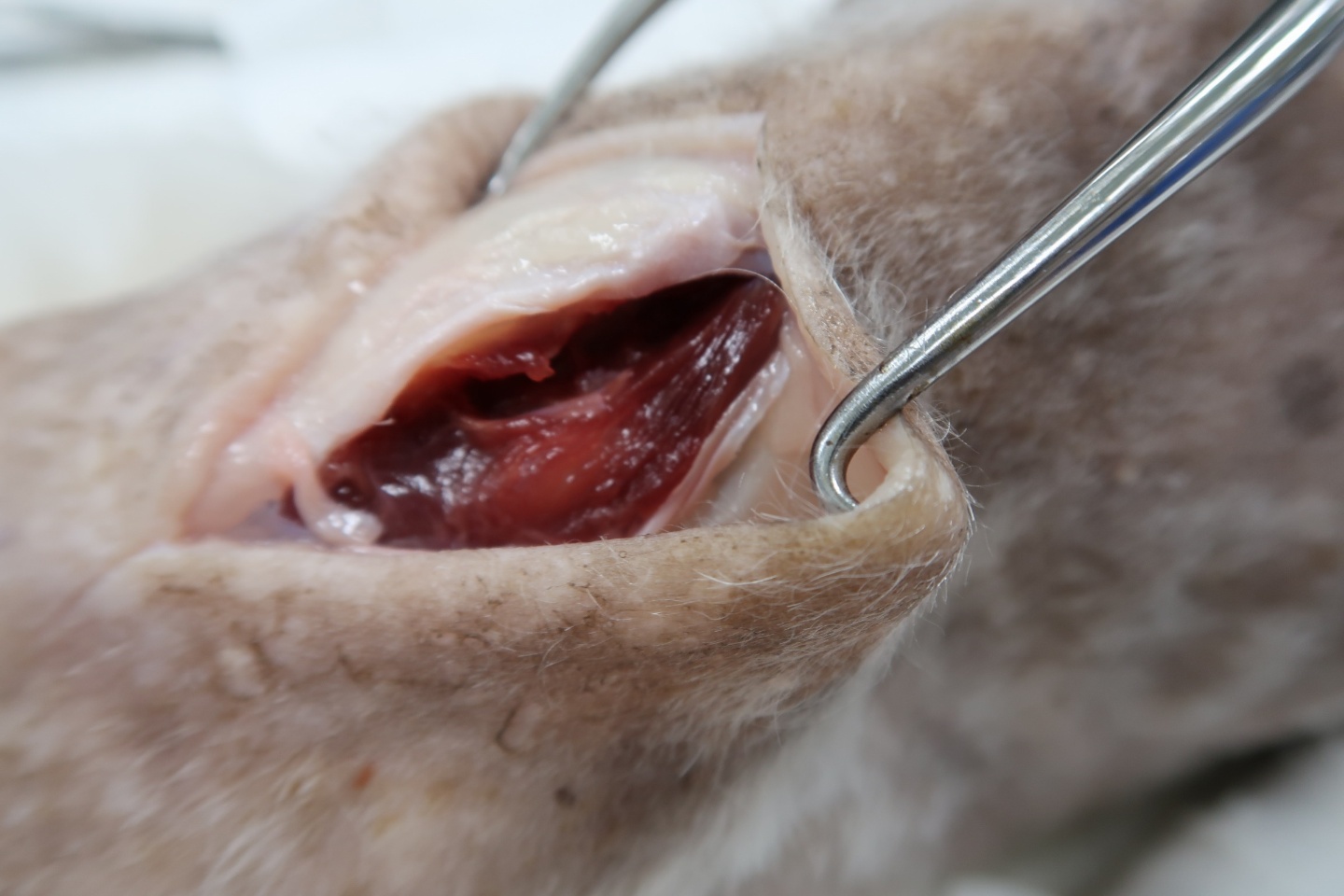

Supplement: Supplementary file 1 [file vetsci-12-01045-s001.zip › KakaoTalk_20221022_164754286_14.jpg]

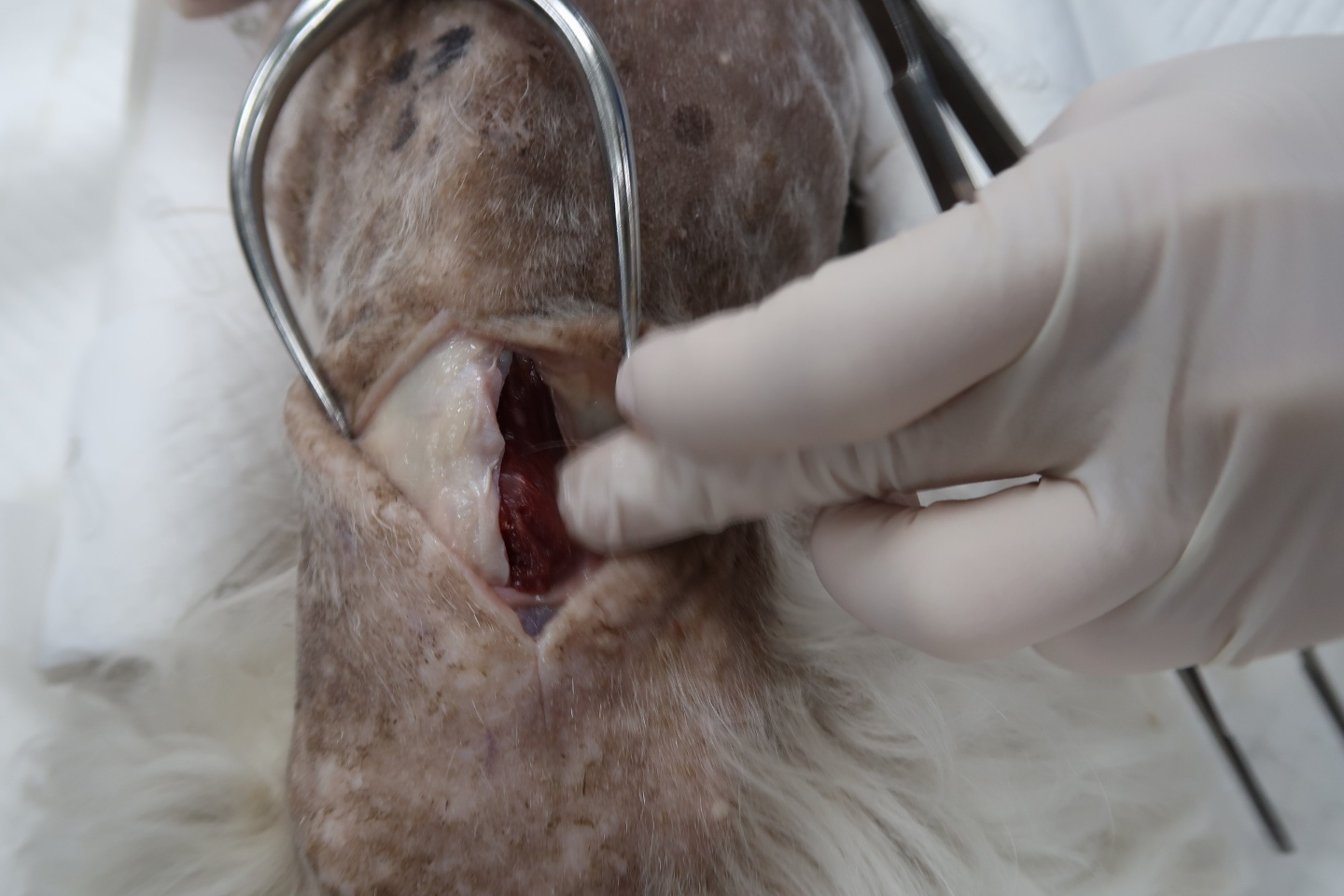

Supplement: Supplementary file 1 [file vetsci-12-01045-s001.zip › KakaoTalk_20221022_164754286_15.jpg]

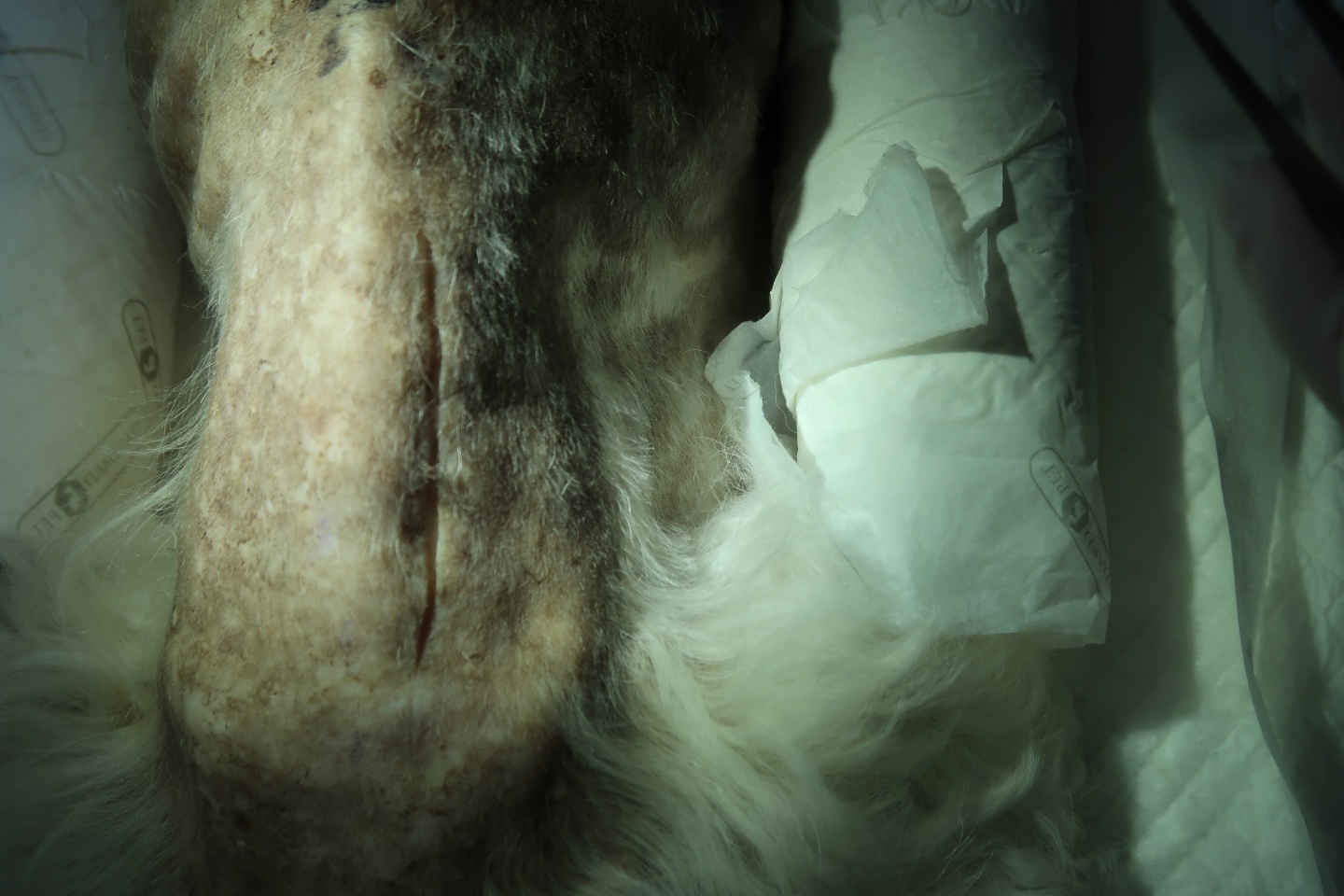

Supplement: Supplementary file 1 [file vetsci-12-01045-s001.zip › KakaoTalk_20221022_164754286_16.jpg]

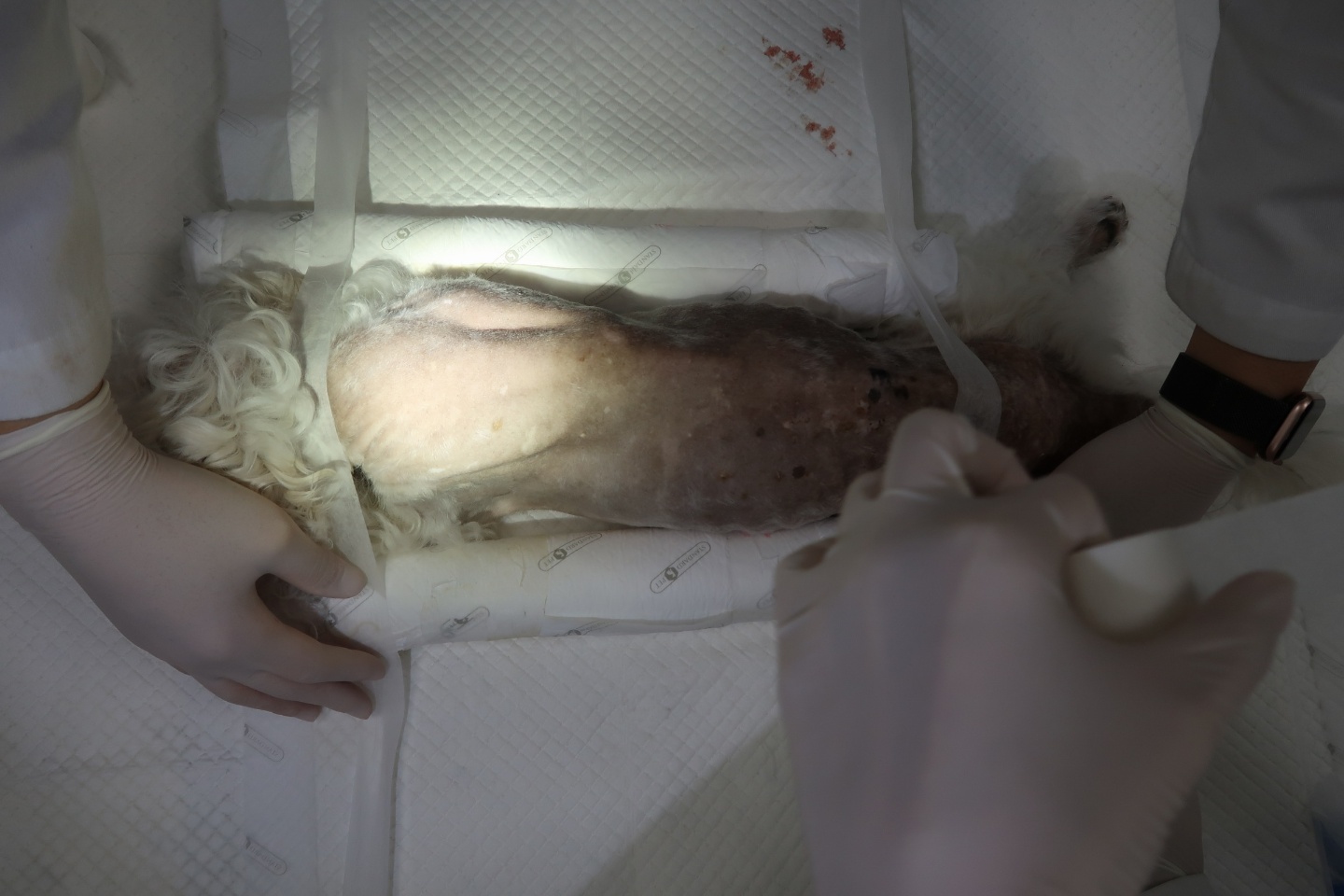

Supplement: Supplementary file 1 [file vetsci-12-01045-s001.zip › KakaoTalk_20221022_164754286_17.jpg]

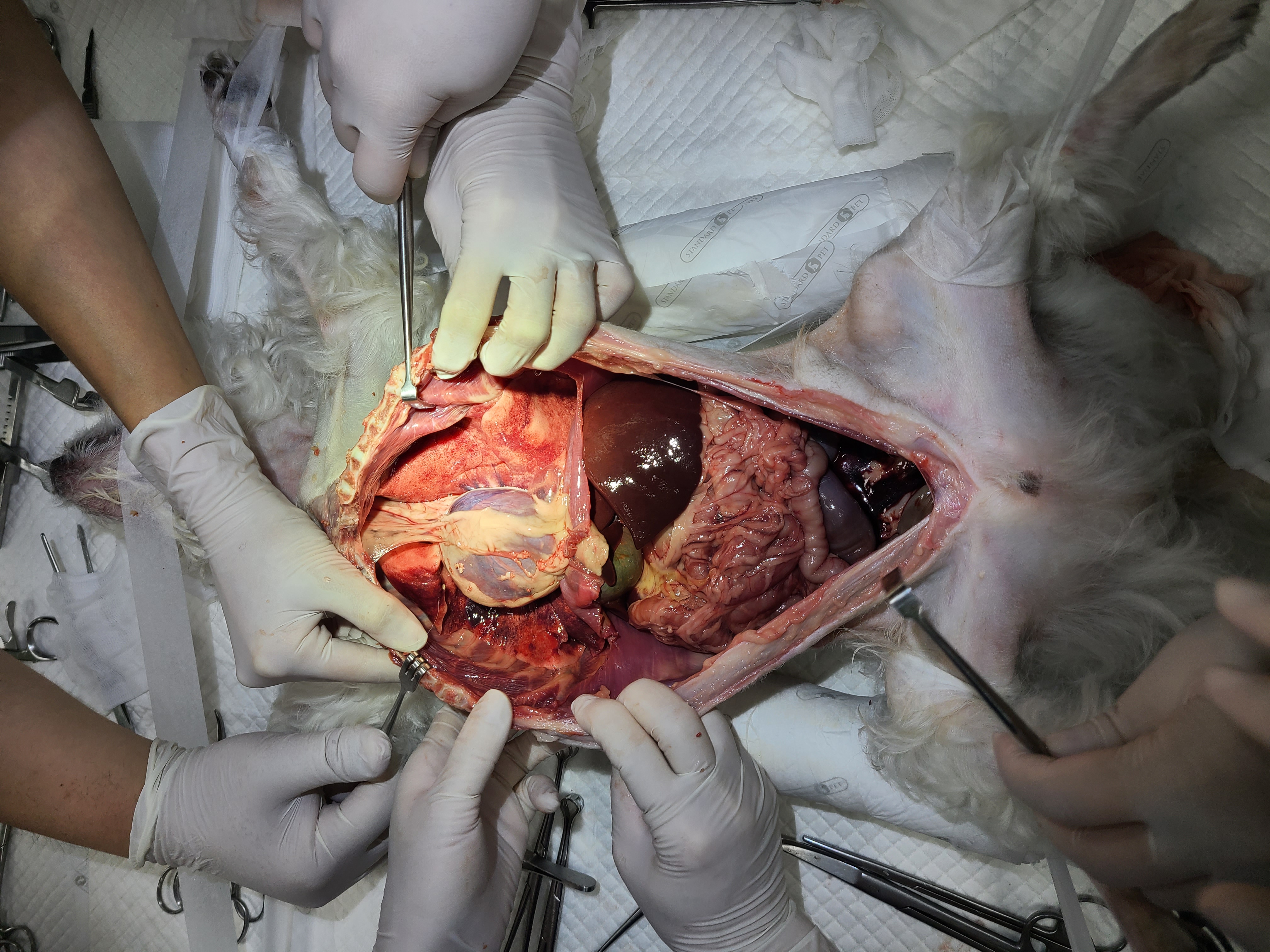

Supplement: Supplementary file 1 [file vetsci-12-01045-s001.zip › KakaoTalk_20221022_173552299.jpg]

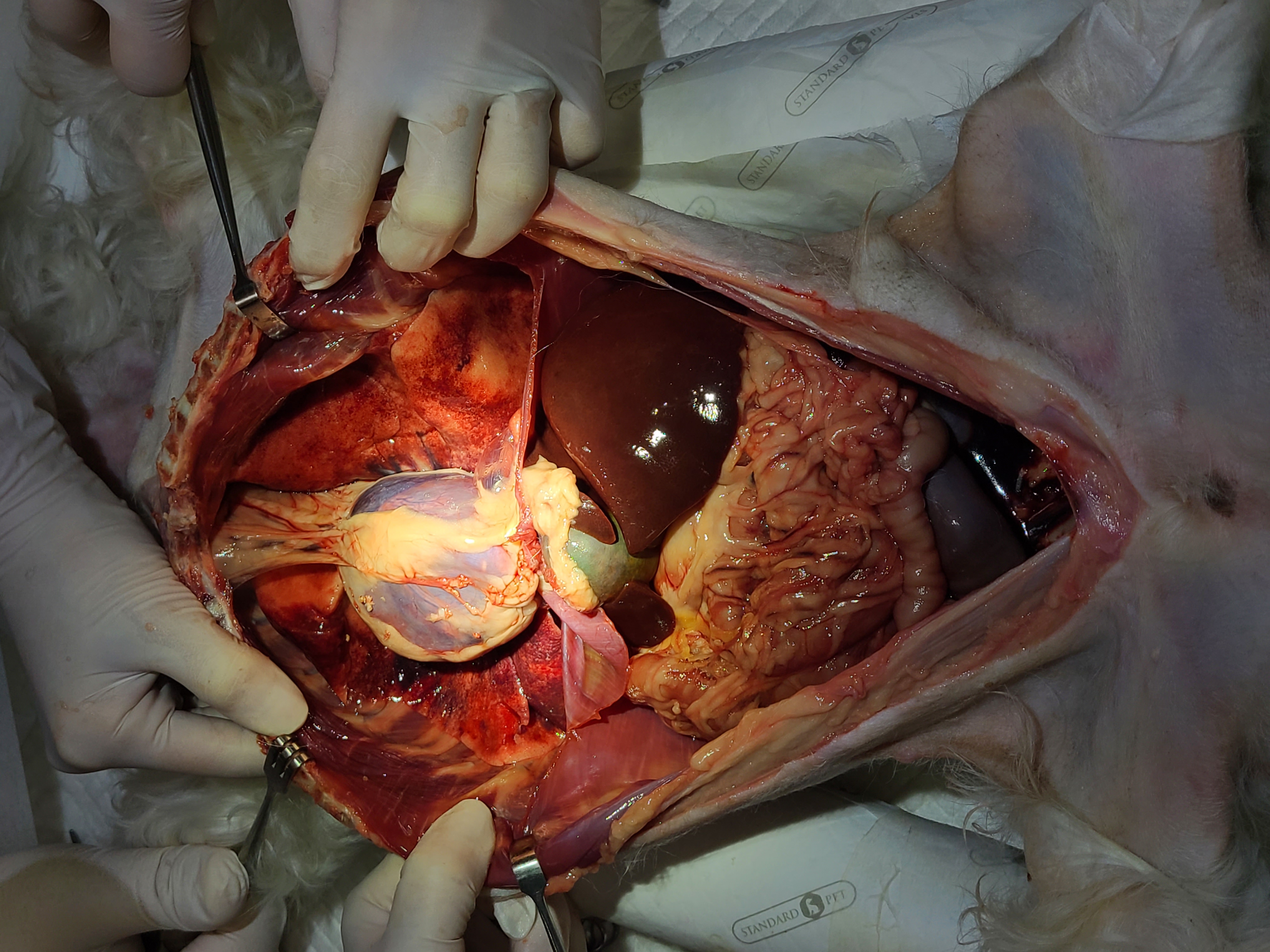

Supplement: Supplementary file 1 [file vetsci-12-01045-s001.zip › KakaoTalk_20221022_173552299_01.jpg]

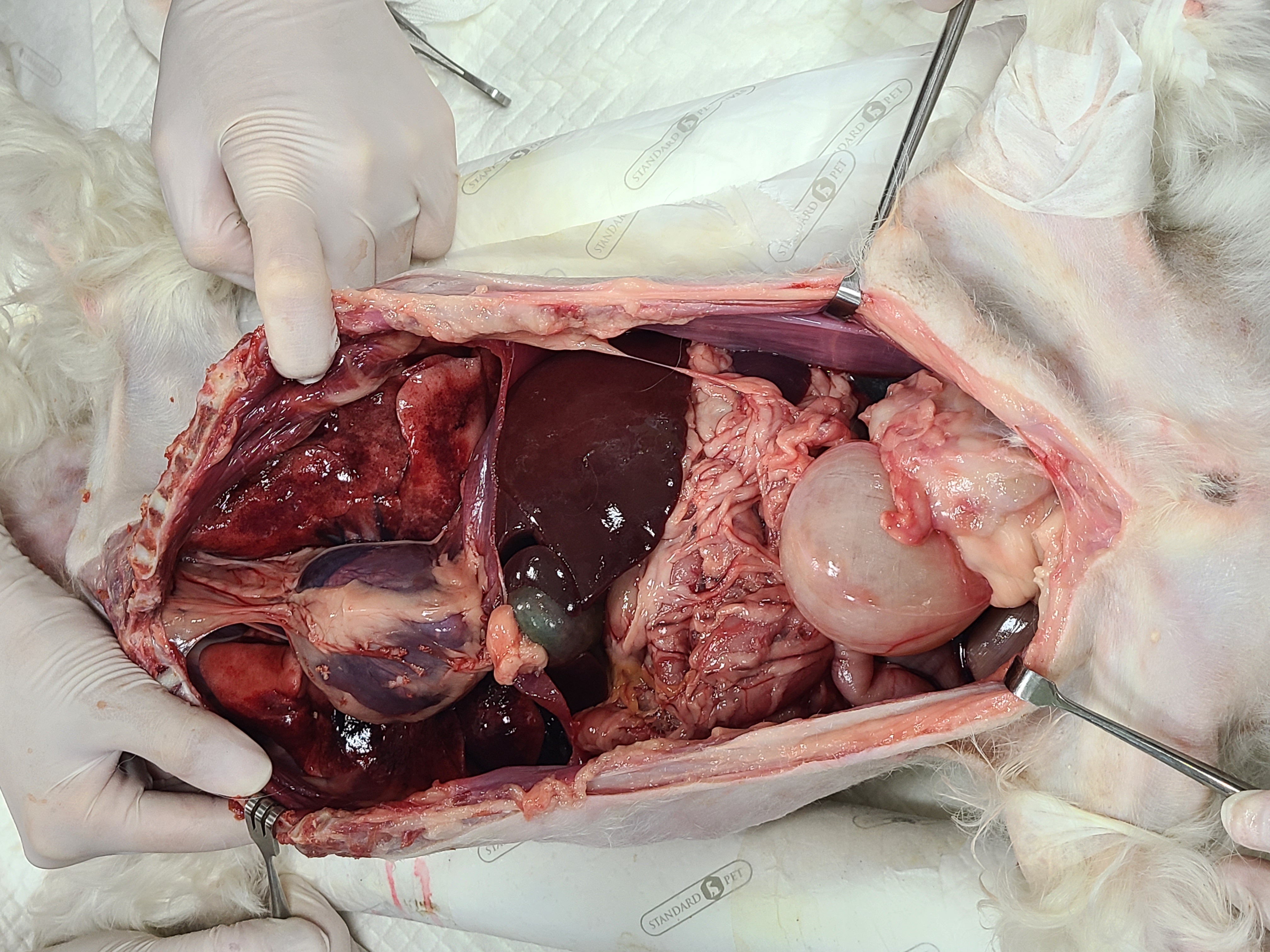

Supplement: Supplementary file 1 [file vetsci-12-01045-s001.zip › KakaoTalk_20221022_173552299_02.jpg]

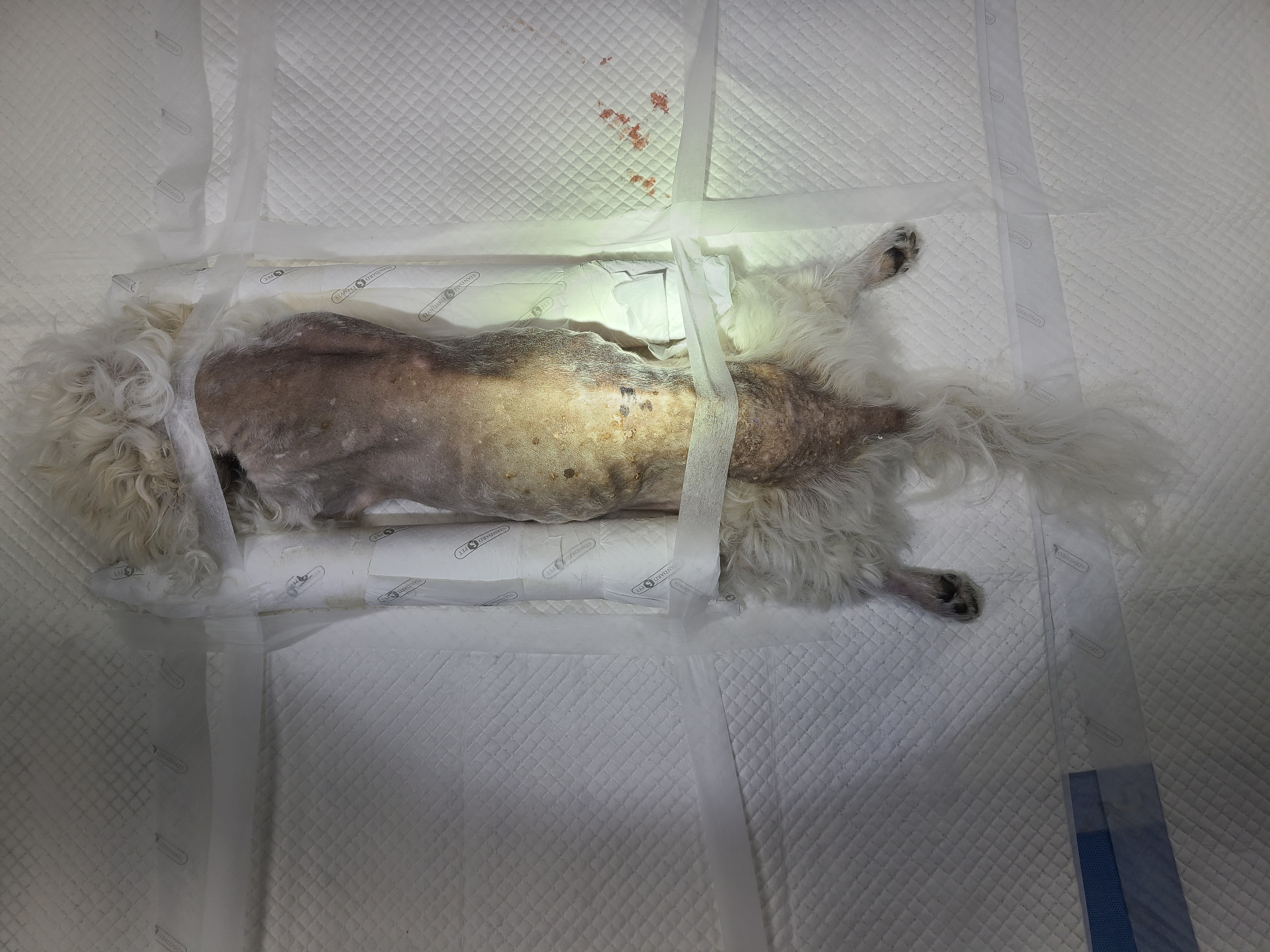

Supplement: Supplementary file 1 [file vetsci-12-01045-s001.zip › KakaoTalk_20221022_173638675.jpg]

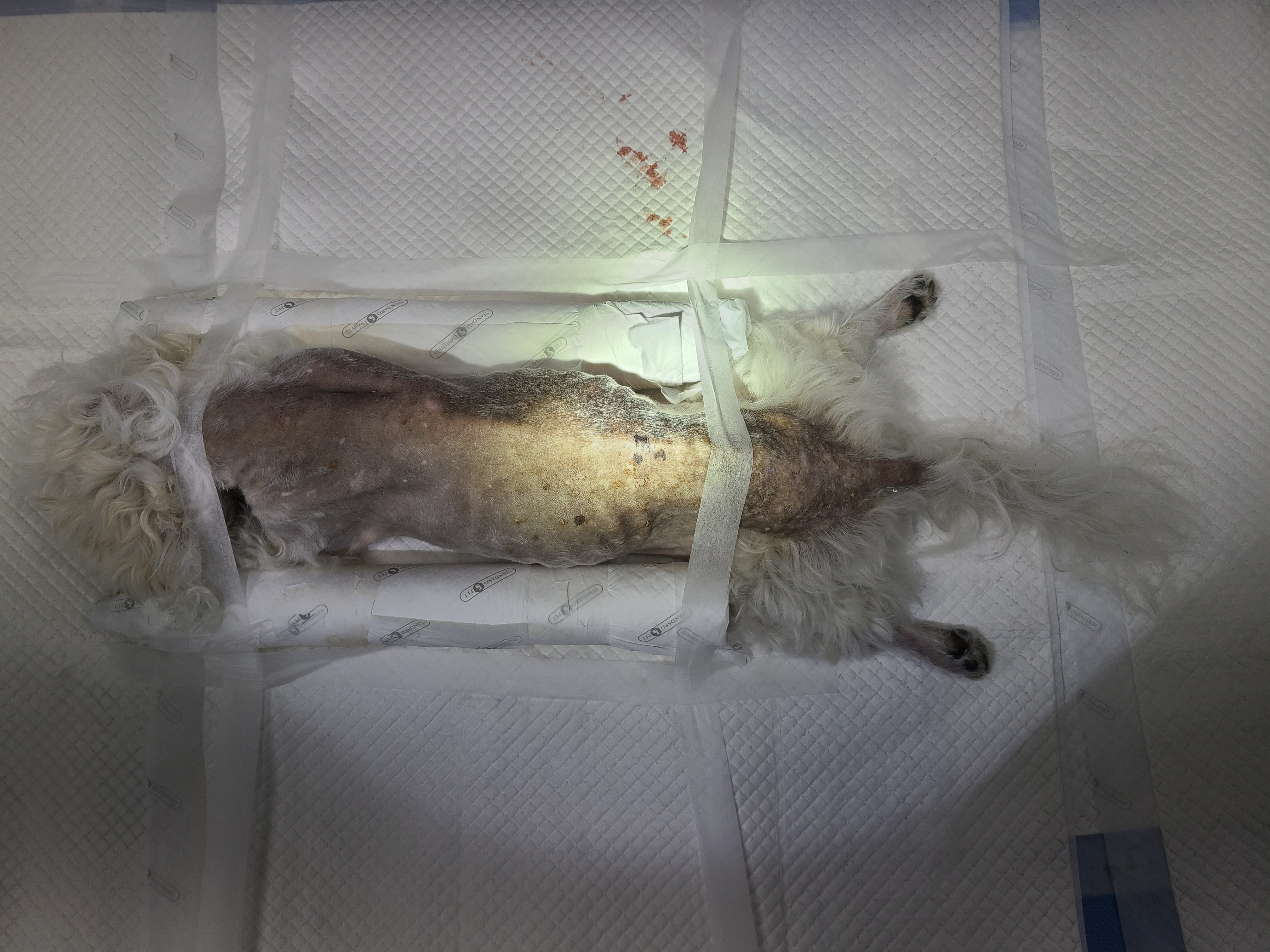

Supplement: Supplementary file 1 [file vetsci-12-01045-s001.zip › KakaoTalk_20221022_173638675_01.jpg]

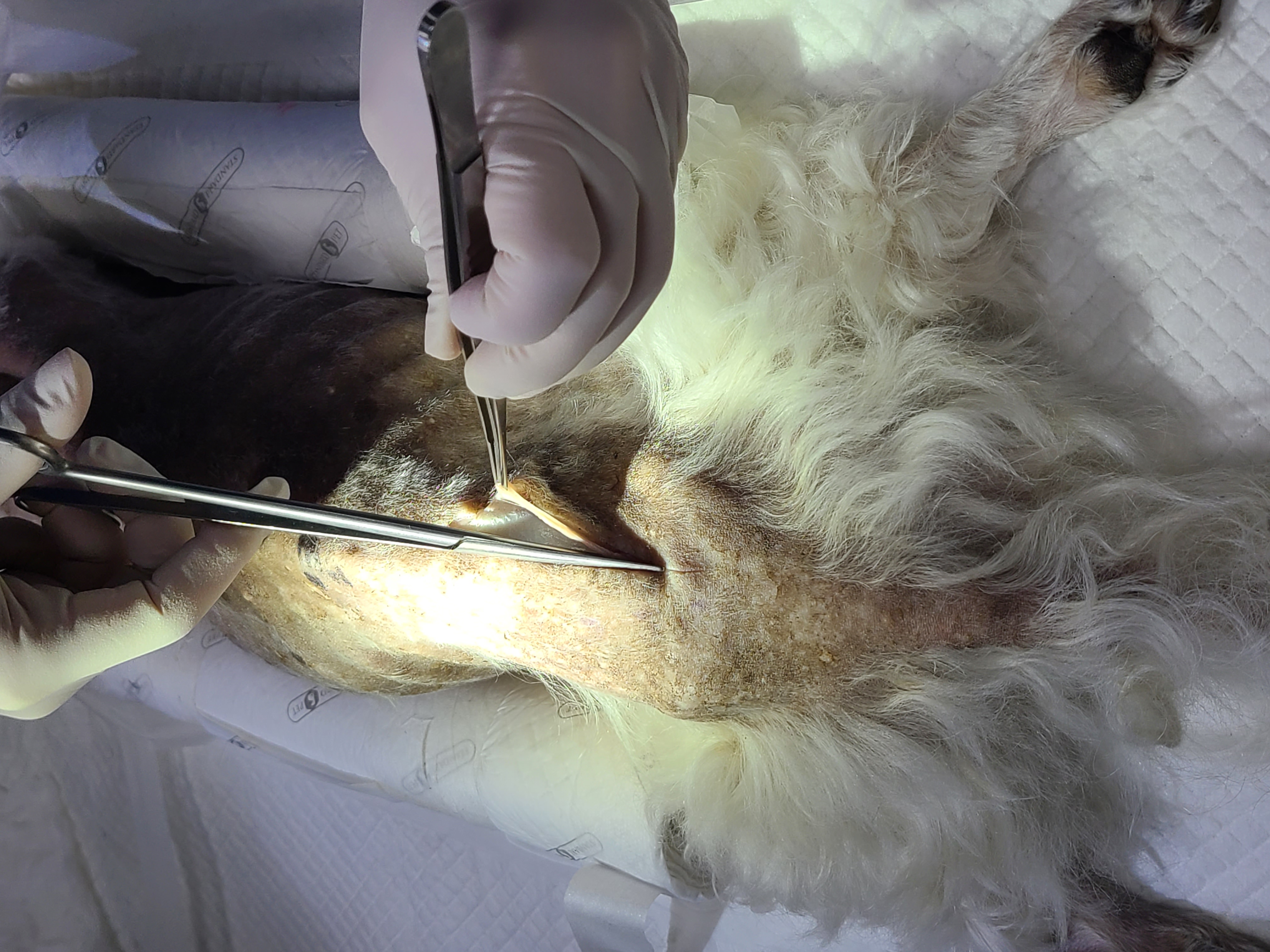

Supplement: Supplementary file 1 [file vetsci-12-01045-s001.zip › KakaoTalk_20221022_173638675_02.jpg]

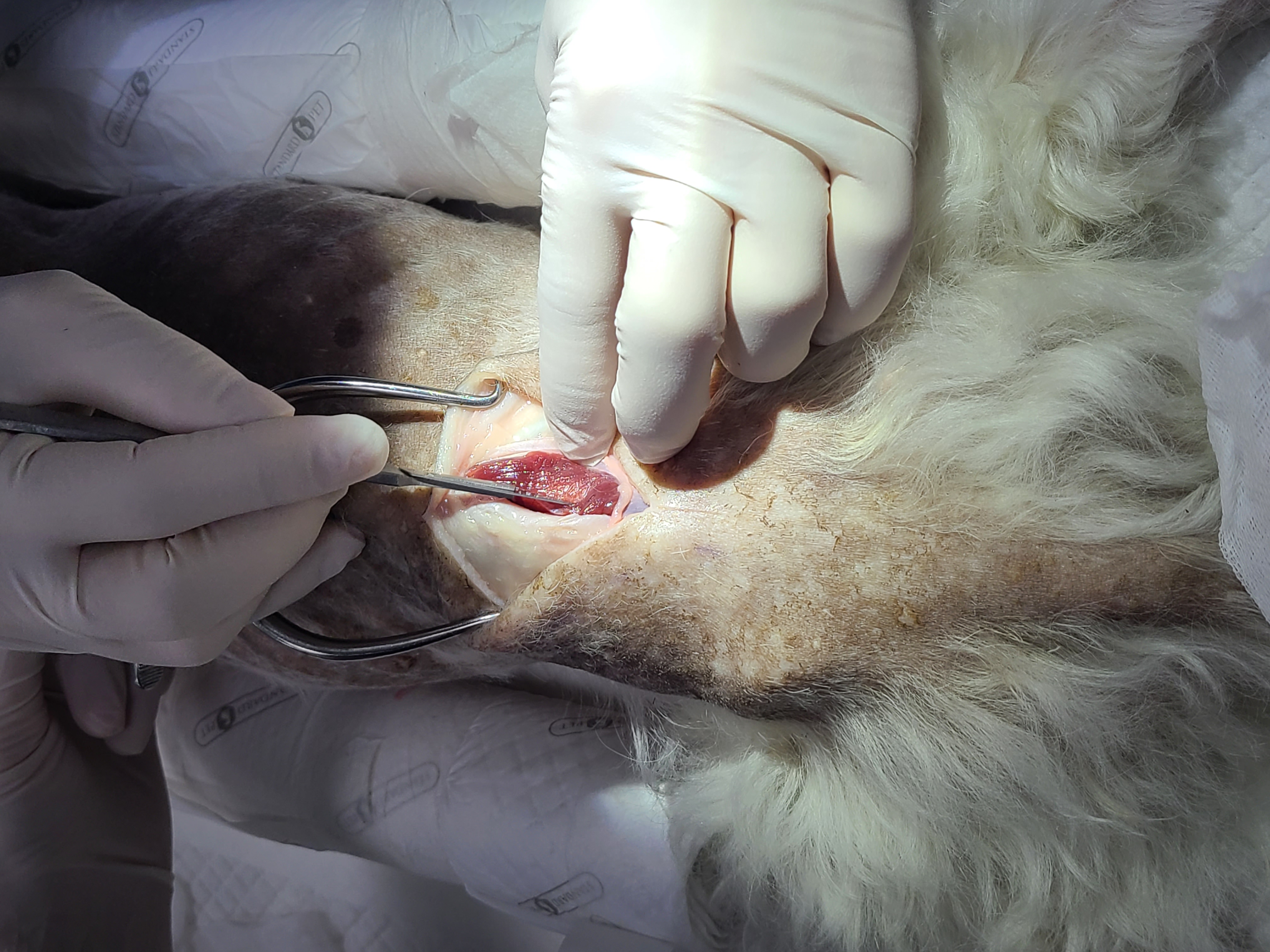

Supplement: Supplementary file 1 [file vetsci-12-01045-s001.zip › KakaoTalk_20221022_173638675_03.jpg]

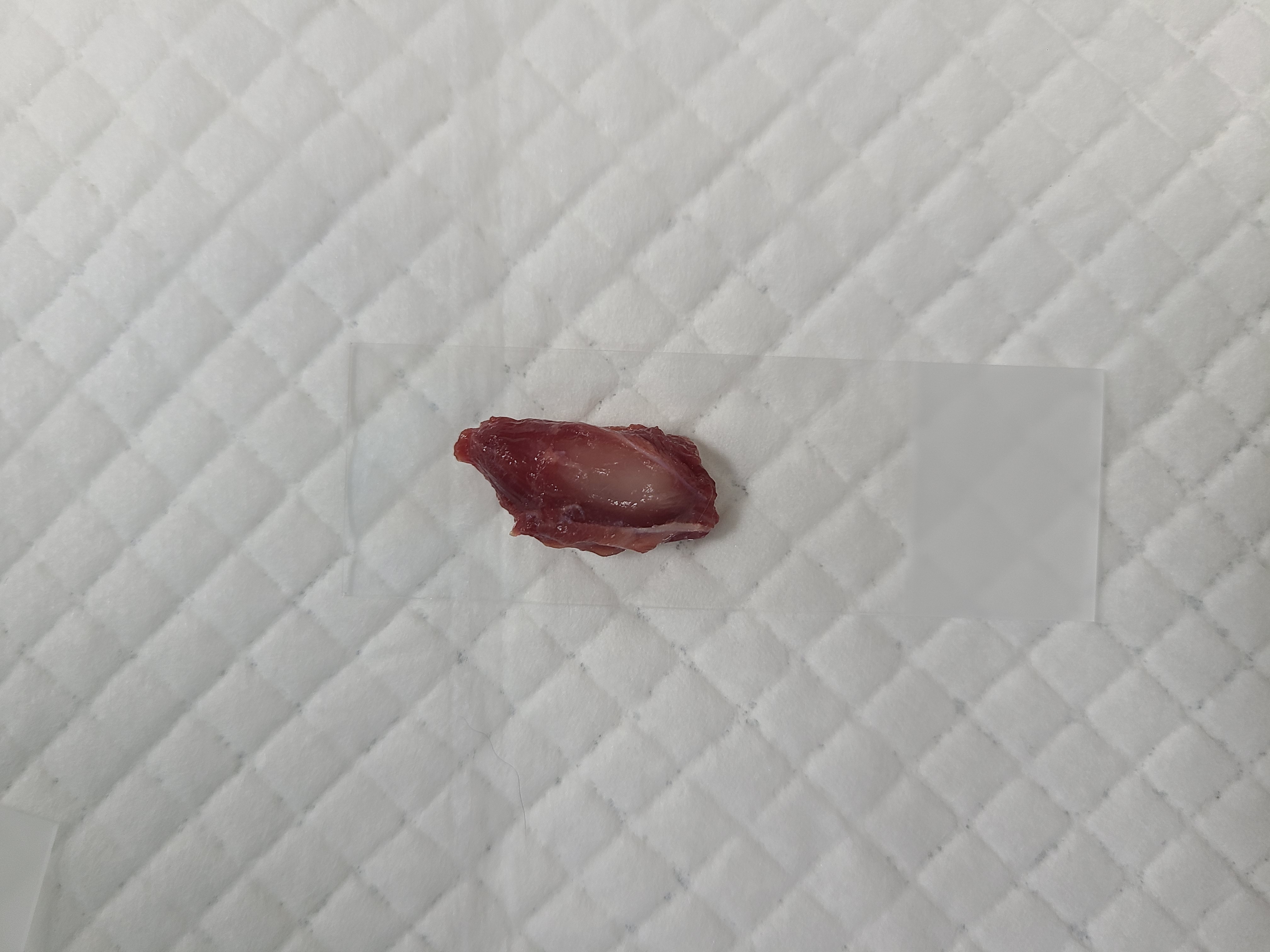

Supplement: Supplementary file 1 [file vetsci-12-01045-s001.zip › KakaoTalk_20221022_173638675_04.jpg]

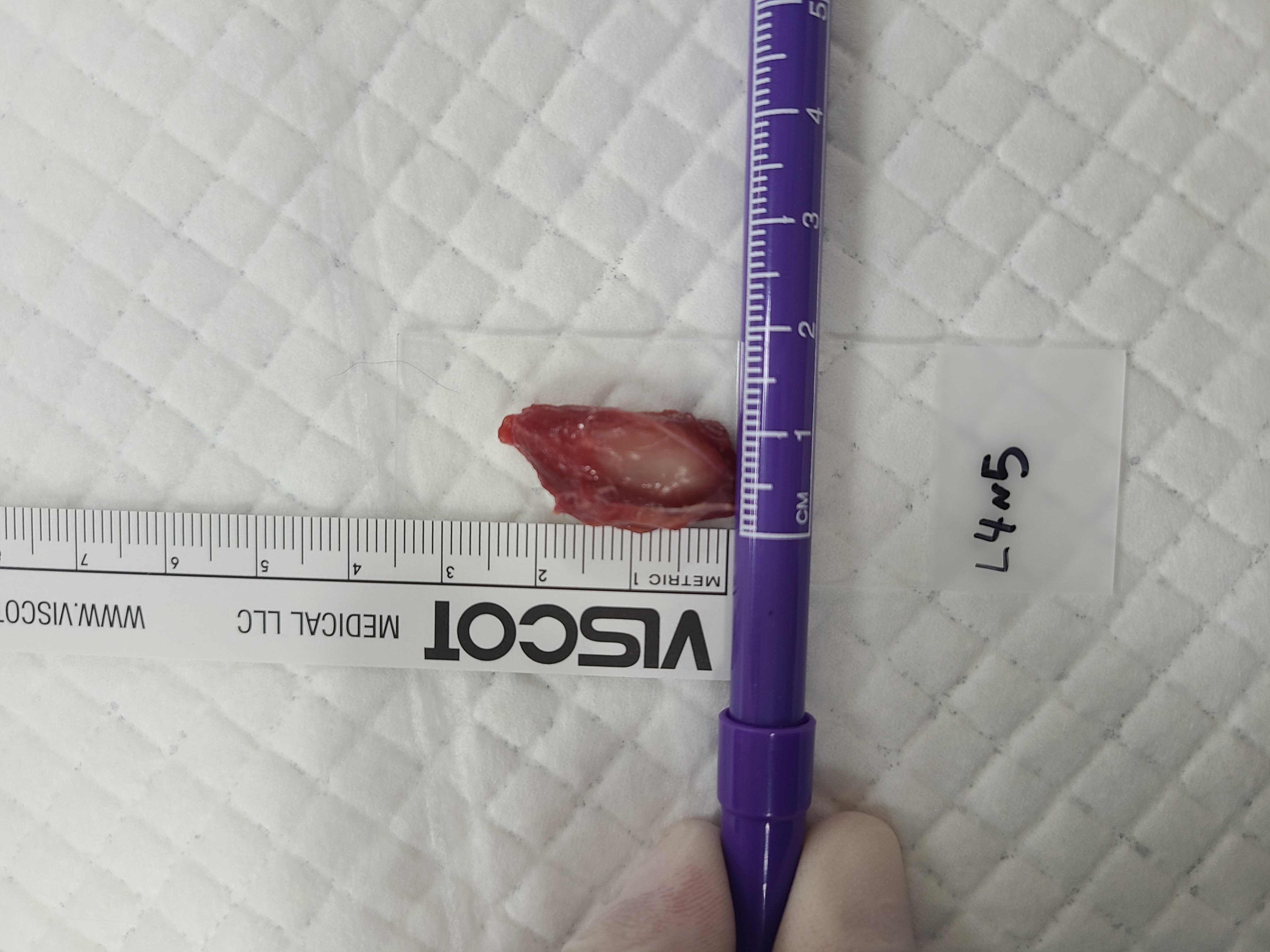

Supplement: Supplementary file 1 [file vetsci-12-01045-s001.zip › KakaoTalk_20221022_173638675_05.jpg]

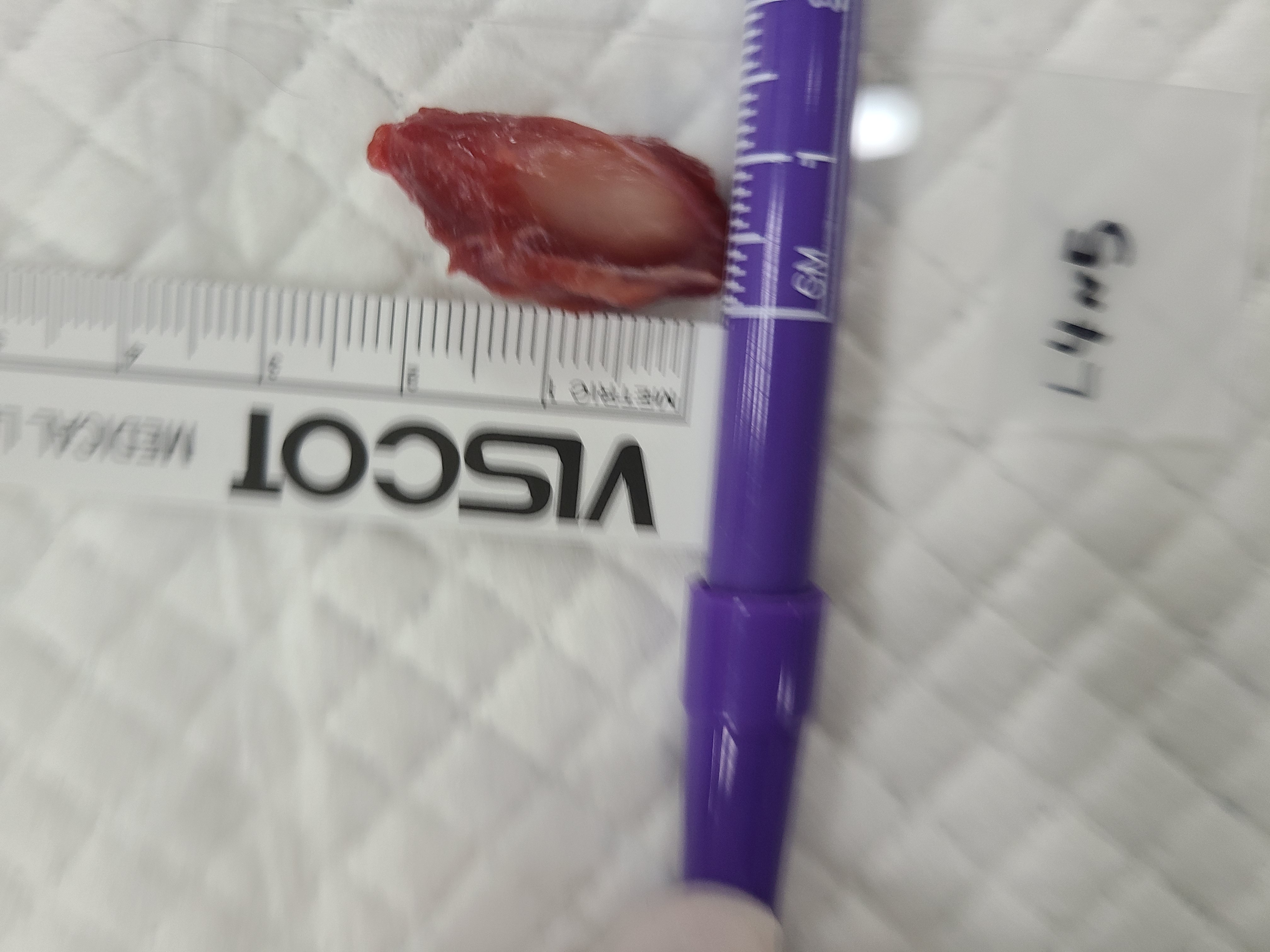

Supplement: Supplementary file 1 [file vetsci-12-01045-s001.zip › KakaoTalk_20221022_173638675_06.jpg]

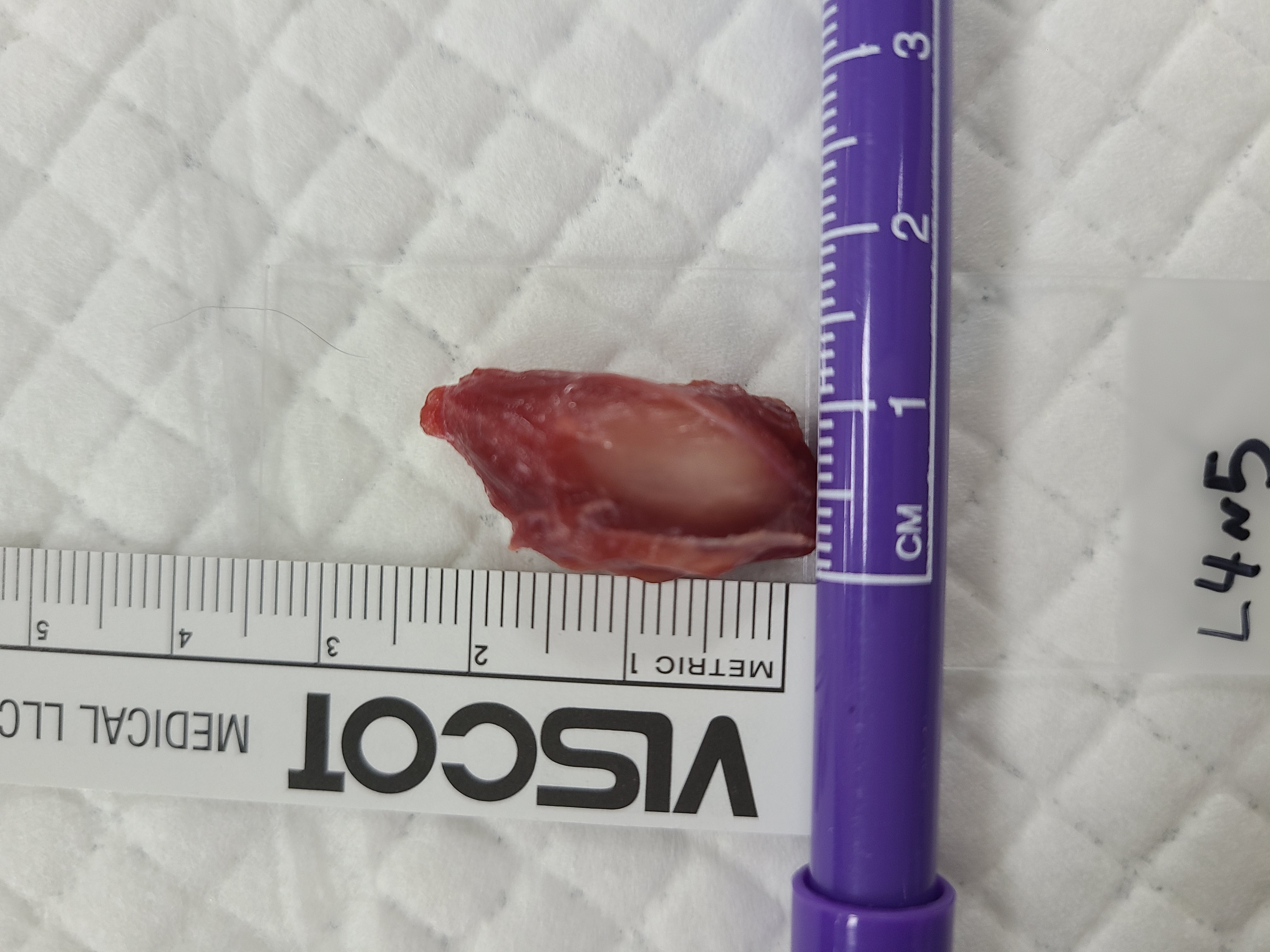

Supplement: Supplementary file 1 [file vetsci-12-01045-s001.zip › KakaoTalk_20221022_173638675_07.jpg]

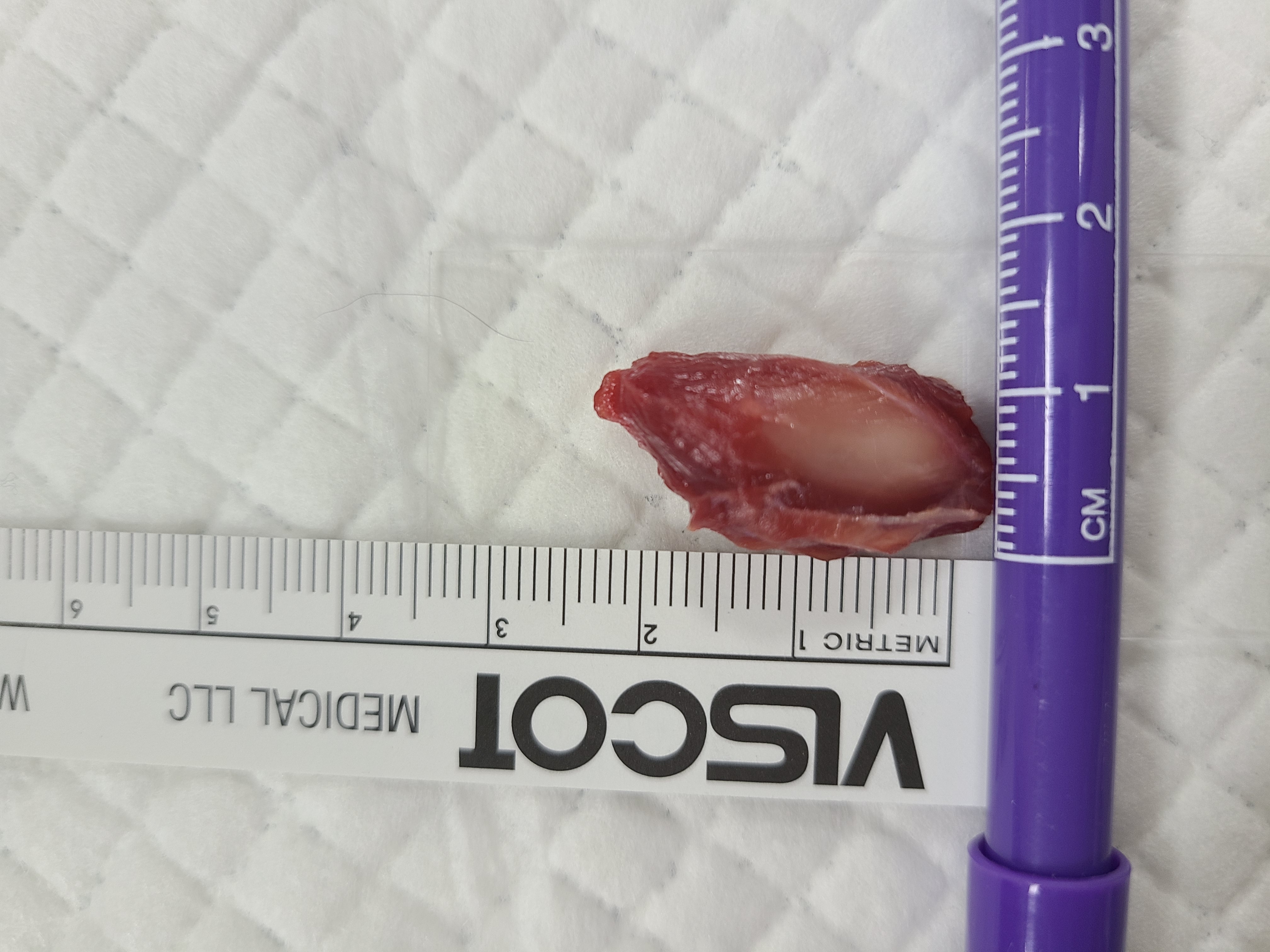

Supplement: Supplementary file 1 [file vetsci-12-01045-s001.zip › KakaoTalk_20221022_173638675_08.jpg]

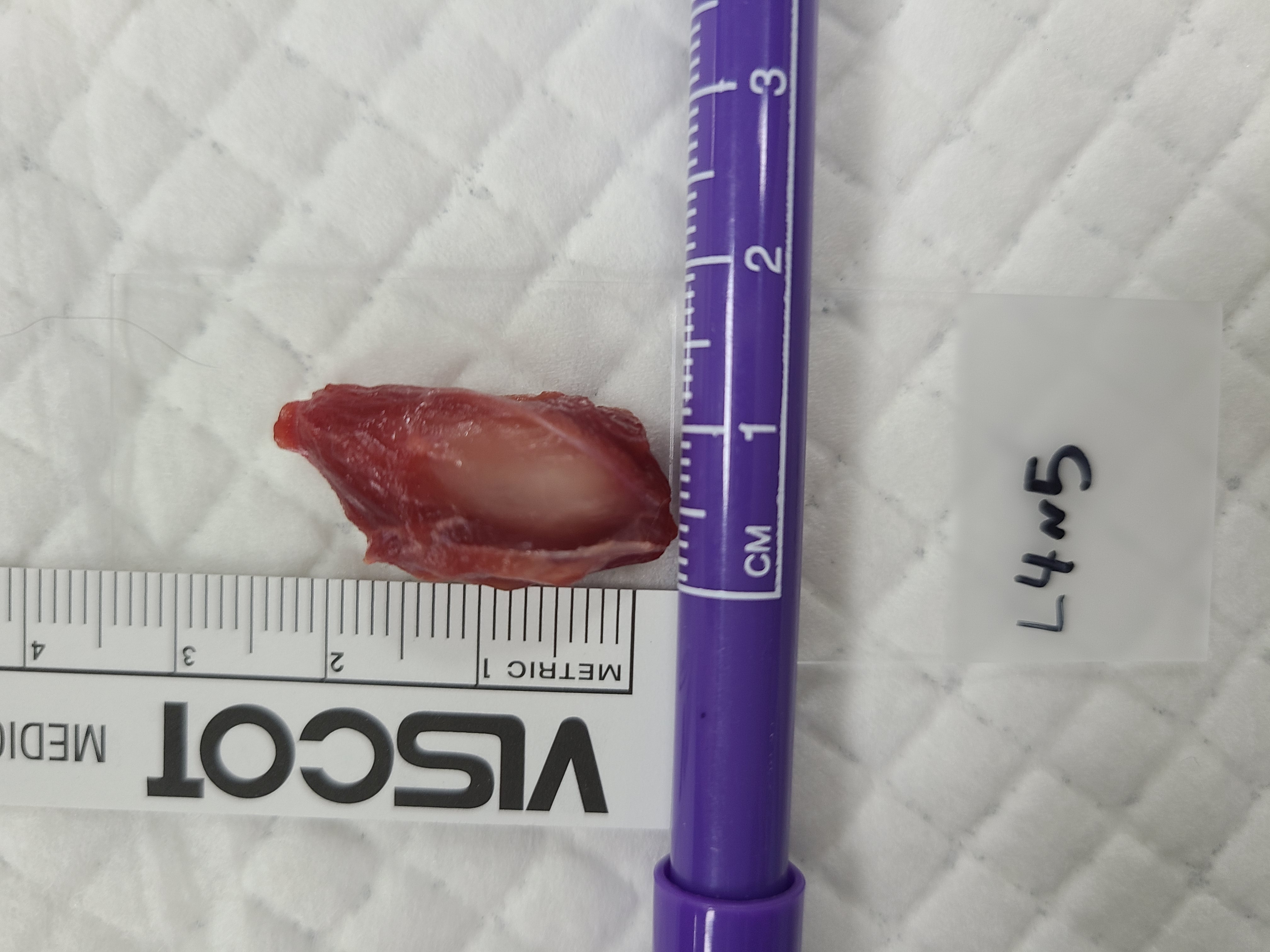

Supplement: Supplementary file 1 [file vetsci-12-01045-s001.zip › KakaoTalk_20221022_173638675_09.jpg]
